# The Protective Potential of *Aronia melanocarpa* L. Berry Extract against Cadmium-Induced Kidney Damage: A Study in an Animal Model of Human Environmental Exposure to This Toxic Element

**DOI:** 10.3390/ijms241411647

**Published:** 2023-07-19

**Authors:** Nazar M. Smereczański, Małgorzata M. Brzóska, Joanna Rogalska, Tomasz Hutsch

**Affiliations:** 1 Department of Toxicology, Medical University of Bialystok, Adama Mickiewicza 2C Street, 15-222 Bialystok, Poland; 2 Department of Pathology and Veterinary Diagnostics, Institute of Veterinary Medicine, Warsaw University of Life Sciences, Nowoursynowska 159C Street, 02-776 Warsaw, Poland; 3 Veterinary Diagnostic Laboratory ALAB Bioscience, Stępińska 22/30 Street, 00-739 Warsaw, Poland

**Keywords:** cadmium, kidney, nephrotoxicity, tubular damage, glomerular damage, biomarkers, histopathology, *Aronia melanocarpa* berry extract, protection

## Abstract

The impact of cadmium (Cd) on the function and structure of the kidney and the potential protective effect of an extract from *Aronia melanocarpa* L. berries were investigated in a rat model of low- and moderate-level environmental exposure to this heavy metal (1 and 5 mg Cd/kg feed for up to 24 months). The sensitive biomarkers of Cd-induced damage to the kidney tubules (N-acetyl-β-D-glucosaminidase (NAG), alkaline phosphatase (ALP), β2-microglobulin (β2-MG), and kidney injury molecule-1 (KIM-1) in the urine), clinically relevant early markers of glomerular damage (albumin in the urine and creatinine clearance), and other markers of the general functional status of this organ (urea, uric acid, and total protein in the serum and/or urine) and Cd concentration in the urine, were evaluated. The morphological structure of the kidney and inflammatory markers (chemerin, macrophage inflammatory protein 1 alpha (MIP1a), and Bcl2-associated X protein (Bax)) were also estimated. Low-level and moderate exposure to Cd led to damage to the function and structure of the kidney tubules and glomeruli. The co-administration of *A. melanocarpa* berry extract significantly protected against the injurious impact of this toxic element. In conclusion, even low-level, long-term exposure to Cd poses a risk of kidney damage, whereas an intake of Aronia berry products may effectively protect from this outcome.

## 1. Introduction

A growing body of epidemiological data shows that environmental exposure to heavy metals, including cadmium (Cd), is now a real threat to public health in industrialized countries around the world and that the exposure is increasing [1,2]. Cd was ranked by the Agency for Toxic Substances and Disease Registry as the 7th most dangerous xenobiotic that endangers the health of the general population [3]. This results from the fact that environmental exposure to this heavy metal can lead to damage to numerous organs and systems, including the kidney [2,4,5,6] and liver [7,8], as well as the skeletal system [8,9], nervous system [8,10], and cardiovascular system [11].

The kidney is the main organ of Cd accumulation in the body and the first organ to be damaged (i.e., the target organ) under chronic intoxication with this heavy metal [1,2,8]. This xenobiotic has a damaging impact on the structure and function of the renal tubules and glomeruli [2,4,8,12,13,14]. Current global environmental exposure to Cd is generally low to moderate (except for areas considered excessively polluted). However, the results of epidemiological studies show that even such exposure may negatively affect the kidney [2,13,14,15,16]. The lowest observed adverse effect levels (LOAELs) of this element concentration for clinically relevant kidney damage (glomerular dysfunction) have been estimated to be >0.18 μg/L in the blood and >0.27 μg/g creatinine in the urine and are within the lower range of concentrations noted in inhabitants of industrialized countries [2,4,5,6,7,9,11,13,14]. These indicate that current levels of exposure of the general population to this heavy metal may pose a real risk of organ injury, but the risk is not well known so far (for a review, see [2]). That is why it is of high importance not only to recognize the risk but also to find an effective protective strategy. However, due to the probability of the coexistence of numerous factors that may influence kidney status in the general population, it is very difficult to evaluate the involvement of this xenobiotic in the development of organ damage under low-level exposure [2]. The impact may be well estimated in experimental models that allow for the exclusion of confounding factors. Such models are also appropriate for looking for effective protective strategies against Cd toxicity.

Thus, we have undertaken the study to investigate both the damaging impact of Cd on the kidney and the possibility of protection against it in a rat model (1 and 5 mg Cd/kg feed for 3–24 months) that well reflects the environmental exposure of the general population to this heavy metal in industrialized countries [17]. It has already been reported in the model that even low-level exposure (1 mg Cd/kg feed) injured the liver [18,19,20] and skeletal system [21,22,23], as well as destroyed the oxidative/reductive balance in the sublingual salivary glands [24] and the body status of zinc (Zn) [25], copper (Cu) [25], and manganese (Mn) [26]. In contrast, the co-administration of a 0.1% aqueous extract from the berries of *Aronia melanocarpa* L. (AM) had a protective impact [18,19,20,21,22,23,24,25,26]. The fruits of *A. melanocarpa* ((Michx.) Elliott, Rosaceae; chokeberry) are one of the richest sources of polyphenols in nature [27,28,29]. Chokeberries are also a rich source of vitamins (vitamins from group B and vitamins A, C, E, and K), macro- and microelements (e.g., calcium, magnesium, and iron), carotenoids (β-carotene), phytosterols, tannins, carbohydrates, triterpenes, pectins, sugar and sugar alcohols, organic acids, dietary fiber, and proteins [27,28,29]. Polyphenol-rich products are of particular interest among the possible agents that could be useful in counteracting the toxic action of Cd because of the multidirectional beneficial properties of these compounds, including antioxidative, anti-inflammatory, and anti-carcinogenic effects and their ability to bind the ions of divalent metals [27,29,30,31,32]. We have already revealed that the administration of AM during low-level (1 mg Cd/kg feed) and moderate (5 mg Cd/kg feed) intoxication with Cd significantly improved the antioxidative barrier (enzymatic and nonenzymatic), decreased concentrations of pro-oxidants, and protected from oxidative stress development and its consequences, such as oxidative modifications of lipids, proteins, and deoxyribonucleic acid (DNA) in the liver and changes in its morphological structure, as well as normalized the serum activities of liver enzyme markers [18,19]. Moreover, the intake of AM ameliorated Cd-mediated changes in the expression of collagen types I and III at the messenger ribonucleic acid (mRNA) and protein levels, as well as a rise in the concentrations of matrix metalloproteinases (MMP-1 and MMP-2) and their tissue inhibitors (TIMP-1 and TIMP-2) in the liver, indicating that it may be effective in preventing this heavy metal-caused disturbance in collagen homeostasis in this organ [20]. The intake of the extract at both levels of exposure to Cd provided important protection from this xenobiotic-induced oxidative stress, lipid peroxidation, and oxidative damage to the protein and DNA in the bone tissue, as well as from disturbances in bone turnover, and changes in bone mineral status [21,22]. Moreover, it improved bone collagen biosynthesis and femur biomechanical properties. The administration of the extract counteracted the development of oxidative stress and oxidative modifications of macromolecules not only in the liver [18,19] and bone tissue [22] but also in the sublingual salivary glands [24]. The supplementation with AM prevented or at least partially protected from most of the Cd-induced changes in the metabolism of Zn [25], Cu [25], and Mn [26], as well as ameliorated changes in the activity of Mn-dependent superoxide dismutase (Mn-SOD) and the concentration of Mn, and protected from Cd accumulation in the mitochondria, mainly in the liver [26].

The research so far carried out in the above-described experimental model of the general population’s exposure to Cd [17,18,19,20,21,22,23,24,25,26] allowed us to hypothesize that the exposure would also result in kidney damage. Taking into account the strong antioxidative potential of chokeberries [28,29,30] and prooxidative Cd effects [2,18,19,22,24], as well as the finding that the administration of AM during the treatment with this heavy metal decreased the body burden of this element, including its accumulation in the kidney [17], and counteracted numerous outcomes of the toxic action of this xenobiotic [18,19,20,21,22,23,24,25,26], it was hypothesized that the extract can also protect the kidney from damage. To investigate these hypotheses, we conducted a comprehensive study using a model of human environmental exposure to this heavy metal that we created. The present paper is the first report from this research, and it aimed to investigate whether low-level and moderate (1 and 5 mg Cd/kg feed, respectively) chronic exposure to Cd can result in damage to the function and structure of the kidney and if the administration of AM during intoxication can prevent these consequences. For this purpose, the sensitive biomarkers of damage to the kidney tubules (kidney injury molecule-1 (KIM-1), β2-microglobulin (β2-MG), N-acetyl-β-D-glucosaminidase (NAG), and alkaline phosphatase (ALP) in the urine) and clinically relevant early markers of glomerular injury (albumin concentration in the urine and creatinine clearance reflecting the glomerular filtration rate (GFR)), as well as other markers of the overall kidney function such as the concentrations of uric acid and urea in the serum and urine, and total protein concentration in the urine, were estimated. The morphological structure of the kidney and markers of inflammatory processes in this organ (chemerin, macrophage inflammatory protein 1 alpha (MIP1a), and Bcl2-associated X protein (Bax)) were evaluated as well. A similar study has not been conducted before. The present study is the first to not only assess Cd nephrotoxicity during intoxication, well reflecting the current levels of exposure of the general population worldwide, but also focus on the possibility of protection against this outcome via the administration of chokeberry extract.

## 2. Results

### 2.1. The Impact of Cd and AM Alone and Their Co-Administration on the Values of Biomarkers of Damage to the Kidney Tubules

**Figure 1:**
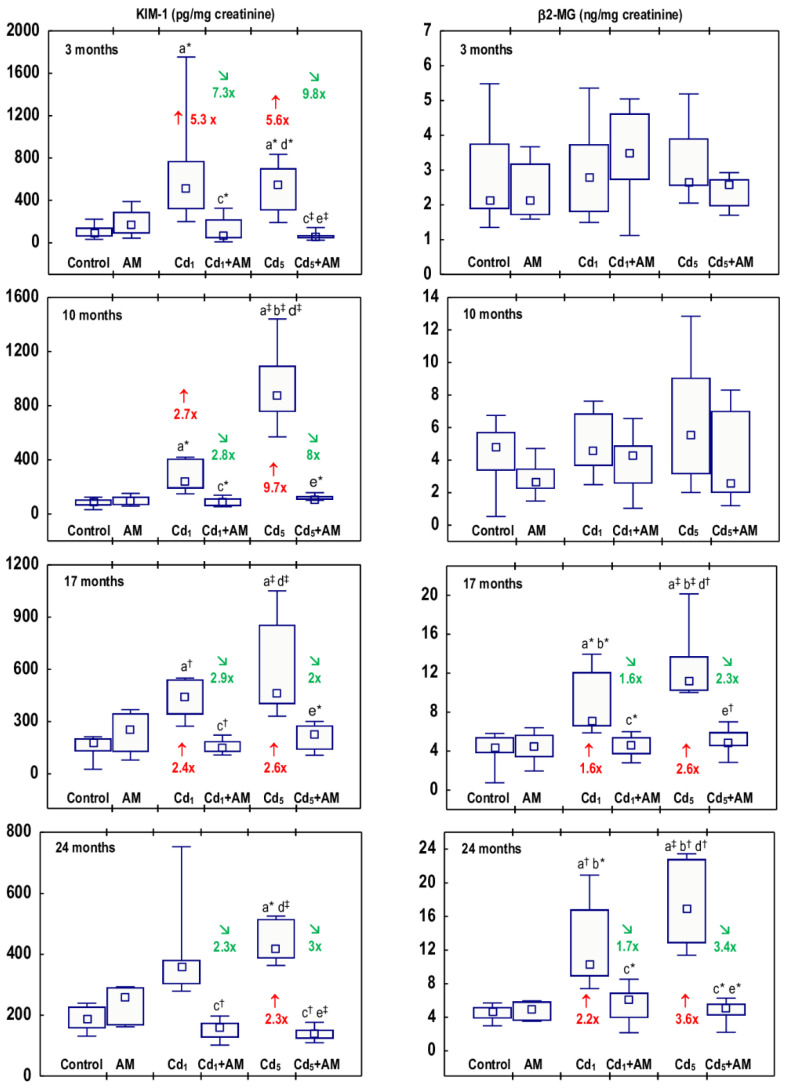
The concentrations of kidney injury molecule-1 (KIM-1) and β2-microglobulin (β2-MG) in the urine of female rats. The animals were treated with cadmium (Cd) in the feed at a concentration of 0, 1, or 5 mg/kg (Control, Cd_1_, and Cd_5_ groups) and/or 0.1% extract from the berries of *Aronia melanocarpa* L. (AM, Cd_1_+AM, and Cd_5_+AM groups) for 3, 10, 17, and 24 months. Data are shown as a median, 25–75% confidence interval, and minimum and maximum values for eight animals (except for seven females in the AM, Cd_1_, and Cd_5_ groups after 24 months). Statistically significant differences (Kruskal-Wallis test) compared to: a—Control group; b—AM group; c—Cd_1_ group; d—Cd_1_+AM group; and e—Cd_5_ group, where * *p* < 0.05, ^†^ *p* < 0.01, and ^‡^ *p* < 0.001 are marked. The factors of change compared to the control group (↑, increase) or the adequate group treated with Cd alone (↘, decrease) are indicated by the numerical values below or above the bars. Detailed data are presented in Table S1.

**Figure 2:**
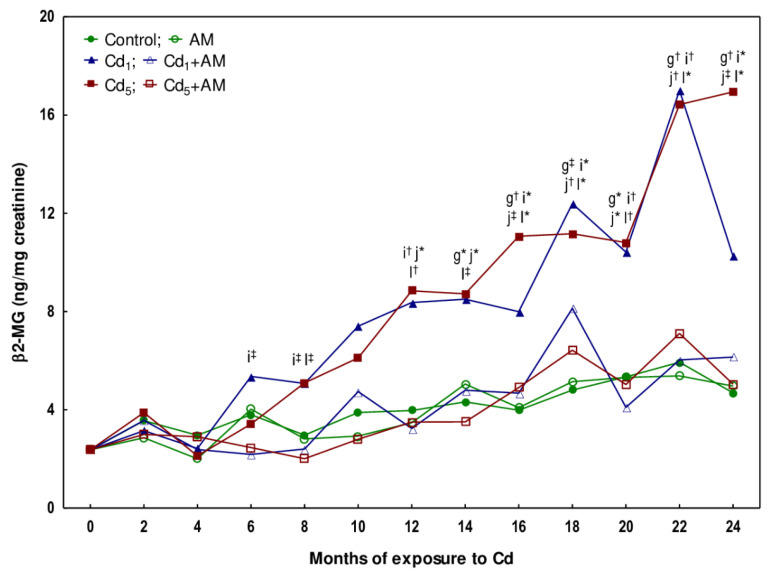
The concentration of β2-microglobulin (β2-MG) in the urine of female rats evaluated every other month during the 24-month study. The animals were treated with cadmium (Cd) in the feed at a concentration of 0, 1, or 5 mg/kg (Control, Cd_1_, and Cd_5_ groups) and/or 0.1% extract from the berries of *Aronia melanocarpa* L. (AM, Cd_1_+AM, and Cd_5_+AM groups). Data are shown as a median value for eight animals (except for seven females in the AM, Cd_1_, and Cd_5_ groups after 20, 22, and 24 months). An occurrence of statistically significant differences (Kruskal-Wallis test; * *p* < 0.05, ^†^ *p* < 0.01, and ^‡^ *p* < 0.001) between every two experimental groups at each time point was evaluated and marked as follows: f—AM and Control groups; g—Cd_1_ and Control groups; h—Cd_1_+AM and Control groups; i—Cd_1_ and Cd_1_+AM groups; j—Cd_5_ and Control groups; k—Cd_5_+AM and Control groups; l—Cd_5_ and Cd_5_+AM groups; m—Cd_1_ and Cd_5_ groups; and n—Cd_1_+AM and Cd_5_+AM groups. A lack of the particular letter symbol means a lack of statistically significant differences between appropriate groups. Detailed data are presented in Table S2.

**Figure 3:**
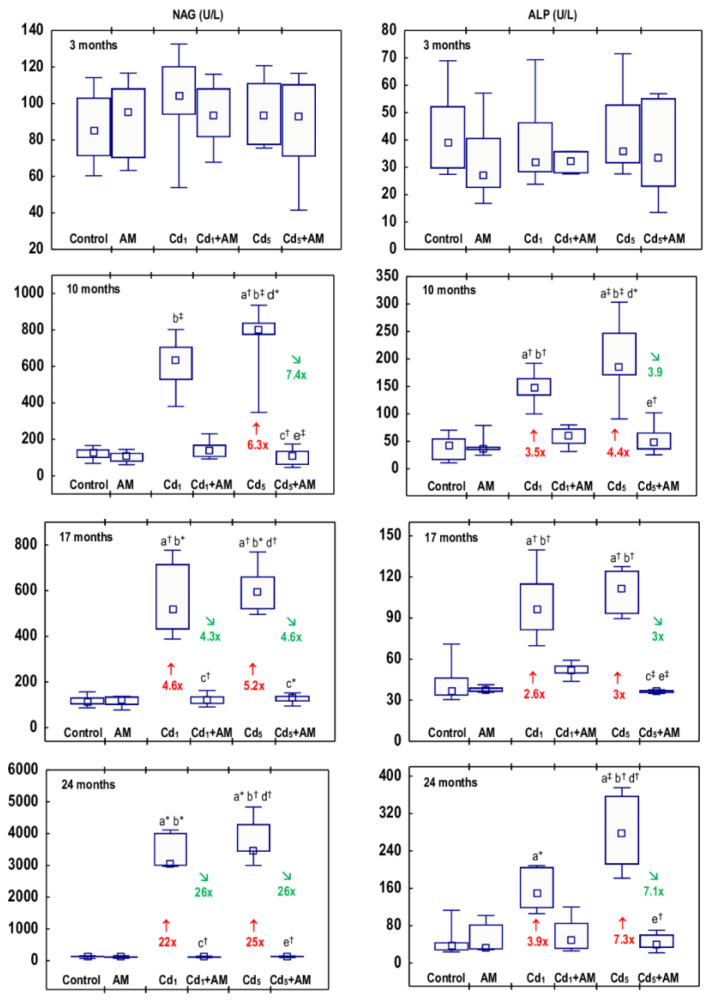
The activities of N-acetyl-β-D-glucosaminidase (NAG) and alkaline phosphatase (ALP) in the urine of female rats. The animals were treated with cadmium (Cd) in the feed at a concentration of 0, 1, or 5 mg/kg (Control, Cd_1_, and Cd_5_ groups) and/or 0.1% extract from the berries of *Aronia melanocarpa* L. (AM, Cd_1_+AM, and Cd_5_+AM groups) for 3, 10, 17, and 24 months. Data are shown as a median, 25–75% confidence interval, and minimum and maximum values for eight animals (except for seven females in the AM, Cd_1_, and Cd_5_ groups after 24 months). Statistically significant differences (Kruskal-Wallis test) compared to: a—Control group; b—AM group; c—Cd_1_ group; d—Cd_1_+AM group; and e—Cd_5_ group, where * *p* < 0.05, ^†^ *p* < 0.01, and ^‡^ *p* < 0.001 are marked. The factors of change compared to the control group (↑, increase) or the adequate group treated with Cd alone (↘, decrease) are indicated by the numerical values below or above the bars. Detailed data are presented in Table S3.

**Figure 4:**
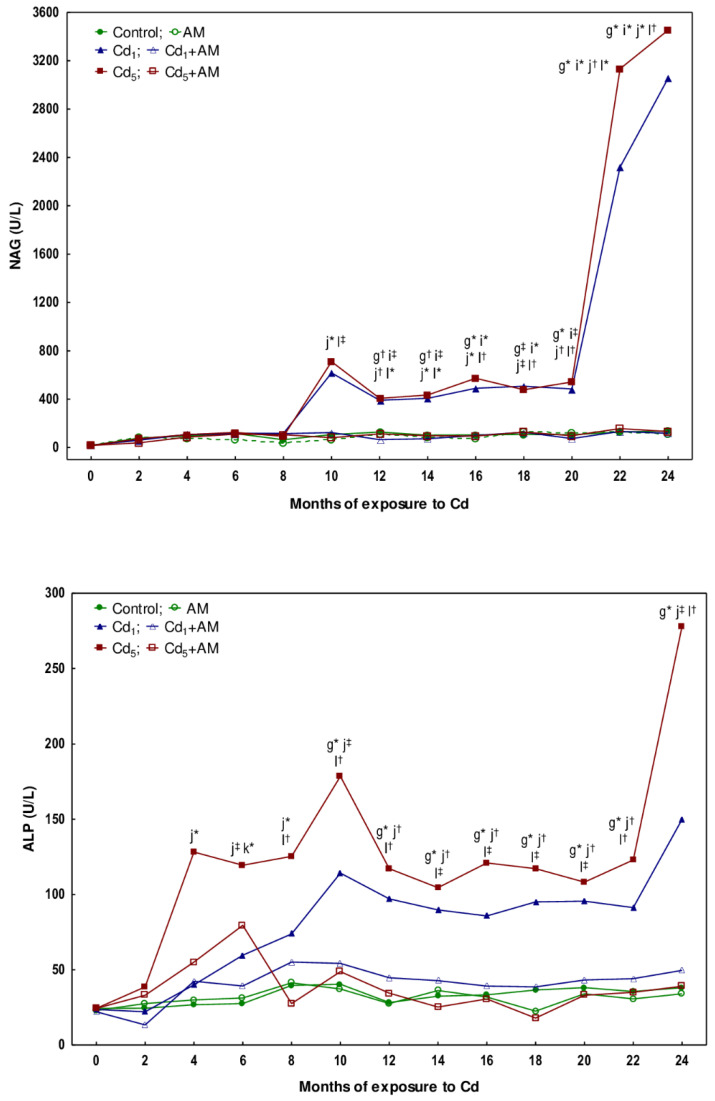
The activities of N-acetyl-β-D-glucosaminidase (NAG) and alkaline phosphatase (ALP) in the urine of female rats evaluated every other month during the 24-month study. The animals were treated with cadmium (Cd) in the feed at a concentration of 0, 1, or 5 mg/kg (Control, Cd_1_, and Cd_5_ groups) and/or 0.1% extract from the berries of *Aronia melanocarpa* L. (AM, Cd_1_+AM, and Cd_5_+AM groups). Data are shown as a median value for eight animals (except for seven females in the AM, Cd_1_, and Cd_5_ groups after 20, 22, and 24 months). An occurrence of statistically significant differences (Kruskal-Wallis test; * *p* < 0.05, ^†^ *p* < 0.01, and ^‡^ *p* < 0.001) between every two experimental groups at each time point was evaluated and marked as follows: f—AM and Control groups; g—Cd_1_ and Control groups; h—Cd_1_+AM and Control groups; i—Cd_1_ and Cd_1_+AM groups; j—Cd_5_ and Control groups; k—Cd_5_+AM and Control groups; l—Cd_5_ and Cd_5_+AM groups; m—Cd_1_ and Cd_5_ groups; and n—Cd_1_+AM and Cd_5_+AM groups. A lack of the particular letter symbol means a lack of statistically significant differences between appropriate groups. Detailed data are presented in Tables S4 (NAG) and S5 (ALP).

The administration of AM alone for up to 24 months had no effect on any of the biomarkers of damage to the kidney tubules (KIM-1, β2-MG, NAG, and ALP) determined in the urine (Figure 1, Figure 2, Figure 3 and Figure 4, Tables S1–S5). The exposure to Cd in fodder at concentrations of 1 and 5 mg/kg affected the levels of all biomarkers of tubular damage, and the occurrence of these effects was dependent on the level and duration of exposure; however, at any of the time points, there were no differences in the values of specific parameters between the Cd_1_ and Cd_5_ groups (Figure 1, Figure 2, Figure 3 and Figure 4, Tables S1–S5).

In the rats maintained on the feed containing 1 and 5 mg Cd/kg for 3, 10, 17, and 24 months, the concentration of KIM-1 in the urine was higher (2.3–9.7 times) compared to the control animals, except for the Cd_1_ group after 24 months. Although the Kruskal-Wallis post hoc test showed a lack of difference (*p* > 0.05) in KIM-1 concentration between the Cd_1_ group (range 279.0–752.6 pg/mg creatinine) and the control group (range 131.2–239.3 pg/mg creatinine), this parameter in particular animals in the group exposed to Cd reached higher numerical values than in the control group (Figure 1, Table S1).

The concentration of β2-MG in the urine was unchanged after the 3- and 10-month feeding with the 1 and 5 mg Cd/kg diet compared to the control group; however, after 17 and 24 months, it was increased (1.6–3.6-fold) (Figure 1, Table S1). The measurements of β2-MG concentration, carried out every other month in the animals maintained in the experiment for 24 months, revealed that at the exposure to 1 mg Cd/kg feed, the concentration of this low-molecular-weight protein increased compared to the control group after 14 months (2-fold), while in the case of the higher treatment, it was elevated (2.2-fold) after 12 months, and at both levels of exposure, it remained at the increased level (2–2.9 times in the Cd_1_ group and 2–3.6 times in the Cd_5_ group, compared to the control group) until the end of the study (Figure 2, Table S2).

The activity of NAG in the urine of rats fed with fodder containing 1 mg Cd/kg for 3 and 10 months was within the range of values noted in the control group, while after 17 and 24 months, it was increased (4.6- and 22-fold, respectively) (Figure 3, Table S3). In the animals exposed to the 5 mg Cd/kg feed for 10, 17, and 24 months, this enzyme activity was elevated 5.2–25-fold (Figure 3, Table S3). The measurements of NAG taken every two months during the 24-month study disclosed that at low-level exposure to Cd, the urinary activity of this enzyme began to increase after 12 months (was 3.1-fold higher than in the control group) and confirmed the rise in this enzyme activity after 10 months of the higher exposure noted in the animals sacrificed at this time point (Figure 3 and Figure 4). At both levels of exposure to Cd, the activity of NAG, once increased, remained at an elevated (3.2–5.5-fold compared to the control group) but relatively stable level during the following months; however, between the 20th and 22nd months, a marked increase (4.8- and 5.8-fold in the Cd_1_ and Cd_5_ groups, respectively) in this enzyme activity (up to values 18 and 25 times higher in the Cd_1_ and Cd_5_ groups, respectively, than in the control group) was noted (Figure 4, Table S4).

In the animals maintained on the feed containing 1 and 5 mg Cd/kg for 10, 17, and 24 months, the activity of ALP in the urine was increased (2.6–7.3-fold) (Figure 3; Table S3). Bimonthly monitoring of ALP activity in the animals maintained in the experiment for 24 months revealed that at moderate exposure to Cd, the activity of this enzyme increased as early as after 4 months (4.8-fold), whereas in the low-level treatment, it rose (2.8-fold) after 10 months, analogously as in the animals sectioned after this time of exposure duration (Figure 4, Table S5). Once the activity of ALP increased, it remained elevated (2.5–3.9 times in the Cd_1_ group and 3.2–7.3 times in the Cd_5_ group) until the end of the experiment (Figure 4, Table S5). In the Cd_5_ group, between the 22nd and 24th months, a marked increase (2.3-fold) in ALP activity was noted (Figure 4, Table S5).

The administration of AM during the exposure to the 1 and 5 mg Cd/kg feeds entirely prevented all the changes described above in the values of KIM-1, β2-MG, NAG, and ALP except for the rise in ALP activity after 6 months of the treatment with the 5 mg Cd/kg feed (Figure 1, Figure 2, Figure 3 and Figure 4, Tables S1–S5). The activity of ALP in the Cd_5_+AM group after 6 months was 2.9-fold higher compared to the control group and did not differ compared to the Cd_5_ group (Figure 4, Table S5). Apart from this one exception, the values of all biomarkers of damage to the kidney tubules in the Cd_1_+AM group and the Cd_5_+AM group were within the ranges of values determined in the control group (Figure 1, Figure 2, Figure 3 and Figure 4, Tables S1–S5).

### 2.2. The Impact of Cd and AM Alone and Their Co-Administration on the Values of Biomarkers of Damage to the Kidney Glomeruli

**Figure 5:**
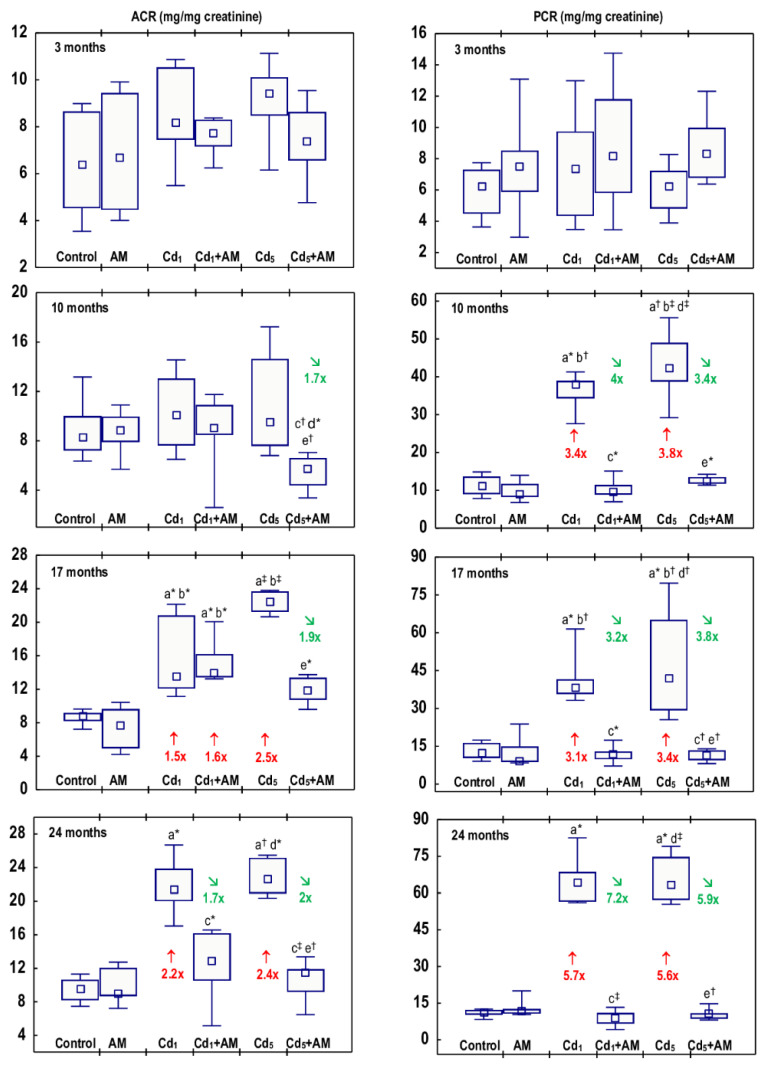
The concentrations of albumin and total protein in the urine of female rats adjusted for creatinine concentration (ACR and PCR, respectively). The animals were treated with cadmium (Cd) in the feed at a concentration of 0, 1, or 5 mg/kg (Control, Cd_1_, and Cd_5_ groups) and/or 0.1% extract from the berries of *Aronia melanocarpa* L. (AM, Cd_1_+AM, and Cd_5_+AM groups) for 3, 10, 17, and 24 months. Data are shown as a median, 25–75% confidence interval, and minimum and maximum values for eight animals (except for seven females in the AM, Cd_1_, and Cd_5_ groups after 24 months). Statistically significant differences (Kruskal-Wallis test) compared to: a—Control group; b—AM group; c—Cd_1_ group; d—Cd_1_+AM group; and e—Cd_5_ group, where * *p* < 0.05, ^†^ *p* < 0.01, and ^‡^ *p* < 0.001 are marked. The factors of change compared to the control group (↑, increase) or the adequate group treated with Cd alone (↘, decrease) are indicated by the numerical values below or above the bars. Detailed data are presented in Table S6.

**Figure 6:**
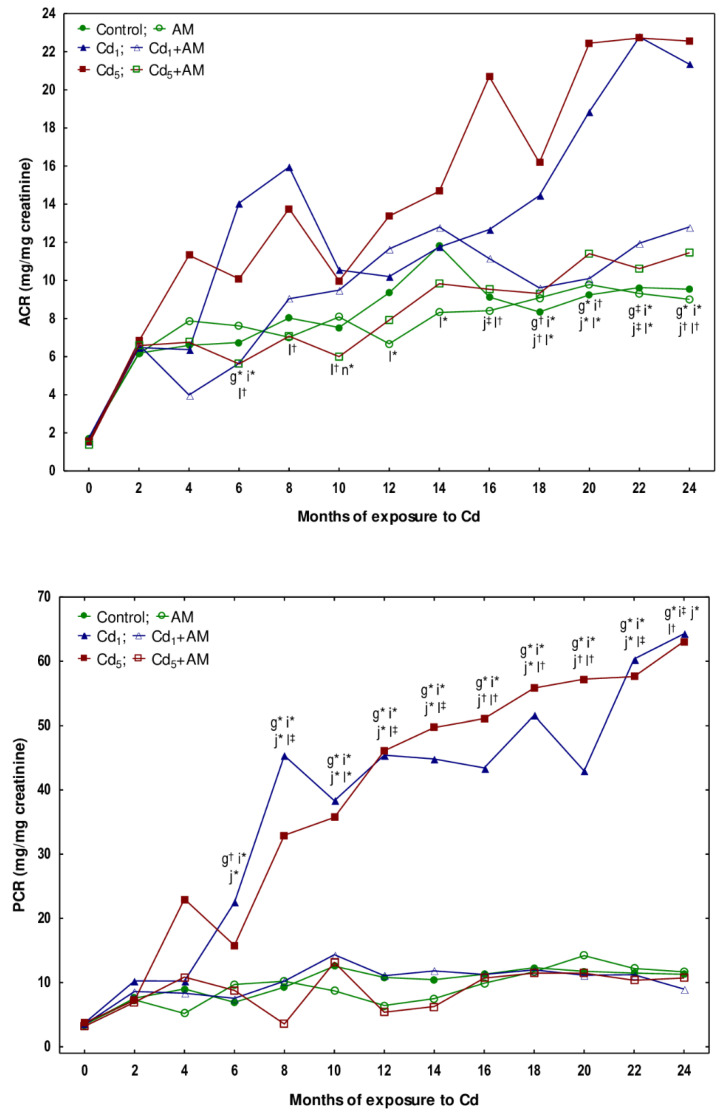
The concentrations of albumin and total protein in the urine of female rats adjusted for creatinine concentration (ACR and PCR, respectively) evaluated every other month during the 24-month study. The animals were treated with cadmium (Cd) in the feed at s concentration of 0, 1, or 5 mg/kg (Control, Cd_1_, and Cd_5_ groups) and/or 0.1% extract from the berries of *Aronia melanocarpa* L. (AM, Cd_1_+AM, and Cd_5_+AM groups). Data are shown as a median values for eight animals (except for seven females in the AM, Cd_1_, and Cd_5_ groups after 20, 22, and 24 months). An occurrence of statistically significant differences (Kruskal-Wallis test; * *p* < 0.05, ^†^ *p* < 0.01, and ^‡^ *p* < 0.001) between every two experimental groups at each time point was evaluated and marked as follows: f—AM and Control groups; g—Cd_1_ and Control groups; h—Cd_1_+AM and Control groups; i—Cd_1_ and Cd_1_+AM groups; j—Cd_5_ and Control groups; k—Cd_5_+AM and Control groups; l—Cd_5_ and Cd_5_+AM groups; m—Cd_1_ and Cd_5_ groups; and n—Cd_1_+AM and Cd_5_+AM groups. A lack of the particular letter symbol means a lack of statistically significant differences between appropriate groups. Detailed data are presented in Tables S7 (ACR) and S8 (PCR).

**Figure 7:**
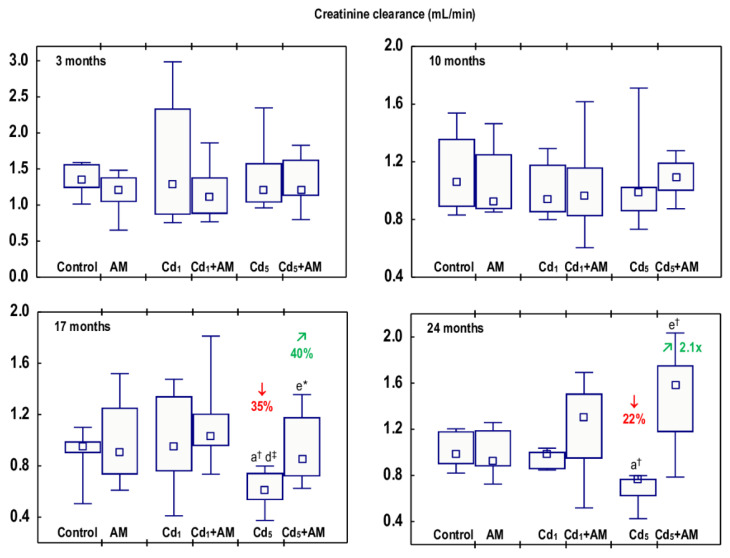
Creatinine clearance in female rats. The animals were treated with cadmium (Cd) in the feed at a concentration of 0, 1, or 5 mg/kg (Control, Cd_1_, and Cd_5_ groups) and/or 0.1% extract from the berries of *Aronia melanocarpa* L. (AM, Cd_1_+AM, and Cd_5_+AM groups) for 3, 10, 17, and 24 months. Data are shown as a median, 25–75% confidence interval, and minimum and maximum values for eight animals (except for seven females in the AM, Cd_1_, and Cd_5_ groups after 24 months). Statistically significant differences (Kruskal-Wallis test) compared to: a—Control group; d—Cd_1_+AM group; and e—Cd_5_ group, where * *p* < 0.05, ^†^ *p* < 0.01, and ^‡^ *p* < 0.001 are marked. The percentage changes or a factor of change compared to the control group (**↓**, decrease) or the adequate group treated with Cd alone (**↗**, increase) are indicated by the numerical values above the bars. Detailed data are presented in Table S9.

**Figure 8:**
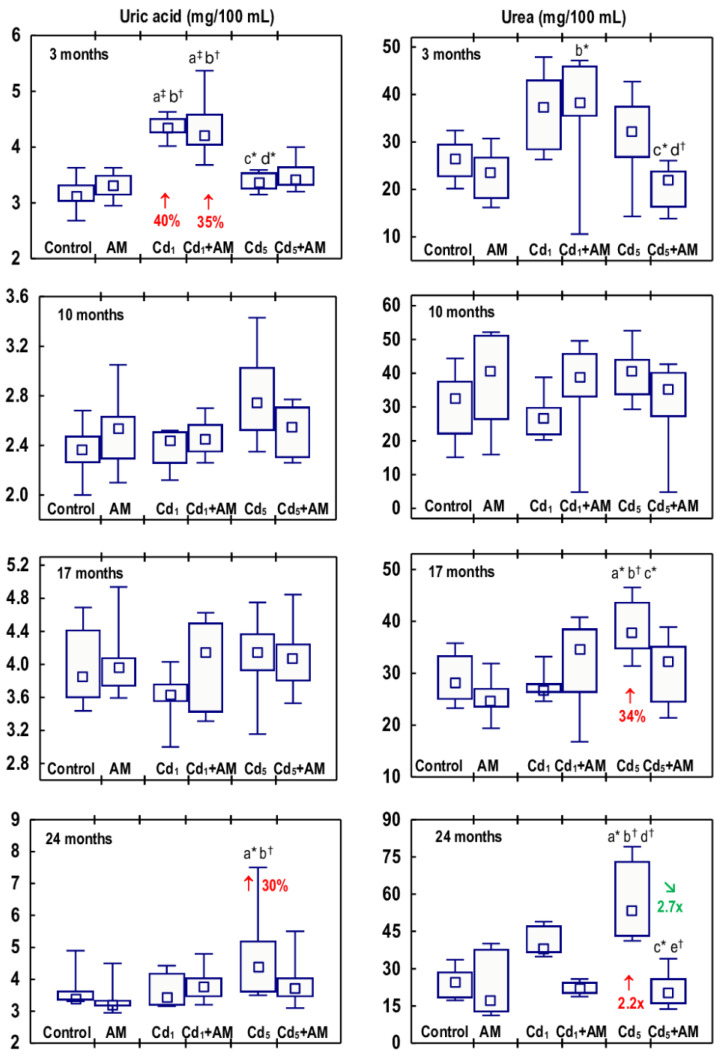
The concentrations of uric acid and urea in the serum of female rats. The animals were treated with cadmium (Cd) in the feed at a concentration of 0, 1, or 5 mg/kg (Control, Cd_1_, and Cd_5_ groups) and/or 0.1% extract from the berries of *Aronia melanocarpa* L. (AM, Cd_1_+AM, and Cd_5_+AM groups) for 3, 10, 17, and 24 months. Data are shown as a median, 25–75% confidence interval, and minimum and maximum values for eight animals (except for seven females in the AM, Cd_1_, and Cd_5_ groups after 24 months). Statistically significant differences (Kruskal-Wallis test) compared to: a—Control group; b—AM group; c—Cd_1_ group; d—Cd_1_+AM group; and e—Cd_5_ group—where * *p* < 0.05, ^†^ *p* < 0.01, and ^‡^ *p* < 0.001 are marked. The percentage changes or factors of changes compared to the control group (↑, increase) or the adequate group treated with Cd alone (↘, decrease) are indicated by the numerical values below or above the bars. Detailed data are presented in Table S10.

In the rats administered AM alone for up to 24 months, the concentrations of albumin and total protein in the urine adjusted for creatinine concentration (ACR and PCR, respectively), creatinine concentration in the serum and urine, creatinine clearance, as well as the concentrations of uric acid and urea in the serum and their content in the 24-h urine, were within the ranges of the control group (Figure 5, Figure 6, Figure 7 and Figure 8, Tables S6–S11).

Low-level and moderate exposure to Cd for 17 and 24 months resulted in an increase in ACR (1.5–2.5-fold) (Figure 5, Table S6). The estimation of ACR every other month in the animals maintained in the experiment for 24 months revealed that in the Cd_1_ group, a temporary growth (2.1-fold) in this parameter was noted after 6 months. Next, for several months, ACR remained proper, while after 18 months, it increased again and was enhanced (1.7–2.4-fold) until the end of the experiment (Figure 6, Table S7). In the females treated with the 5 mg Cd/kg feed, ACR increased (2.3-fold) after 16 months and remained at the elevated (1.9–2.4 times) level until the end of the study (Figure 6, Table S7).

In the animals sectioned after 10, 17, and 24 months, PCR was higher (3.1–5.7 times) than in the control group (Figure 5, Table S6). The measurements carried out every other month at both levels of exposure to Cd disclosed that PCR increased for the first time after 6 months (3.2 and 2.3 times in the Cd_1_ and Cd_5_ groups, respectively) and remained enhanced (3.0–5.7 times and 2.8–5.6 times in the Cd_1_ and Cd_5_ groups, respectively) until the end of the 24-month study (Figure 6, Table S8).

The exposure to Cd at the concentrations of 1 and 5 mg Cd/kg feed had no impact on creatinine concentrations in the serum or urine (data presented only in the Supplementary Materials; Table S9). The creatinine clearance was not affected by the low-level exposure to Cd for 3–24 months, but it decreased after 17 and 24 months of the moderate treatment (by 35% and 22%, respectively) (Figure 7, Table S9).

The concentrations of uric acid and urea in the serum in the Cd_1_ group were unaffected, except for an increase (by 40%) in uric acid concentration after 3 months (Figure 8, Table S10). In the Cd_5_ group, urea concentration was enhanced after 17 and 24 months (by 34% and 2.2-fold, respectively), and uric acid concentration was elevated (by 30%) after 24 months (Figure 8, Table S10). Intoxication with Cd in fodder at concentrations of 1 and 5 mg/kg had no impact on the content of uric acid and urea in the 24-h urine (data presented only in the Supplementary Materials; Table S11).

There were no differences in the values of particular markers of glomerular function between the Cd_1_ group and the Cd_5_ group, except for a lower (by 22%) concentration of uric acid in the serum after 3 months and a higher (by 41%) concentration of urea after 17 months in the Cd_5_ group (Figure 5, Figure 6, Figure 7 and Figure 8, Tables S6–S11).

All of the evaluated biomarkers of damage to the kidney glomeruli (ACR, PCR, creatinine concentration in the serum and urine, and creatinine clearance, as well as the concentrations of uric acid and urea in the serum and their content in the 24-h urine) in the animals co-administered with Cd and AM were within the ranges of values determined in the control group, except for ACR in the Cd_1_+AM group after 17 months and the serum concentration of uric acid after 3 months that were increased (1.6-fold and by 35%, respectively) compared to the control group but did not differ versus the Cd_1_ group (Figure 5, Figure 6, Figure 7 and Figure 8, Tables S6–S11). Moreover, creatinine concentration in the serum in the Cd_5_+AM group after 3, 17, and 24 months was lower (by 26–39%) than in the Cd_5_ group (Table S9).

### 2.3. The Impact of Cd and AM Alone and Their Co-Administration on the Concentration of Cd in the Urine

**Figure 9:**
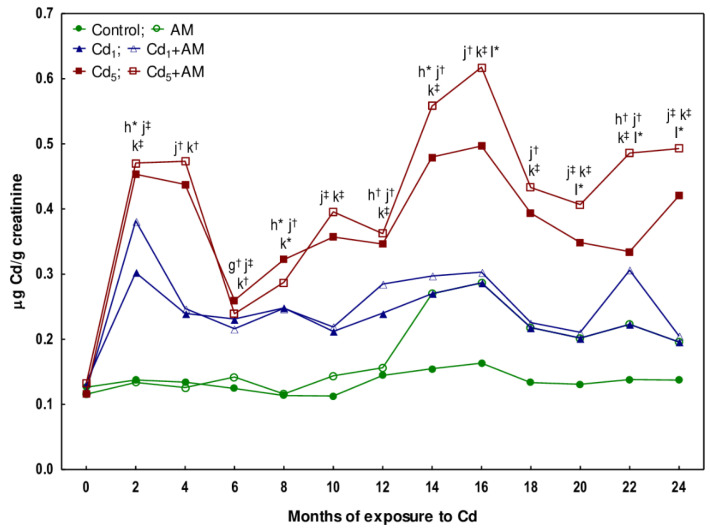
The concentration of cadmium (Cd) in the urine of female rats evaluated every other month during the 24-month study. The animals were treated with Cd in the feed at a concentration of 0, 1, or 5 mg/kg (Control, Cd_1_, and Cd_5_ groups) and/or 0.1% extract from the berries of *Aronia melanocarpa* L. (AM, Cd_1_+AM, and Cd_5_+AM groups). Data are shown as a median value for eight animals (except for seven females in the AM, Cd_1_, and Cd_5_ groups after 20, 22, and 24 months). An occurrence of statistically significant differences (Kruskal-Wallis test; * *p* < 0.05, ^†^ *p* < 0.01, and ^‡^ *p* < 0.001) between every two experimental groups at each time point was evaluated and marked as follows: f—AM and Control groups; g—Cd_1_ and Control groups; h—Cd_1_+AM and Control groups; i—Cd_1_ and Cd_1_+AM groups; j—Cd_5_ and Control groups; k—Cd_5_+AM and Control groups; l—Cd_5_ and Cd_5_+AM groups; m—Cd_1_ and Cd_5_ groups; and n—Cd_1_+AM and Cd_5_+AM groups. A lack of the particular letter symbol means a lack of statistically significant differences between appropriate groups. Detailed data are presented in Table S12.

The concentration of Cd in the urine of the female rats maintained on the feed containing 1 mg Cd/kg, evaluated every other month throughout the 24-month study, did not differ compared to the control group, except for its higher (by 86%) value after 6 months, and ranged from 0.1114 to 0.6386 μg Cd/g creatinine (Figure 9, Table S12). In the animals fed with the 5 mg Cd/kg diet, the concentration of this toxic element in the urine starting from the 2nd month until the end of the experiment ranged from 0.1664 to 0.9785 μg Cd/g creatinine and was higher (2.1–3.3-fold) than in the control group (0.0620–0.2894 μg Cd/g creatinine) but did not differ statistically significantly compared to the Cd_1_ group; however, the median concentration of this heavy metal reached clearly higher numerical values (Figure 9, Table S14).

The administration of AM to the animals maintained on the standard diet (containing 0.0584 ± 0.0049 mg/kg) and the 1 mg Cd/kg diet had no impact on Cd concentration in 24-h urine samples (Figure 9, Table S14). In the Cd_5_+AM group, the Cd concentration in the urine after 16 months and between the 20th and 24th months was higher by 17–45% compared to the Cd_5_ group (Figure 9, Table S14).

### 2.4. The Impact of Cd and AM Alone and Their Co-Administration on the Morphological Structure of the Kidney

The macroscopic picture of both kidneys in the control group was normal. The kidney was a bean-shaped organ of brick-red color and had a soft consistency. In the animals that received Cd and AM alone and together, the macroscopic picture of the kidneys did not differ from that in the control group.

Both kidneys in each rat had the same weight. The median absolute weight of the left kidney of control females reached 0.9420 g (0.8595–1.0100 g) after 3 months and 1.3065 g (1.1163–2.0292 g) after 24 months, whereas the relative weight of this organ was 0.3032 g/100 g body weight (b.w.) (0.2732–0.3222 g/100 g b.w.) and 0.1937 g/100 g b.w. (0.1786–0.3624 g/100 g b.w.), respectively (Table S13). There were no differences in the absolute and relative weight of the kidney between the experimental groups throughout the study (Table S13).

**Figure 10:**
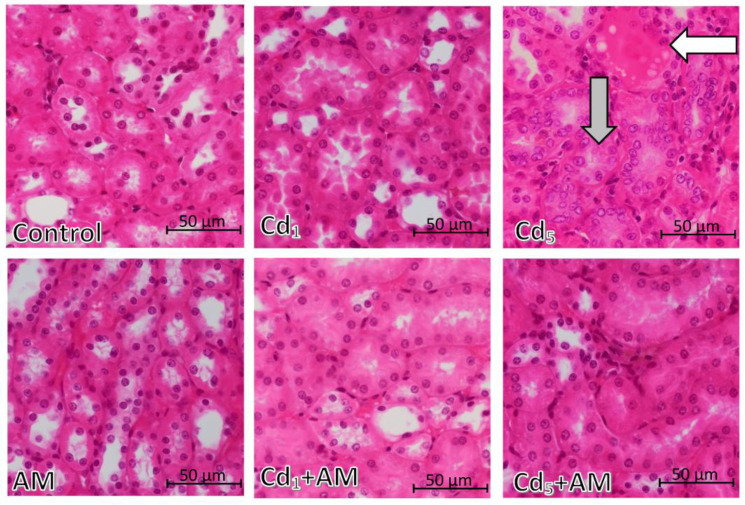
Histopathological image of the renal tubules of female rats in hematoxylin–eosin (H+E) staining. The animals were treated with cadmium (Cd) in the feed at a concentration of 0, 1, or 5 mg/kg (Control, Cd_1_, and Cd_5_ groups) and/or 0.1% extract from the berries of *Aronia melanocarpa* L. (AM, Cd_1_+AM, and Cd_5_+AM groups) for 24 months. Tubular changes were the most advanced in the Cd_5_ group, in which hyalinization (white arrow), hyperplasia, and hypertrophy (grey arrow) of the tubular epithelium and pronounced proliferation of the interstitial tissue of the kidney were found.

**Figure 11:**
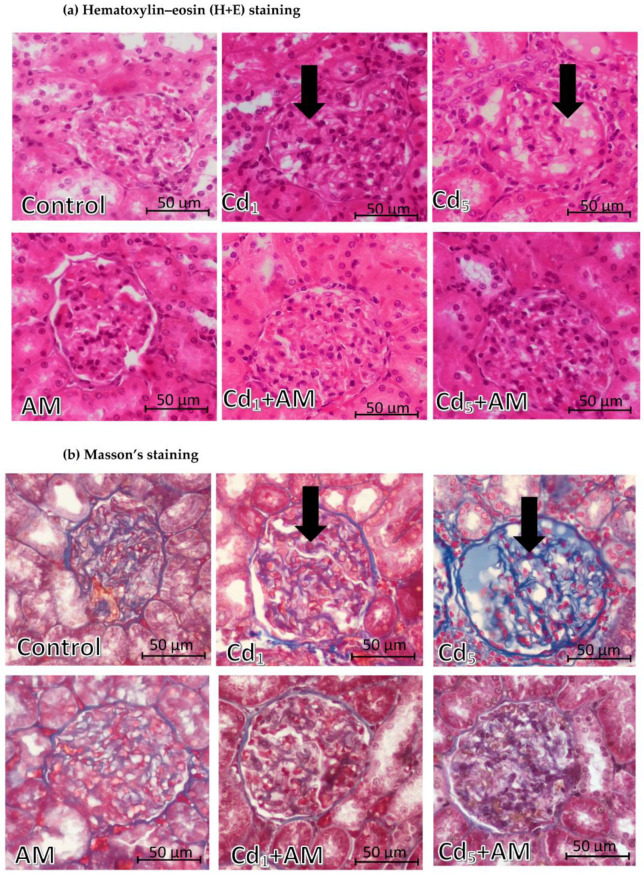
Histopathological image of the renal glomeruli of female rats. The animals were treated with cadmium (Cd) in the feed at a concentration of 0, 1, or 5 mg/kg (Control, Cd_1_, and Cd_5_ groups) and/or 0.1% extract from the berries of *Aronia melanocarpa* L. (AM, Cd_1_+AM, and Cd_5_+AM groups) for 24 months. In the Cd_1_ and Cd_5_ groups, moderate and severe, respectively, glomerulonephritis is evident. There is a thickening of the basement membranes (blue in Masson’s staining) of the glomerulus and progressive atrophy of the mesangium (decrease in cellularity) (black arrow). In the Cd_1_+AM and Cd_5_+AM groups, minimal or slight, respectively, glomerulonephritis is observed.

The morphological microscopic image of the kidney in the control group was almost proper, except for slight glomerulonephritis (found in 50% of the animals) and perivascular oedema (occurring in 25% of the animals) (Table 1, Figure 10 and Figure 11). In the AM group, slight intensity changes such as tubular vacuolization, an extension of the tubular lumen, hyperplasia of the epithelium of the convoluted tubules, or glomerulonephritis were observed, and each of these changes occurred only in one female (25%) (Table 1, Figure 10 and Figure 11).

In the animals maintained for 24 months on the feed containing 1 and 5 mg Cd/kg (Cd_1_ and Cd_5_ groups), pathological changes in the tubules such as vacuolization (only in the Cd_1_ group), hyalinization, an extension of the tubular lumen (only in the Cd_1_ group), hyperplasia and hypertrophy of the epithelium of the convoluted tubules, and proliferation of the interstitial tissue of the kidney, as well as glomerulonephritis and congestion at the cortex/medullary interface and perivascular oedema (only in the Cd_5_ group), were observed (Table 1, Figure 10 and Figure 11). There was no tubular necrosis or glomerular congestion (Table 1). The Cd-induced changes in the morphological structure of the kidney in the Cd_5_ group were more advanced compared to those noted in the Cd_1_ group, except for tubular vacuolization, which was not observed in the Cd_5_ group (Table 1, Figure 10 and Figure 11).

In the animals administered with AM during the treatment with Cd (Cd_1_+AM and Cd_5_+AM groups), the intensity of pathological changes in the morphological structure of the kidney was milder compared to the respective groups that did not receive the extract under the exposure to Cd (Cd_1_ and Cd_5_ groups) (Table 1, Figure 10 and Figure 11). The administration of AM at the exposure to the 1 mg Cd/kg feed completely protected the tubular vacuolization and hyalinization, an extension of the tubular lumen, and hyperplasia and hypertrophy of the epithelium of the convoluted tubules, as well as a weakened proliferation of interstitial tissue of the kidney (Table 1, Figure 10). The application of the extract at the time of intoxication with 5 mg Cd/kg feed prevented hyperplasia of the epithelium of the convoluted tubules and attenuated the other Cd-induced changes in the histological structure of tubules, such as hyalinization, hypertrophy of the epithelium of the convoluted tubules, and interstitial proliferation (Table 1, Figure 10). However, a slight extension of the tubular lumen was observed in 50% of animals in the Cd_5_+AM group, whereas no such change occurred in the Cd_5_ group (Table 1). In the Cd_1_ group, glomerulonephritis developed in all animals, and in 25% of the females, it was slight and in 75%, moderate, while in the Cd_1_+AM group, it was noted only in 25% of the animals and was slight. Similarly, in the Cd_5_ group, glomerulonephritis was severe in 75% of the animals and moderate in 25% of the females, whereas in the Cd_5_+AM group, it was noted only in 50% of the animals and was slight in intensity (Table 1, Figure 11). The administration of AM during the exposure to 1 and 5 mg Cd/kg feed also weakened congestion at the cortex/medullary interface. In addition, AM administration under higher exposure to Cd resulted in a weakening of perivascular oedema; however, in the Cd_1_+AM group, a slight intensity change of this kind was noted in 25% of animals, while in the Cd_1_ group, it was absent (Table 1).

### 2.5. The Impact of Cd and AM Alone and Their Co-Administration on the Markers of Inflammation in the Kidney

**Figure 12:**
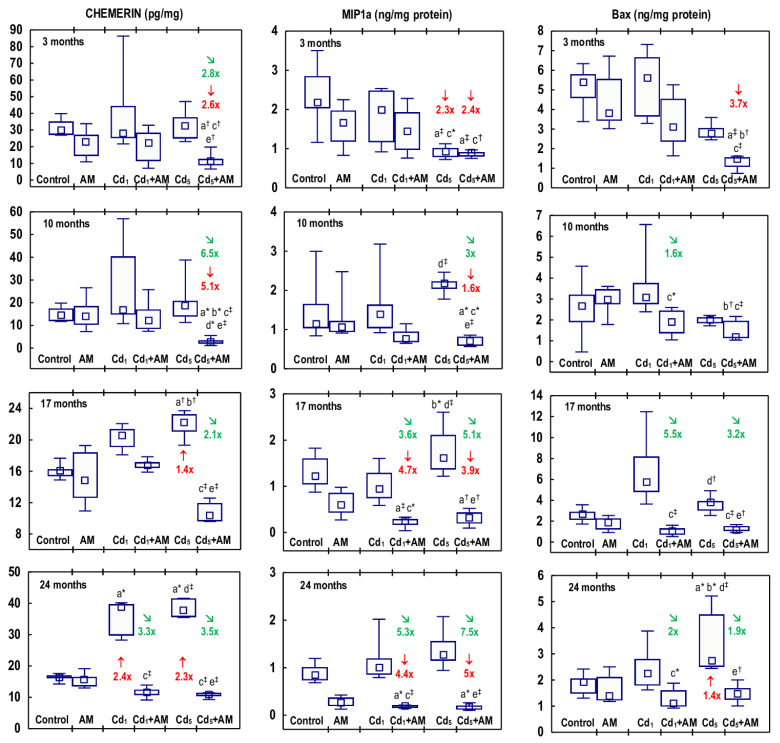
The concentrations of chemerin, macrophage inflammatory protein 1 alpha (MIP1a), and Bcl2-associated X protein (Bax) in the kidneys of female rats. The animals were treated with cadmium (Cd) in the diet at a concentration of 0, 1, or 5 mg/kg (Control, Cd_1_, and Cd_5_ groups) and/or 0.1% extract from the berries of *Aronia melanocarpa* L. (AM, Cd_1_+AM, and Cd_5_+AM groups) for 3, 10, 17, and 24 months. Data are shown as a median, 25–75% confidence interval, and minimum and maximum values for eight animals (except for seven females in the AM, Cd_1_, and Cd_5_ groups after 24 months). Statistically significant differences (Kruskal-Wallis test) compared to: a—Control group; b—AM group; c—Cd_1_ group; d—Cd_1_+AM group; and e—Cd_5_ group—where * *p* < 0.05, ^†^ *p* < 0.01, and ^‡^ *p* < 0.001 are marked. The factors of change compared to the control group (↓, decrease; ↑, increase) or the adequate group treated with Cd alone (↘, decrease) are indicated by the numerical values below or above the bars. Detailed data are presented in Table S14.

The administration of AM alone for up to 24 months had no impact on the concentrations of chemerin, MIP1a, and Bax in the kidney (Figure 12, Table S14).

In the rats treated with the 1 mg Cd/kg feed, the concentration of chemerin in the kidney tissue was increased (2.4-fold) after 24 months, whereas the concentrations of MIP1a and Bax throughout the whole experiment were unchanged compared to the control group (Figure 12, Table S14). In the animals fed with fodder containing 5 mg Cd/kg, chemerin concentration was elevated after 17 and 24 months (1.4- and 2.3-fold, respectively), and Bax concentration was enhanced after 24 months (1.4-fold), whereas the concentration of MIP1a was decreased (2.3-fold) after 3 months (Figure 12, Table S14). The kidney concentrations of chemerin, MIP1a, and Bax did not differ between the Cd_1_ and Cd_5_ groups, except for the lower (by 53%) concentration of MIP1a in the Cd_5_ group after 3 months (Figure 12, Table S14).

The administration of AM during the exposure to Cd prevented these heavy-metal-induced changes in the concentration of chemerin, but it decreased the exposure to the 5 mg Cd/kg feed-unchanged concentration of this parameter after 3 and 10 months compared to the control and Cd_5_ groups (2.6–6.5-fold) (Figure 12, Table S14). The concentration of MIP1a in the Cd_1_+AM group after 17 and 24 months and in the Cd_5_+AM group at all time points was lower compared to the control group, except for a lack of difference in the concentration of MIP1a between the Cd_5_ group and the Cd_5_+AM group after 3 months (Figure 12, Table S14). In the animals administered with AM during the 10-, 17-, and 24-month feeding with fodder containing 1 mg Cd/kg, the concentration of Bax was lower (1.6–5.5-fold) compared to the Cd_1_ group; however, it did not differ compared to the control group (Figure 12, Table S14). The 3-month administration of AM to the females maintained on the 5 mg Cd/kg feed resulted in a decrease (3.7-fold) in the concentration of Bax compared to the control group. After 10, 17, and 24 months, Bax concentration in the Cd_5_+AM group did not differ compared to the control, and after 17 and 24 months, it was lower (3.2 and 1.9 times, respectively) than in the Cd_5_ group (Figure 12, Table S14).

### 2.6. Relationships between the Investigated Biomarkers of Kidney Status and the Body Burden of Cd

In the female rats that were not administered with AM (the control group and the Cd_1_ and Cd_5_ groups), positive dependencies were noted between the indices of the body burden of Cd, such as this heavy metal concentration in the blood, urine, and kidney, and all evaluated markers of renal tubular damage (KIM-1, β2-MG, NAG, and ALP), as well as indices of glomerular damage such as ACR, PCR, and the serum concentrations of uric acid and urea, except for a lack of dependence between NAG activity and Cd concentration in the urine (Table 2). The creatinine clearance was negatively correlated with the Cd concentration in the blood and kidney (Table 2). Moreover, the kidney concentration of chemerin was positively correlated with the Cd concentration in the blood (Table 2).

In the rats that were administered AM alone and during the exposure to Cd (the AM, Cd_1_+AM, and Cd_5_+AM groups), there were no dependencies between the Cd concentration in the blood, urine, and kidney and the indices of tubular and glomerular damage, except for positive correlations between the kidney Cd concentration and β2-MG, NAG, and ACR (Table 2). Moreover, negative dependencies occurred in these animals between the kidney concentrations of chemerin, MIP1a, and Bax and the concentrations of Cd in the blood, urine, and kidney (Table 2).

### 2.7. Mutual Relationships between the Investigated Markers of the Kidney Status

In the female rats that were not administered with AM (the control group and the Cd_1_ and Cd_5_ groups), mutually positive relationships were noted between each of the investigated markers of renal tubular damage (KIM-1, β2-MG, NAG, and ALP), ACR, and PCR (Table 3). The creatinine clearance was negatively correlated with the urinary β2-MG, NAG, ALP, ACR, and PCR, as well as urea the concentration in the serum, and positively correlated with the urinary concentrations of uric acid and urea (Table 3). Positive relationships also occurred between the markers of kidney damage determined in the urine (KIM-1, β2-MG, NAG, ALP, ACR, and PCR) and the serum concentrations of uric acid and urea, except for a lack of relationship between the serum concentration of uric acid and ALP activity and PCR (Table 3). There was no relationship between the serum and urinary concentrations of uric acid and urea (Table 3). The urinary activities of NAG and ALP, ACR, creatinine clearance, and the concentrations of uric acid and urea in the serum positively correlated with the kidney concentration of chemerin (Table 3).

Unlike the female rats that were not administered with AM, in the animals that received this extract (alone and during the exposure to Cd), only a few mutual dependencies were noted between the evaluated markers of tubular and glomerular damage (Table 3). Moreover, negative dependencies occurred between β2-MG, NAG, ALP, ACR, and PCR and the kidney concentrations of MIP1a and/or Bax (Table 3).

In both animals administered or not with AM, positive relationships occurred between the kidney concentrations of chemerin, MIP1a, and Bax (Table 3).

## 3. Discussion

The present article is the first report, from studies carried out in an experimental animal model, that even low-level, long-term exposure to Cd poses a substantial risk of damage to the kidney, whereas the intake of an extract from the berries of *A. melanocarpa* considerably protects against this outcome. Since the study was conducted in an in vivo model that well reflects the current levels of Cd exposure in the worldwide general population, it can be concluded that lifetime environmental exposure to this heavy metal in industrialized countries is a risk factor for kidney damage. Moreover, the article presents a proposal for an effective protective strategy against this organ injury due to long-term low-to-moderate intoxication with this xenobiotic.

The kidney, as the main organ of Cd accumulation in the body and the organ responsible for its detoxification and elimination, is especially vulnerable to damage caused by this xenobiotic [2,4,5,6,8,12,14]. Because Cd is very slowly eliminated from the body, it gradually accumulates in the kidney during chronic intoxication, mainly in the form of complexes with a low-molecular-weight protein—metallothionein (MT) [2,13,25]. The binding of Cd ions (Cd^2+^) in the kidney cells by MT results in the formation of non-toxic (in the intracellular space) complexes of this element with MT (Cd-MT complexes), which is the process of detoxification of this heavy metal. However, Cd-MT complexes are characterized by a short lifespan (about 3 days) and are decomposed via the release of Cd^2+^ ions, which further induce the synthesis of MT and bind to this protein. Nevertheless, the ability of the kidney to biosynthesize MT and accumulate Cd in the form of non-toxic Cd-MT complexes is limited [2,13,25]. Thus, repeated exposure to this toxic heavy metal creates a threat to kidney health [1,2,4,12,14], and as shown in the present study, even low-level intoxication with this xenobiotic can result in organ damage.

As performed in the present investigation, measurements of the sensitive biomarkers of kidney status together with histological evaluation of this organ revealed that repeated, low-level, and moderate exposure to Cd resulted in abnormalities in the function and morphological structure of kidney tubules and glomeruli. The injury to tubules exceeded damage to the glomeruli, and the first signs of the damaging impact of this xenobiotic on the kidneys were found as early as after 3 months of low-level exposure. Compared to the proper values determined in the control animals, the elevated concentrations of KIM-1 and β2-MG and the activities of NAG and ALP in the urine reflect tubular damage, which gradually progressed with the duration of exposure, especially at the higher investigated levels of intoxication. It is important to emphasize that although there were no differences in the values of the biomarkers of tubular damage between the Cd_1_ and Cd_5_ groups at particular time points, the changes in the values of these parameters occurred earlier at the higher exposure, except for KIM-1. The positive relationships found between almost all determined indices of renal tubular damage and Cd concentration in the blood, urine, and kidneys of the animals exposed to this element in trace-to-moderate amounts show that the extent of tubular damage progressed with the increasing body burden of this xenobiotic. Moreover, histopathological studies also revealed that the changes in the morphological structure of the tubules were more advanced at a higher level of exposure to Cd. Similar to those noted in the Cd_1_ and Cd_5_ groups, pathological changes in the histological structure of the kidney were also reported by other authors, however, at higher levels of exposure to this xenobiotic [33,34,35,36,37,38].

The finding that, at both levels of exposure to Cd, the first biomarker whose values were enhanced already after 3 months was KIM-1 and that this effect occurred from a few to several months earlier than changes in the values of β2-MG and NAG, which are commonly considered sensitive markers of this heavy metal nephrotoxicity [2,8,13,14,15], shows that this parameter appears to be the earliest biomarker of this xenobiotic-induced tubular damage. KIM-1 is a transmembrane glycoprotein localized on the epithelial cells of the proximal tubules. Cd undergoes accumulation in the cells of the tubular epithelium, and once damage to these cells occurs due to the toxic action of Cd^2+^ ions, KIM-1 is shed into the urine [39]. Although an increase in the concentration of KIM-1 in the urine as a result of exposure to Cd has been reported in numerous studies in humans and experimental animals [2,13,39,40,41]⁠, according to some authors, the usefulness of this marker during low-level exposure to this toxic element may be limited [13,40]. However, our study provides credible evidence that KIM-1 can be an early marker of tubular damage during such exposure, and thus, it is worth determining the impact of Cd on kidney status. In humans environmentally exposed to Cd, the higher prevalence rate of increased urinary concentration of KIM-1 has been shown to occur at this heavy metal concentration in the urine higher than 1 μg/g creatinine [39]. Our study shows that an increased concentration of this biomarker can be noted already after a relatively short exposure (3 months in the experimental model) and at markedly lower levels (several times) than in human Cd concentrations in the urine (0.0852–0.2820 μg/g creatinine) (Table S15) [17].

Like KIM-1, NAG is another biomarker of Cd-induced cytotoxic damage to the renal tubules. It is a lysosomal enzyme abundantly present in the epithelial cells of the proximal tubules. Its activity is low under physiological conditions, but it increases due to Cd-induced injury of the tubular cells [2,8,13,37,39,42,43]. Thus, the increased activity of NAG noted in the animals fed with fodder containing 1 and 5 mg Cd/kg indicates proximal tubular damage. The Cd concentration in the urine of female rats at which the increase in the activity of NAG was first detected (median 0.2403 μg/g creatinine; range 0.1912–0.4959 μg/g creatinine in the Cd_1_ group and median 0.3570 μg/g creatinine; range 0.2120–0.5084 μg/g creatinine in the Cd_5_ group) (Table S12) is similar to the heavy metal concentration at which an increased activity of this enzyme was reported in humans (0.38 μg Cd/g creatinine in men and 0.42 μg Cd/g creatinine in women) [4]. Apart from KIM-1 and NAG, the urinary activity of ALP is also a biomarker of cytotoxic damage to the proximal tubules. This enzyme is present in the cells of the brush border of the epithelium of the proximal tubules, and thus, damage to these cells is reflected in the increased activity of this enzyme in the urine [13]. Increased activity of ALP in the urine has been found in workers occupationally exposed to Cd who have high concentrations of this xenobiotic in the urine [37] and in experimental animals [43]; however, this biomarker is not commonly used in epidemiological studies. The fact that in the present study, at both levels of treatment with Cd, the increase in the activity of ALP in the urine occurred earlier (after 4 and 10 months of low-level and moderate exposure, respectively) than the changes of commonly used sensitive markers, such as β2-MG and NAG, indicates that the determination of ALP may be useful in monitoring the impact of low-to-moderate exposure to Cd on kidney status.

At both levels of exposure to Cd, the concentration of β2-MG in the urine increased for the first time two months later than when the increase in the activity of NAG occurred, confirming that NAG is a more sensitive biomarker for Cd-induced renal tubular damage than β2-MG [14]. β2-MG is a low-molecular-weight protein present on the surface of almost all nucleated cells and routinely shed by these cells into the blood. Under physiological conditions, this protein passes by the glomeruli and is reabsorbed by the proximal tubules. Thus, it is present in the urine only in low concentrations; however, Cd-induced damage to the reabsorptive function of tubules results in an increase in its amount in the urine [4,14]. The increase in the concentration of β2-MG in the urine of female rats exposed to Cd reflects the destroyed reabsorptive function of the renal proximal tubules. Based on our results, it can be concluded that the reabsorptive function of the renal tubules may start to weaken at such low Cd concentrations in the urine as 0.1850–0.3110 μg/g creatinine (Table S12). In humans, β2-MG was found in the urine in enhanced amounts at Cd concentrations, reaching 0.38 μg/g creatinine in men and 0.42 μg/g creatinine in women [4].

The Cd-induced increase in ACR, PCR, and the serum concentrations of uric acid and urea, as well as the decrease in creatinine clearance, reflect the impairment of glomerular filtration. Our recently performed overview of the available literature data allowed for the conclusion that albuminuria and GFR (estimated in the present study based on ACR and creatinine clearance, respectively) are sensitive markers of Cd-induced glomerular damage and that LOAELs of the Cd concentration in the blood and urine for glomerular dysfunction (albuminuria and decreased GFR) in the general population are >0.18 μg/L and >0.27 μg/g creatinine, respectively [2,4,5]. In the present study, the first obvious signs of glomerular damage (increased PCR that occurred after 6 months and remained until the end of the 24-month study) were observed at the urinary concentration of the toxic element, ranging from 0.1485 to 0.3397 μg/g creatinine (median 0.2311 μg/g creatinine). The 22–35% reduction in creatinine clearance (reflecting the amount of creatinine filtered in the glomeruli per unit of time) and the increased concentration of uric acid and/or urea in the serum after the 17–24-month exposure to the 5 mg Cd/kg feed indicate significant deterioration in the GFR. Although there were no differences in the values of particular markers of glomerular damage between the Cd_1_ and Cd_5_ groups, the fact that at the exposure to the 1 mg Cd/kg feed, the creatinine clearance as well as the serum concentration of uric acid and urea were unaffected shows that the damaging impact of this heavy metal on the kidney was more serious at the exposure to the 5 mg Cd/kg feed. Moreover, the histopathological study revealed more advanced glomerulonephritis at the higher of the investigated levels of exposure to Cd. Low-level exposure to this xenobiotic would be expected to not affect creatinine clearance and the serum concentrations of uric acid and urea; however, the finding that the increase in the urinary excretion of total protein (increased PCR) at both levels of exposure to Cd occurred after 6 months and exceeded (for 10–12 months) an occurrence of the enhancement in the excretion of albumin (increased ACR) was unexpected, especially at low-level exposure. The elevation in the values of KIM-1 and β2-MG and enzymatic proteins such as NAG and ALP noted in Cd-exposed rats was not high enough to explain the increase in PCR; however, the presence in the urine of other proteins (e.g., α1-microglobulin, Clara cell protein 16, and MT) might also be enhanced [2,8,13,14]. The positive relationships between Cd concentration in the blood, urine, and kidney and the urinary ACR and PCR in the animals that did not receive AM during trace-to-moderate exposure to Cd indicate that the risk of Cd-induced proteinuria, including albuminuria, increased with the increasing body burden of Cd. Albumin is the main plasma protein physiologically present in the blood. Under proper kidney function, only trace amounts of this protein occur in the urine, but when glomerular damage occurs, its concentration in the urine increases [2].

Detailed analysis of the changes in the values of particular biomarkers of the kidney status during the 24-month exposure to Cd showed that the kidney injury developed and progressed with the duration of the exposure to this xenobiotic, leading to both tubular and glomerular damage at relatively low concentrations of this xenobiotic in the blood and urine and its low accumulation in the kidney (Table S15). Histopathological studies confirmed the damaging impact of Cd on kidney morphology, including both the injury of tubules and the development of glomerulonephritis due to lifelong, even low-level, exposure to this xenobiotic. It is important to emphasize that although the statistical analysis revealed no difference in Cd concentration in the urine or biomarkers of kidney damage between the Cd_1_ and Cd_5_ groups, the measurements of biochemical parameters and microscopic assessment of the morphological structure of this organ allowed for the conclusion that the damaging impact of Cd on the kidney at the higher exposure occurred earlier and was more advanced at the same exposure duration than at the lower level of exposure. The lack of statistically significant differences in the values of biomarkers of Cd nephrotoxicity between the Cd_1_ and Cd_5_ groups may be explained by the lack of difference in this toxic element’s concentration in the urine (the most useful biomarker of long-term exposure to Cd) between these two groups. Moreover, the lack of differences in both the values of the estimated biomarkers of kidney status and the Cd concentration in the urine between these groups might also result from a relatively wide range of values in terms of particular parameters.

The fact that in the experimental model of human exposure to Cd that we created, the 24-month exposure to the 1 mg Cd/kg feed resulted in cytotoxic damage to the kidney tubules, weakened their resorptive function, and led to glomerulonephritis, allows for the conclusion that lifelong exposure to this toxic heavy metal may create a risk for kidney damage in the general population. Even under low environmental exposure, Cd gradually accumulates in the kidney during a person’s lifetime and reaches a peak concentration in this organ at around 60 years of age [44,45]⁠. In the female rats maintained on fodder containing only trace amounts of Cd (0.098 mg/kg) and those fed with the 1 mg Cd/kg diet, the accumulation of this element in the kidneys increased throughout the experiment and reached its peak after 24 months (0.0844 ± 0.0357 and 1.981 ± 0.5089 μg/g wet weight (w.w.), respectively) [17], when the age of the animals was equivalent to the human age of 60 years [46]. The accumulation of Cd in the kidneys during the exposure to 5 mg Cd/kg reached a peak after 17 months (10.77 ± 1.936 μg/g w.w.), corresponding to approximately 45 human years [46], and a plateau was reached thereafter [17].

Detailed analysis of the results of the present study shows that even under low-level exposure, the first signs of the damaging impact of Cd on the kidney tubules may occur already at a young age (i.e., after 3 months of the experiment, corresponding to the human age of around 18 [46]), while at the stage of adulthood (i.e., after 10–17 months of the study, corresponding to 30–45 years in humans [46]), the damage to both tubules and glomeruli was clearly evident, whereas in the elderly (i.e., after 24 months of the study), tubular damage intensified and glomerulonephritis developed. The finding that even low-level exposure to Cd has a detrimental effect on the kidney that progresses with exposure duration and may lead to destroying kidney function and pathological changes in this organ structure is a very important result of the study. Revealing that these effects occur at this heavy metal concentration in the urine (0.0852–0.2820 μg/g creatinine) within the lower range of values noted in the worldwide general population confirms our recently published [2] conclusion made based on the overview of literature data that even low-level environmental exposure currently occurring in developed countries is a risk factor for kidney damage.

Although the toxic impact of Cd on the kidneys has been known for a long time and has been the subject of many studies, the mechanism of the nephrotoxic action of this xenobiotic, as well as the risk of this organ damage under low-level exposure, have not been sufficiently studied (for a review, see [2]). In the available literature, there are numerous studies on the damaging impact of Cd on the kidney conducted in laboratory animals; however, the used experimental models do not correspond to human exposure in terms of the dose (moderate, high, and even very high doses were administered) of this xenobiotic and the route of intoxication [33,34,35,36,37,38,40,47,48,49]. To our knowledge, the experimental model we created is the only in vivo model constructed so far that well reflects human environmental exposure, and, therefore, we discuss our findings in the light of data from epidemiological studies. According to current knowledge, the mechanism of the nephrotoxic action of Cd is multidirectional and mainly involves the induction of oxidative stress and oxidative lesions of cellular macromolecules and cellular organelles, the development of inflammatory processes, the stimulation of cell proliferation, and the induction of epigenetic changes [2,4,8]. As noted in the present study, increased concentrations of chemerin and Bax in the renal tissue and changes in the microscopic image of the kidney show that the mechanism of the toxic impact of Cd at low-to-moderate exposure is related to the induction of inflammatory status and the stimulation of apoptotic and proliferative processes [35,37,40]. The present study was focused primarily on understanding whether low-level chronic exposure creates a risk for kidney damage and if this effect may be counteracted by Aronia extract. However, our research project also includes an explanation of the mechanisms of both the nephrotoxic action of Cd and the nephroprotective impact of the extract, and the findings will be published soon.

The most important and new finding of the present study is revealing the possibility of protection from the damaging impact of Cd on the kidney via the administration of chokeberry extract and providing further evidence of the beneficial impact of Aronia products to counteract the harmful effect of exposure to this xenobiotic. The finding that the intake of chokeberry extract during low-level and moderate exposure to Cd almost completely protected from changes in the biochemical biomarkers of this xenobiotic-induced tubular and glomerular damage and weakened pathological changes in the histological structure of the renal tissue suggests that Aronia berry products are a good strategy for protecting against kidney damage caused by exposure to this xenobiotic. Since even low-level chronic exposure to Cd, which nowadays is increasingly inevitable in industrialized countries, creates a risk for kidney damage [1,2,4,5,6,8,12,14], the findings of this study have not only scientific value but are also very important from a public health perspective and have practical implications.

Considering our previous findings taken from the experimental model [17,18,19,20,21,22,23,24,25,26] and the chemical composition of the chokeberry extract [21,27,28,30,32], the protective impact of AM regarding Cd nephrotoxicity revealed in the present study may be explained by the direct action of the extract ingredients, especially polyphenolic compounds, as well as their interaction with Cd. It has been revealed that the administration of AM to the animals treated with 5 mg Cd/kg feed decreased the apparent absorption (by 14–17%) and retention in the body (by 14–17%) and increased urinary excretion (by 21–39%) of this toxic element, resulting in lower total accumulation in the internal organs (by 20–29%) (mainly in the kidneys and liver—by 20–29%—but also in the spleen, heart, and brain, by 15–17%) and bone tissue (by 12–25%), as well as a lower concentration in the blood (by 10–19%) (Table S15) [17]. The Cd concentration in the kidneys of the rats co-administered with AM and the 5 mg Cd/kg feed for 3–24 months was lower by 6.5–13% than in the animals that were not supplemented with the extract during the treatment with this toxic element (Table S15). However, at exposure to the 1 mg Cd/kg feed, the beneficial effect of AM was very slight—only a temporary decrease in the apparent absorption and retention of Cd (by 14% and 13%, respectively, after 10 months) and its concentration in the liver (by 29% after 3 months) and kidney (by 33% and 37% after 3 and 24 months, respectively) was noted (Table S15) [17].

The measurements of Cd concentration in the urine carried out in the present study every other month during the 24-month experiment also disclosed that the administration of AM to the animals maintained on fodder containing 5 mg Cd/kg led to an increase in the urinary excretion of Cd. The revealed protective impact of AM on the function and structure of the kidney, especially at moderate exposure, may, at least partially, be explained by the lower concentration of Cd in the blood and kidney and its increased urinary excretion (Table S15) [17]. The finding that the administration of AM during the exposure to the 5 mg Cd/kg feed, similar to the case of low-level exposure, almost entirely protected the kidney from the damaging impact of Cd on the kidney may be explained by the markedly lower body burden of Cd. Moreover, the fact that in the animals that received AM during the trace-to-moderate levels of exposure to Cd, there were almost no dependencies between this heavy metal concentration in the blood, urine, and kidney (unlike the animals that did not receive the extract) may suggest that the decrease in the body burden of Cd was not the only cause of the nephroprotective effect of the extract. The ability of the chokeberry extract to decrease the body burden of Cd resulted, at least to some extent, from the high content of polyphenolic compounds, which, because of the presence of hydroxyl (-OH) groups, are able to form complexes with ions of divalent toxic metals, including Cd^2+^ ions [17,32]. Polyphenols can form complexes with Cd^2+^ ions in the gastrointestinal tract, preventing their absorption in this way, as well as in the extracellular fluids, facilitating their elimination via the urine, and protecting against their retention in the body and toxic effect [17,30,32].

Taking into account the known health-promoting properties of chokeberries [27,29,30,31,49,50], the results of the present study, as well as our previous findings in these animals [17,18,19,20,21,22,23,24,25,26], the protective impact of AM against Cd nephrotoxicity can also be explained by the antioxidative, anti-inflammatory, antiapoptotic, and antiproliferative properties of the extract ingredients, especially polyphenolic compounds [27,29,30,31]. Although, at this stage of our study, we are unable to explain these mechanisms, the fact that the administration of AM under both levels of exposure to Cd led to a decrease in the concentration of chemerin, MIP1a, and Bax compared to the animals that did not receive the extract shows the involvement of the anti-inflammatory and antiapoptotic potential of the extract. These, together with the antiproliferative effect of AM revealed in histopathological studies and the lower body burden of Cd, including its lower accumulation in the kidneys, may, to some extent, explain the protective impact of the extract. Further possible mechanisms, especially the strong antioxidative potential of chokeberries [22,29] are also the subject of our research project, and the findings will be published soon. Polyphenolic compounds are the main group of chokeberry extract ingredients characterized by numerous healthful properties [27,29,30,31]; however, it is important to point out that the nephroprotective effect of AM might also result from the presence of other components that are effective in reducing Cd toxicity, such as β-carotene, triterpenes, fiber, pectin, ascorbic acid (vitamin C), alpha-tocopherol (vitamin E), and essential bioelements, such as Zn or selenium [29,30,31].

In the available literature, there is no data on the possibility of the protective impact of Aronia products on Cd-induced kidney damage. Only Kowalczyk et al. [49] have revealed that oral administration of anthocyanins from the berries of *A. melanocarpa* at a dose of 10 mg/kg b.w. during a 30-day treatment with cadmium chloride 2.5-hydrate (CdCl_2_ × 2.5 H_2_O) at a dose of 4 μg/kg b.w. resulted in a decrease in Cd accumulation in the kidney and protected against this heavy-metal-induced increase in the concentration of urea in the serum. Moreover, there are some reports from experimental studies showing that some other compounds, such as curcumin [43], carvacrol [45], N-acetyl-L-cysteine (NAC) [47], and calcium antagonists such as nimodipine and chlorpromazine [48], can protect from Cd nephrotoxicity; none of these studies, however, was as comprehensive as the present study and did not evaluate the impact of exposure comparable to the exposure of the general population. The present study is the only one so far to indicate that the consumption of Aronia products may be an effective strategy in protecting against kidney damage due to low-to-moderate chronic exposure to Cd. Moreover, this article is our next report, providing evidence for the multidirectional protective impact of chokeberry ingredients in counteracting the negative health outcomes of exposure to Cd.

It is important to underline that the fact that in the animals that received AM alone, the values of all biomarkers of kidney status were within the proper values noted in the control group shows that the enhanced daily intake of polyphenols in the absence of exposure to Cd has no negative impact on the kidney. The slight changes in the morphological picture of the kidney noted in single animals after 24 months of the study may be related to aging-related changes in this organ. The same might be a cause of the slight changes evident in the morphological picture of the kidney in the control group. In the available literature, we have found no data on the unfavorable impact of the consumption of Aronia products on kidney status. Moreover, it has been reported that supplementation with chokeberry juice in type 2 diabetic patients decreased the concentration of creatinine in the urine after 3 months but not after 6 months and had no impact on the concentration of creatinine and urea in the serum [50].

We are fully aware of the limitations of our research and its accomplishments. Since female rats were used in the trial because they are more prone to Cd toxicity than male rats, our findings apply to female kidneys. That is why further studies on both the toxicity of Cd in the model well reflecting human exposure to this heavy metal and the protective properties of AM during this exposure on male kidneys are necessary. Moreover, at this stage of our research, we are unable to explain some results (e.g., why the increase in PCR exceeded the increase in ACR) and the mechanisms of the nephrotoxic impact of Cd at low-to-moderate exposure and the nephroprotective effect of AM; however, we are currently conducting studies in this area and will publish the results soon. We are aware that extrapolation of our findings regarding Cd concentration in the blood and urine and its accumulation in the kidneys of rats to humans could be inaccurate and should be approached with caution; however, our study provides the only attempt to explore this process in vivo considering a lifetime exposure level comparable to that currently noted in the worldwide general population in industrialized countries. Despite some limitations, the results of this study indicate that Aronia products appear to be an important strategy in preventing the harmful health effects of environmental exposure to Cd. However, further studies are needed in humans to confirm their effectiveness in protecting the kidneys in the general population.

## 4. Materials and Methods

### 4.1. Animals

In total, 192 3–4 week-old female Wistar rats (Hannover Wistar rats, bred according to the Charles River International Genetic Standardization Program—Crl: WI (Han)) from a licensed breeding facility (Laboratory Animal House, Brwinów, Poland) were used in the study. The rats were given 5 days to acclimate to the experimental environment before the study began. Throughout the experiment, the animals were kept under standard conditions (temperature 22 ± 2 °C, relative humidity 50 ± 10%, and 12-h light/dark cycle) with free access to feed and drinking water.

### 4.2. Feed Containing Cd

The feed containing Cd at concentrations of 1 and 5 mg/kg was provided by Label Food “Morawski” (Kcynia, Poland). It was prepared by adding CdCl_2_ × 2.5 H_2_O (POCh, Gliwice, Poland) to the ingredients of the Labofeed H diet (given as a breeding diet for the first 3 months of the experiment) and the standard Labofeed B diet (used as the maintenance diet from the beginning of the 4th month until the end of the study).

The procedure was carried out to determine the homogeneity of Cd content in the Labofeed diets, and the results showed that the concentration of this element in these diets (1.09 ± 0.13 mg/kg and 4.92 ± 0.53 mg/kg; mean ± standard deviation—SD) was exactly in line with the certified values (1 and 5 mg Cd/kg, respectively) [17]. The mean concentration of Cd in the standard Labofeed fodder (maintenance and breeding diets) without the addition of CdCl_2_ × 2.5 H_2_O reached 0.0584 ± 0.0049 mg/kg [17].

### 4.3. Aronia melanocarpa L. Extract

The lyophilized chokeberry extract, in powdered form, provided by Adamed Consumer Healthcare (Tuszyn, Poland), was used. According to the manufacturer’s (Adamed Consumer Healthcare, Tuszyn, Poland) declaration (Certificate KJ 4/2010; Butch No. M100703), the extract contained 65.74% polyphenolic compounds, including 18.65% anthocyanins. The total content of polyphenols in the powdered extract determined in our laboratory was 612.40 ± 3.33 mg/g (mean ± standard error—SE), and the polyphenolic profile (Figure S1) of the extract was as follows: total anthocyanins (202.28 ± 1.28 mg/g); derivatives of cyanidin (cyanidin 3-O-β-galactoside— 80.07 ± 1.05 mg/g; cyanidin 3-O-α-arabinoside—33.21 ± 0.01 mg/g; cyanidin 3-O-β-glucoside—3.68 ± 0.01 mg/g); total proanthocyanidins (129.87 ± 1.12 mg/g); total phenolic acids (110.92 ± 0.89 mg/g); chlorogenic acid (68.32 ± 0.08 mg/g); and total flavonoids (21.94 ± 0.98 mg/g) [21]. The extract also contained other ingredients such as carotenoids, minerals, pectins, sugar, sugar alcohols, phytosterols, triterpenes, and vitamins, as well as 6.1% water (producer data, [27,28]).

To prepare the 0.1% aqueous solution of the extract (AM) for administration to animals, 1 g of the powdered extract by Adamed was dissolved in 1 L of redistilled water. The solution was prepared daily and was stable for at least 24 h. One mL of the solution contained 0.612 ± 0.003 mg of polyphenols [17].

### 4.4. Design of the Study

The experimental protocol was approved by the Local Ethics Committee for Animal Experiments in Bialystok (Poland; permission number 60/2009 on 21 September 2009). All procedures using animals were carried out in accordance with institutional guidelines, ethical standards, and the International Guide for the Use of Animals in Biomedical Research.

The rats were randomly divided into the following six experimental groups, each consisting of 32 animals:

- Control group: the rats were maintained on the standard Labofeed diet (0.0584 ± 0.0049 mg Cd/kg) and drinking water (redistilled water containing < 0.05 μg Cd/L) without the addition of the extract from the berries of *A melanocarpa* L.
- AM group: the animals received AM (0.1% aqueous solution of the extract from the berries of *A. melanocarpa*) as the only drinking fluid.
- Cd_1_ group: the rats were fed a diet containing 1 mg Cd/kg and received redistilled water as drinking fluid.
- Cd_1_+AM group: the animals were fed AM as the only drinking fluid for the entire duration of exposure to Cd at a concentration of 1 mg/kg feed.
- Cd_5_ group: the rats were treated with Cd at a concentration of 5 mg/kg feed and received redistilled water as drinking fluid.
- Cd_5_+AM group: the animals were exposed to Cd at a concentration of 5 mg/kg feed and received AM as the only drinking fluid.

The experiment lasted up to 24 months. During the 24-month study, no unfavorable health outcomes were observed in all groups; however, one animal from the AM group, Cd_1_ group, and Cd_5_ group died spontaneously between the 18th and 20th month [17]. Eight females of each group, except for seven animals after 24 months in the AM, Cd_1_, and Cd_5_ groups, were necropsied after 3, 10, 17, and 24 months. At the beginning of the study (before starting the administration of Cd and/or AM) and at the end of every other month of the 24-month study, the 24-h urine was collected from the same eight animals in each group (except for seven females after 20, 22, and 24 months in the AM, Cd_1_, and Cd_5_ groups). The urine was also collected from eight rats from each group subjected to necropsy after 3, 10, and 17 months. To perform the 24-h urine collection, the rats were housed individually in metabolic cages. During this time, the animals had free access to feed and drinking fluid (Labofeed diet with or without Cd and redistilled water or AM, depending on the experimental group). Immediately after collection, the urine was centrifuged (MPW-350R centrifuge, Medical Instruments; Warsaw, Poland), and its volume was determined.

The exposure to the 1 or 5 mg Cd/kg feed reflects low or moderate levels of environmental human exposure, respectively, which was confirmed by analyzing the Cd concentration in the blood (0.1030–0.3240 μg Cd/L in the Cd_1_ group and 0.7350–1.3320 μg Cd/L in the Cd_5_ group) and urine (0.0852–0.2820 and 0.2839–0.6949 μg Cd/g creatinine, respectively) of the rats [17] being within the lower range of values currently noted in the general population of industrialized countries (0.02–4.40 μg/L in the blood and 0.04–3.39 μg/g creatinine in the urine (for a review, see [2]). The concentration of Cd in the blood (0.0330–0.1290 and 0.0330–0.1360 μg Cd/L in the control group and AM group, respectively) and urine (0.0764–0.2013 and 0.0730–0.1712 μg Cd/g creatinine in the control group and AM group, respectively), noted in the groups non-exposed to this heavy metal, was irrelevant compared to the other experimental groups [17]. The selection of female rats over male specimens was conditioned by the higher susceptibility of females to the toxic action of Cd, which has been confirmed both in animal and human studies [8].

Throughout the study, the daily feed and fluid intakes, as well as body weight gain, did not differ between the experimental groups [17]. Based on the measurements of the daily consumption of feed and AM, the intake of Cd, as well as the extract and polyphenols, were calculated (Table 4). The daily intake of Cd and polyphenols did not differ between the experimental groups regardless of whether Cd and AM were administered separately or together (Table 4). The daily intake of polyphenolic compounds in the animals that received AM as the only drinking fluid (Table 4) was several times higher compared to the average intake of these compounds among the general population worldwide. According to the available literature data [31], the daily intake of polyphenols in humans ranges from about 800 mg to over 1700 mg, reaching an average of 1000 mg (14.29 mg/kg b.w. assuming a mean body weight of 70 kg).

Following the 24-h urine collection performed after 3, 10, 17, and 24 months of the experiment, the rats were deprived of food overnight and then subjected to intraperitoneal anesthesia with barbiturate (Morbital, 30 mg/kg b.w.; Biowet; Pulawy, Poland), under which the whole blood was taken via cardiac puncture with and without anticoagulant (heparin), and various organs and tissues, including both kidneys, were dissected. The kidneys, immediately after collection, were rinsed multiple times with ice-cold 0.9% sodium chloride (physiological saline) and gently dried on the filter paper. Next, these organs were weighed with an analytical balance (OHAUS^®^, Nanikon, Switzerland; accuracy to 0.0001 g). The left kidney was sectioned in the longitudinal plane into two parts. One of these parts of four animals from each group after 3, 10, 17, and 24 months was subjected to histopathological examination. The second part of the left organ was used for other measurements, including the determination of Cd concentration [17,25,26]. The biological material that was not used immediately was stored frozen (−70 °C) until assayed.

**Figure 13:**
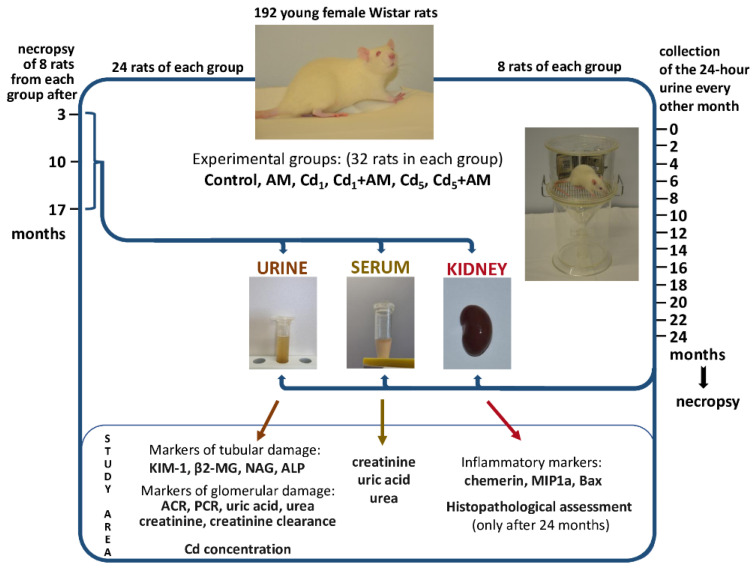
A schematic representation of the experimental model and range of measurements performed in the present study. The animals were treated with cadmium (Cd) in the diet at a concentration of 0, 1, or 5 mg/kg (Control, Cd_1_, and Cd_5_ groups) and/or 0.1% extract from the berries of *Aronia melanocarpa* L. (AM, Cd_1_+AM, and Cd_5_+AM groups) for 3, 10, 17, and 24 months. β2-MG, beta2-microglobulin; ACR, albumin concentration in the urine adjusted for creatinine concentration; ALP, alkaline phosphatase; Bax, Bcl-2-associated X protein; KIM-1, kidney injury molecule 1; MIP1a, macrophage inflammatory protein-1 alpha; NAG, N-acetyl-β-D-glucosaminidase; PCR, total protein concentration in the urine adjusted for creatinine concentration. The appropriate parameters, except for Cd in the urine, were determined in the serum, urine, and kidney after 3, 10, 17, and 24 months. Moreover, β2-MG, NAG, ALP, ACR, and PCR, as well as Cd, were evaluated every other month of the 24-month study and before its beginning.

The experimental model has been reported in detail [17,18,19,20,21,22,23,25,26]. A schematic representation of the model and range of measurements performed in the present paper are presented in Figure 13.

### 4.5. Analytical Methods

All measurements using commercial kits were performed according to the recommendations of the producers, and the precision of these measurements was expressed as the intra- and/or inter-assay coefficient of variation (CV). The spectrophotometers MULTISCAN GO (Thermo Scientific, Vantaa, Finland), Epoch (Bio Tek Instruments, Inc., Winooski, VT, USA), and Specord 50 Plus (Analityk Jena, Jena, Germany) were used for the quantification of the determined variables.

#### 4.5.1. Measurements of the Markers of Kidney Status in the Urine and Serum

##### Biomarkers of Tubular Damage

The measurement of the concentration of KIM-1 was performed with the KIM-1 ELISA Kit (Catalog No. MBS355395) produced by MyBioSource, Inc. (San Diego, CA, USA). Sandwich enzyme-linked immunosorbent assay (ELISA) technology served as the foundation for this kit. Purified, horseradish peroxidase (HRP)-conjugated anti-KIM-1 antibody pre-coated on a plate was used as a detecting antibody. Following the addition, mixing, and incubation of the standards and tested samples in the wells, the unbound conjugate was removed from the plate using a wash buffer. The HRP enzymatic reaction was visualized using a chromogenic reagent. The reagent was catalyzed using HRP to create a blue color product that became yellow after the addition of an acidic stop solution. The absorbance of standards and tested samples was measured at 450 nm using a microplate reader, and the concentration of KIM-1 in the tested samples was automatically quantified from the calibration curve. The intra- and inter-assay CVs for this kit were <3% and 2%, respectively.

The concentration of β2-MG and the activity of NAG in the urine were determined with the use of the B2M ELISA Kit (Catalog No. E0260r) and the Rat N-acetyl-beta-D-glucosaminidase ELISA Kit (Catalog No. E0069r), respectively, by EIAAB Science Inc. (Wuhan, China). Standards and tested samples were added to the wells pre-coated with biotin-conjugated antibodies specific for the target protein (β2-MG or NAG), and after incubation with detection reagents and substrate addition, stop solution was added, producing a yellow-colored product (450 nm). The concentration of β2-MG and the activity of NAG were automatically quantified from their respective calibration curves. The intra- and inter-assay CVs were <4.5% and 3%, respectively, for β2-MG and <5.4% and 2.4%, respectively, for NAG.

The activity of ALP in the urine was assessed using the kit (Catalog No. 1-221-0150) produced by BioMaxima (Lublin, Poland). This method was based on the hydrolysis of colorless p-nitrophenyl phosphate to p-nitrophenol and inorganic phosphate. The rate of an increase in the absorbance of the yellow p-nitrophenol, measured at 405 nm, is proportional to the activity of ALP. The activity of this enzyme assayed by us in the reference BioNorm serum (Catalog No. 1-801-0020; BioMaxima, Lublin, Poland) was 101.7 ± 2.899 U/L (mean ± SD) and agreed with the reference value (86.9–133 U/L).

The concentrations of KIM-1 and β2-MG in the urine were expressed in calculations per creatinine concentration, whereas the activities of NAG and ALP were expressed in international units per litter of the 24-h urine (U/L).

##### Biomarkers of Glomerular Damage

The concentrations of albumin and total protein in the urine were determined with the use of kits (Catalog No. 1-003-0200 and No. 1-008-0200, respectively) produced by BioMaxima (Lublin, Poland). The measurement of the concentration of albumin was based on the end-point method with bromocresol green. This compound binds to albumin, producing a colored complex. The intensity of the color of the complex, measured photometrically (630 nm), is proportional to the concentration of albumin in a tested sample. The concentration of albumin determined by us in the reference BioNorm serum (Catalog No. 1-801-0020; BioMaxima) was 4.45 ± 0.184 g/100 mL (mean ± SD) and agreed with the reference value (3.89–5.27 g/100 mL). The concentration of total protein was determined by the biuret method. The principle of the method is based on a reaction of peptide bonds of protein with ions of copper(II), leading to the formation of a blue complex whose absorption (546 nm) is proportional to the amount of protein present in a tested sample. The concentration of total protein in the control BioNorm serum (Catalog No. 1-801-0020; BioMaxima) determined in our laboratory (5.89 ± 0.34 g/100 mL; mean ± SD) was within the range of reference values provided by the producer (5.26–6.70 g/100 mL). The concentrations of albumin and total protein in the urine were adjusted for creatinine concentrations (ACR and PCR, respectively).

The determination of creatinine concentration in the serum and urine was performed using a kinetic method (Jaffe’s method) with the use of the BioMaxima kit (Catalog No. 1-038-0300; Lublin, Poland) based on the reaction between creatinine and picric acid in alkaline pH, leading to the formation of an orange-red complex, the intensity of which color is proportional to the concentration of creatinine in a tested sample. Based on the concentration of creatinine in the serum and urine and the volume of the 24-h urine, creatinine clearance was calculated. The concentration of creatinine determined by us in the control BioNorm serum (Catalog No. 1-801-0020; BioMaxima) reached 1.270 ± 0.183 mg/100 mL (mean ± SD) and was within the reference range (1.04–1.50 mg/100 mL).

The concentrations of urea and uric acid in the serum and urine were determined with the use of kits (Catalog No. 1-480-0300 and 1-045-0200, respectively) produced by BioMaxima (Lublin, Poland). The measurement of urea concentration was performed using a kinetic enzymatic method with urease and glutamate dehydrogenase. Urease hydrolyzes urea into ammonia and carbon dioxide. In the presence of glutamate dehydrogenase, glutamate is formed from ammonia and 2-oxoglutarate, and at the same time, nicotinamide adenine dinucleotide (NADH) is oxidized. The rate of decrease in absorbance (340 nm) is proportional to the concentration of urea in the tested sample. The concentration of urea determined by us in the control BioNorm serum (Catalog No. 1-801-0020; BioMaxima) reached 35.02 ± 1.80 mg/100 mL (mean ± SD) and was within the range of reference values provided by the manufacturer (31.0–42.0 mg/100 mL). The assay of uric acid was based on the enzymatic oxidation of this compound to allantoine and hydrogen peroxide. The generated hydrogen peroxide combines with 3,5-dichloro-2-hydroxybenzenesulfonic acid and 4-aminoantipyrine to form a colored complex whose intensity (measured at 546 nm) is proportional to the concentration of uric acid. The concentration of uric acid determined by us in the control BioNorm serum (Catalog No. 1-801-0020; BioMaxima) reached 4.23 ± 0.070 mg/100 mL (mean ± SD) and was within the range of reference values (3.82–5.16 mg/100 mL). The contents of urea and uric acid in the 24 h urine were evaluated.

#### 4.5.2. Determination of Cd Concentration in the Urine

The concentration of Cd in the urine was determined using the flameless atomic absorption spectrometry (AAS) method with electrothermal atomization in a graphite furnace with the use of a Hitachi atomic absorption spectrophotometer model Z-5000 (Tokyo, Japan), as reported [17]. Samples of 24-h urine were diluted appropriately with 0.5% nitric acid (HNO_3_) prepared via the dilution of trace-pure 65% HNO_3_ (Merck, Darmstadt, Germany) with ultra-pure water (MAXIMA purification system; ELGA, Bucks, UK). The mixture of palladium and magnesium (as nitrates; Merck, Darmstadt, Germany) was used as a matrix modifier in the AAS method. The Cd concentration was automatically read from a calibration curve prepared from a stock of standard solution (Sigma, St. Louis, MO, USA) assigned for the AAS method. The limit of Cd detection was 0.018 μg/L. To check the analytical quality of the analysis, the certified material, Trace Elements Urine level 1 (No. 201305; SeronormTM, Billingstad, Norway), was used. The Cd concentration determined by us (4.75 ± 0.31 μg/L; mean ± SD) in the certified reference urine exactly agreed with the value given by the producer (4.9 ± 0.2 μg/L). The recovery of Cd in the reference sample was 96.9%, whereas the precision of the measurement, expressed as CV, was <8.5%. Cd concentration in the urine was adjusted for creatinine concentration (determined with the use of a commercial kit No. E10051 by EMAPOL; Gdańsk, Poland).

#### 4.5.3. Histopathological Studies

Immediately after dissection and rinsing with ice-cold physiological saline, halves of the left kidneys (section in the longitudinal plane of the organ) were fixed in Bouin’s solution (prepared by mixing picric acid (Sigma-Aldrich GmbH, Steinheim, Germany), formalin (CHEMPUR, Piekary Śląskie, Poland), and acetic acid (CHEMPUR)) for 24 h, dehydrated in different concentrations of ethyl alcohol (POCh, Gliwice, Poland), cleared with xylene (POCh), and embedded in paraffin (CHEMPUR). The tissues embedded in paraffin blocks were cut on a Histocore Multicut 1860 histological microtome (Leica Biosystems, Nußloch, Germany). Tissue sections were placed on adhesive basic slides (Mar-four, Konstantynów Łódzki, Poland). Then, manual standard histological topographic staining of the preparations with hematoxylin and eosin was performed with the use of the following reagents: Harris hematoxylin (Mar-four), eosin Y (Mar-four), 99.9% ethanol (pure for analysis) (Alpinus Chemia; Solec Kujawski, Poland), xylene (pure for analysis) (Alpinus Chemia, Poland), acidic ethanol solution with 35–38% hydrochloric acid (pure for analysis) (CHEMPUR) (prepared in-house). Histological slides were also manually stained for Masson trichrome staining using a commercial kit (Catalog No. 04-010802; Bio-Optica, Milano, Italy). Histopathological evaluation and photographs were performed by a veterinary pathologist using Axiolab 5 microscopes, an Axiocam camera, and ZEN 2.0 software (Zeiss, Halle, Germany). Criteria for the histopathological assessment were based on the scientific literature and recommendations included in the International Harmonization of Nomenclature and Diagnostic Criteria (INHAND), developed and published by the Global Editorial and Steering Committee (GESC) [51]. The structure of renal tissues was evaluated using hematoxylin and eosin topographic staining, while in Masson’s staining, the basal membranes of the vessels of the glomeruli (in glomerulopathies) and renal tubules (in tubulopathies), as well as the interstitial connective tissue of the kidneys (in the processes of connective tissue hyperplasia/fibrosis), were assessed. Tissues were evaluated at ×5, ×10, and ×40 objective magnifications. The location, nature, and severity of pathological changes were assessed. Based on the literature review, the experience of the research team, and the initial review of the preparations, the catalog of histopathological changes used in the scoring (scalar) assessment was specified. The occurrence of each lesion was graded on the scale: 0—none; 1—slight; 2—moderate; and 3—severe.

#### 4.5.4. Determination of Proinflammatory Markers in the Kidney

Pre-weighted slices of the kidney (right) were homogenized using a high-performance homogenizer (Ultra-Turrax T25; IKA, Staufen, Germany) in a cold potassium phosphate buffer (50 mM, pH = 7.4; made by combining 50 mM potassium dihydrogen phosphate and 50 mM dipotassium hydrogen phosphate (POCh; Gliwice, Poland)) with the addition of butyl-hydroxytoluene (Sigma-Aldrich GmbH; Steinheim, Germany) as an antioxidant. Then, 0.01 mL of 0.5 M butyl-hydroxytoluene in acetonitrile (Merck, Darmstadt, Germany) were used per 1 mL of 10% homogenate (weight/volume—*w/v*). The homogenates were centrifuged at 10,000× *g* for 5 min at 4 °C using an MPW-350R centrifuge (Medical Instruments; Warsaw, Poland) [52].

The concentration of chemerin in the aliquots of the renal tissue homogenates was assayed with the use of the Rat Chemerin ELISA kit (Catalog No. MBS723448) by MyBioSource, Inc. (San Diego, CA, USA) based on the principle of sandwich ELISA technology. In a pre-coated plate, the assay sample and buffer were first incubated with the chemerin-HRP conjugate, followed by the substrate for the HRP enzyme in the wells. The result of the enzyme-substrate reaction was a blue complex. The reaction was stopped by adding a stop solution, causing the solution to turn yellow, and the color intensity was measured spectrophotometrically at 450 nm. The correlation between the color’s intensity and chemerin concentration is inverse. The intra- and inter-assay CVs for these measurements were <4% and 2%, respectively.

The concentrations of MIP1a and Bax in the aliquots of the kidney tissue homogenates were assessed with ELISA kits (Catalog No. SEA092Ra and No. SEB343Ra01, respectively) made by Cloud-Clone Corp. (Katy, TX, USA). Standards or samples were added to the wells with a biotin-conjugated specific antibody, and then avidin conjugated to HRP was added to each microplate well and incubated. After the addition of another substrate, the wells that contained the tested proteins changed in color. Following the cessation of the reaction, the change in color was measured spectrophotometrically (450 nm).

The intra- and inter-assay CV for MIP1a measurements were <4.4% and <7.5%, respectively, and <3% and <2% for Bax, respectively.

### 4.6. Statistical Analysis

All statistical calculations were performed using Statistica 13.3 software (StatSoft, Tulsa, OK, USA), and the data are expressed as a median, 25–75% confidence interval, and minimum and maximum. In the beginning, the normality of the distribution of the data was checked with the Shapiro-Wilk test. Because there was no normal distribution, a nonparametric signed-rank Kruskal-Wallis test with median test was performed to determine whether there were statistically significant (*p* < 0.05) differences between the six groups. If statistically significant differences were found between the six groups, multiple comparisons were carried out to examine between which two groups a statistically significant (*p* < 0.05) difference occurred. A nonparametric Friedman test was performed to test the results of measurements repeated on the same animals every other month. When the Friedman test showed statistically significant differences (*p* < 0.05) between all time points, the Wilcoxon test was performed to compare paired data.

To calculate the dependence between the values of the measured parameters, a linear regression analysis was performed. The results of this analysis are shown as the β coefficient (this metric represents the percentage of the dependent variable’s change for each unit of the independent variable’s change), R^2^ (shows the percentage of one variable that is responsible for the variability of the other), and the statistical significance (*p*). A relationship between two variables was acknowledged to be statistically significant at the value of the β coefficient for which *p* < 0.05. Furthermore, dependencies between the parameters measured in the present study and the previously published Cd concentration in the blood, urine, and kidney were analyzed ([17], Table S15).

## 5. Conclusions

Based on the results of the present study, conducted in an animal model of human lifespan environmental exposure to Cd in industrialized countries, it can be concluded that even low-level repeated intoxication with this toxic heavy metal can result in structural and functional damage to the kidney tubules and glomeruli, creating a risk of this organ injury. The finding in the used experimental model that the unfavorable impact of Cd on kidney function and structure may occur at its concentrations in the urine (0.0852–0.2820 μg/g creatinine) within the lower range of values currently found in the general population worldwide confirms that even low-level environmental exposure to this xenobiotic may pose a risk of damage to the kidney. Moreover, it can be concluded that an intake of polyphenol-rich *A. melanocarpa* berry products during low-to-moderate levels of exposure to Cd may very effectively protect from the nephrotoxic action of this heavy metal. The present study indicates that Aronia products may be an effective strategy for protecting the kidney from damage due to low-to-moderate chronic exposure to Cd.

## Figures and Tables

**Table 1:** Changes in the morphology of the kidneys of female rats in particular experimental groups.

Group and the Extent of Changes ^1^	Tubular Vacuolization	Hyalinization	Extension of the Tubular Lumen	Hyperplasia of the Epithelium of the Convoluted Tubules	Hypertrophy of the Epithelium of the Convoluted Tubules	Tubular Necrosis	Interstitial Proliferation	Congestion at the Cortex/Medullary Interface	Perivascular Oedema	Glomerulonephritis	Glomerular Congestion
% of rats with the same severity of the lesion											
Control											
0	100	100	100	100	100	100	100	100	75	50	100
1									25	50	
2											
3											
AM											
0	75	100	75	75	100	100	100	100		75	100
1	25		25	25					100	25	
2											
3											
Cd_1_											
0	75	25	50	75	75	100		25	100		100
1	25	25		25	25		25	25		25	
2			25				50	50		75	
3		50	25				25				
Cd_1_+AM											
0	100	100	100	100	100	100	75	25	75	75	100
1							25	75	25	25	
2											
3											
Cd_5_											
0	100	25	100			100		25	50		100
1				50	50				50		
2				50	50		100	25		25	
3		75						50		75	
Cd_5_+AM											
0	100	25	50	100	50	100	25	25	25	50	100
1		75	50		50		75	75	75	50	
2											
3											

The animals were treated with cadmium (Cd) in the diet at a concentration of 0, 1, or 5 mg/kg (Control, Cd_1_, and Cd_5_ groups) and/or 0.1% extract from the berries of *Aronia melanocarpa* L. (AM, Cd_1_+AM, and Cd_5_+AM groups) for 24 months. The data are shown as a percentage of four rats in each experimental group with the same severity of the lesion or its absence. ^1^ The following criteria were used to score the presence or absence of the changes and their intensity: 0—without a change; 1—a slight change; 2—a moderate change; and 3—a severe change.

**Table 2:** Relationships between the investigated biomarkers of kidney status and cadmium (Cd) concentration in the blood, urine, and kidney of female rats administered or not with a 0.1% extract from the berries of *Aronia melanocarpa* L. (AM) ^1,2,3^.

Parameter	Regression Analysis ^4^	Cd in the Blood of Rats		Cd in the Urine of Rats		Cd in the Kidney of Rats	
		Not Administered with AM ^2^	Administered with AM ^3^	Not Administered with AM	Administered with AM	Not Administered with AM	Administered with AM
KIM-1 in the Urine	β*^p^* R^2^	0.339 ^‡^ 0.105	NS	0.309 ^†^ 0.086	NS	0.509 ^‡^ 0.251	NS
β2-MG in the Urine	β*^p^* R^2^	0.425 ^‡^ 0.172	NS	0.380 ^‡^ 0.135	NS	0.638 ^‡^ 0.401	0.284 ^†^ 0.071
NAG in the Urine	β*^p^* R^2^	0.316 ^†^ 0.090	NS	NS	NS	0.434 ^‡^ 0.179	0.206 * 0.032
ALP in the Urine	β*^p^* R^2^	0.489 ^‡^ 0.231	NS	0.420 ^†^ 0.092	NS	0.553 ^‡^ 0.298	NS
ACR	β*^p^* R^2^	0.450 ^‡^ 0.194	NS	0.418 ^‡^ 0.166	NS	0.694 ^‡^ 0.476	0.224 * 0.040
PCR	β*^p^* R^2^	0.409 ^‡^ 0.159	NS	0.279 ^†^ 0.068	NS	0.624 ^‡^ 0.383	NS
Creatinine Clearance	β*^p^* R^2^	−0.232 * 0.043	NS	NS	NS	−0.414 ^‡^ 0.162	NS
Uric Acid in the Serum	β*^p^* R^2^	0.225 * 0.040	NS	0.231 * 0.043	NS	0.308^†^ 0.085	NS
Uric acid in the Urine	β*^p^* R^2^	NS	NS	NS	NS	NS	NS
Urea in the Serum	β*^p^* R^2^	0.525 ^‡^ 0.268	NS	0.372 ^‡^ 0.129	NS	0.532 ^‡^ 0.276	NS
Urea in the Urine	β*^p^* R^2^	NS	NS	NS	NS	NS	NS
Chemerin in the Kidney	β*^p^* R^2^	0.289 ^†^ 0.074	−0.498 ^‡^ 0.240	NS	−0.431 ^‡^ 0.177	NS	−0.429 ^‡^ 0.175
MIP1a in the Kidney	β*^p^* R^2^	NS	−0.315 ^†^ 0.090	NS	−0.256 * 0.056	NS	−0.444 ^‡^ 0.188
Bax in the Kidney	β*^p^* R^2^	NS	−0.394 ^‡^ 0.146	NS	−0.375 ^‡^ 0.131	NS	−0.370 ^‡^ 0.127

^1^ Cd concentrations in the blood, urine, and kidneys of the rats subjected to necropsy after 3, 10, 17, and 24 months have already been published [17]. ^2^ The results of the analysis of regression are presented as the β coefficient, R^2^, and the level of statistical significance (*p*; where * *p* < 0.05, ^†^ *p* < 0.01, and ^‡^ *p* < 0.001). NS, a lack of relationship (*p* > 0.05). ^3^ All groups not administered with AM were included in the analysis (the control group that received fodder containing 0.0584 ± 0.0049 mg Cd/kg and the Cd_1_ and Cd_5_ groups maintained on feed containing 1 and 5 mg Cd/kg, respectively). ^4^ All groups administered with AM were included in the analysis (the AM, Cd_1_+AM, and Cd_5_+AM groups). β2-MG, beta2-microglobulin; ACR, albumin concentration in the urine adjusted for creatinine concentration; ALP, alkaline phosphatase; Bax, Bcl2-associated X protein; KIM-1, kidney injury molecule 1; MIP1a, macrophage inflammatory protein 1 alpha; NAG, N-acetyl-β-D-glucosaminidase; PCR, total protein concentration in the urine adjusted for creatinine concentration.

**Table 3:** Mutual relationships between the investigated parameters describing the kidney status in the female rats administered (*italic*) or not administered with a 0.1% extract from the berries of *Aronia melanocarpa* L. (AM) ^1,2^.

Parameter	Regression Analysis ^3^	KIM 1 in the Urine	β2-MG in the Urine	NAG in the Urine	ALP in the Urine	ACR	PCR	Creatinine Clearance	Uric Acid in the Serum	Uric Acid in the Urine	Urea in the Serum	Urea in the Urine	Chemerin in the Kidney	MIP1a in the Kidney	Bax in the Kidney
β2-MG in the Urine	β*^p^* R^2^	0.447 ^‡^ 0.191	-	*0.375* ^‡^ *0.132*	*NS*	*0.543* ^‡^ *0.287*	*NS*	*NS*	*NS*	*0.300* ^†^ *0.080*	*NS*	*NS*	*NS*	*−0.420* ^‡^ *0.168*	*−0.309* ^†^ *0.086*
NAG in the Urine	β*^p^* R^2^	0.235 * 0.045	0.733 ^‡^ 0.532	-	*NS*	0.204 * *0.031*	*NS*	*NS*	*NS*	*NS*	*NS*	*NS*	*NS*	−0.250 * 0.053	*NS*
ALP in the Urine	β*^p^* R^2^	0.273 ^‡^ 0.064	0.594 ^‡^ 0.346	0.741 ^‡^ 0.544	-	*NS*	*0.269* ^†^ *0.063*	*−0.260* ^†^ *0.060*	*NS*	−0.220 * *0.039*	*NS*	*NS*	−0.203 * *0.031*	*−0.270* ^†^ *0.064*	−0.250 * *0.050*
ACR	β*^p^* R^2^	0.563 ^‡^ 0.309	0.862 ^‡^ 0.739	0.704 ^‡^ 0.490	0.548 ^‡^ 0.239	-	*NS*	*NS*	*0.312* ^†^ *0.088*	*0.341* ^‡^ *0.107*	*NS*	*NS*	*NS*	*−0.460* ^‡^ *0.207*	*−0.360* ^‡^ *0.120*
PCR	β*^p^* R^2^	0.488 ^‡^ 0.230	0.755 ^‡^ 0.566	0.777 ^‡^ 0.600	0.767 ^‡^ 0.585	0.803 ^‡^ 0.641	-	*−0.320* ^†^ *0.090*	*NS*	*NS*	*NS*	*NS*	*NS*	*−0.321* ^†^ *0.094*	*−0.320* ^†^ *0.092*
Creatinine Clearance	β*^p^* R^2^	NS	−0.418 ^‡^ 0.165	−0.310 ^†^ 0.088	−0.360 ^‡^ 0.122	−0.460 ^‡^ 0.199	−0.464 ^‡^ 0.207	-	*NS*	*NS*	*NS*	0.220 * *0.038*	*NS*	*NS*	*NS*
Uric Acid in the Serum	β*^p^* R^2^	0.345 ^‡^ 0.109	0.356 ^‡^ 0.113	0.251 * 0.053	NS	0.390 ^‡^ 0.143	NS	NS	-	*NS*	*NS*	0.217 * *0.037*	0.209 * *0.033*	*NS*	*NS*
Uric Acid in the Urine	β*^p^* R^2^	NS	NS	NS	NS	NS	NS	0.248 * 0.052	NS	-	*NS*	*0.421* *0.168*	*NS*	*NS*	*NS*
Urea in the Serum	β*^p^* R^2^	0.259 * 0.057	0.520 ^‡^ 0.262	0.591 ^‡^ 0.342	0.577 ^‡^ 0.325	0.499 ^‡^ 0.241	0.512 ^‡^ 0.254	−0.305 ^†^ 0.083	0.474 ^‡^ 0.216	NS	-	*NS*	*NS*	*NS*	*NS*
Urea in the Urine	β*^p^* R^2^	0.398 ^‡^ 0.149	0.273 ^†^ 0.065	NS	NS	0.289 ^†^ 0.073	NS	0.241 * 0.048	0.394 ^‡^ 0.146	0.628 ^‡^ 0.387	NS	-	*NS*	*NS*	*NS*
Chemerin in the Kidney	β*^p^* R^2^	NS	NS	0.360 ^‡^ 0.120	0.240 * 0.047	0.223 * 0.040	NS	0.279 ^†^ 0.068	0.232 * 0.043	NS	0.239 * 0.047	NS	-	*0.445* ^‡^ *0.189*	*0.481* ^‡^ *0.223*
MIP1a in the Kidney	β*^p^* R^2^	NS	NS	NS	NS	NS	NS	NS	NS	NS	NS	NS	0.258 * 0.057	-	*0.794* ^‡^ *0.626*
Bax in the Kidney	β*^p^* R^2^	NS	NS	NS	NS	NS	NS	NS	NS	NS	NS	0.219 * 0.037	0.273 ^†^ 0.065	0.265 ^†^ 0.060	-

^1^ The groups not administered with AM included in the analysis were the control group that received fodder containing 0.0584 ± 0.0049 mg Cd/kg, and the Cd_1_ and Cd_5_ groups maintained on feed containing 1 and 5 mg Cd/kg, respectively. ^2^ The groups administered with AM included in the analysis were the AM, Cd_1_+AM, and Cd_5_+AM groups. ^3^ The results of the analysis of regression are presented as the β coefficient, R^2^, and the level of statistical significance (*p*, where * *p* < 0.05, ^†^ *p* < 0.01, and ^‡^ *p* < 0.001). NS, a lack of relationship (*p* > 0.05). β2-MG, beta2-microglobulin; ACR, albumin concentration in the urine adjusted for creatinine concentration; ALP, alkaline phosphatase; Bax, Bcl2-associated X protein; KIM-1, kidney injury molecule 1; MIP1a, macrophage inflammatory protein 1 alpha; NAG, N-acetyl-β-D-glucosaminidase; PCR, total protein concentration in the urine adjusted for creatinine concentration.

**Table 4:** The daily intakes of cadmium (Cd) and 0.1% *Aronia melanocapra* berry extract (AM) during the 24-month experiment.

Group	Intake during the 24-Month Study ^1^	
	Cd (μg/kg b.w./24 h)	Chokeberry Extract [Polyphenols] (mg/kg b.w./24 h)
Control	2.30–4.98	0 [0]
AM	2.25–4.95	67.4–146.6 [44.3–96.4]
Cd_1_	39.2–83.8	0 [0]
Cd_1_+AM	37.5–84.9	67.2–154.7 [44.2–101.7]
Cd_5_	210.1–403.2	0 [0]
Cd_5_+AM	200.2–401.9	63.1–150.3 [41.5–98.8]

^1^ Data show the ranges of the daily intakes of Cd, chokeberry extract, and polyphenols throughout the 24-month study (the ranges represent the minimum and maximum intake for 32 rats during the first 3 months; 24 females—from the beginning of the 4th up to the end of the 10th month; 16 animals—from the beginning of the 11th month up to the end of the 17th month and then, for 8 rats, except for 7 animals in the AM, Cd_1_, and Cd_5_ groups between the 18th and 24th months; detailed data on the intakes of the extract and polyphenols in particular groups during 3, 10, 17, and 24 months have been published [17]). The intake of polyphenolic compounds was calculated assuming, according to the manufacturer, that their content in the chokeberry extract reached 65.74%. The intake of Cd in the Cd_1_ and Cd_5_ groups was calculated based on the content of this heavy metal in the Labofeed diet specified by the producer (1 or 5 mg/kg), while its intake in the control group and AM group was estimated based on its concentration in the standard diet (0.0584 ± 0.0049 mg/kg) [17].

## Data Availability

The data presented in this study are available upon request from the corresponding authors. The data are not publicly available.

## Supplementary Materials

The following supporting information can be downloaded at: https://www.mdpi.com/article/10.3390/ijms241411647/s1.

## Abbreviations

AAS method	atomic absorption spectrometry method
ACR	albumin concentration in the urine adjusted for creatinine concentration
ALP	alkaline phosphatase
AM	0.1% aqueous extract from the berries of *Aronia melanocarpa* L.
Bax	Bcl2-associated X protein
β2-MG	β2-microglobulin
b.w.	body weight
Cd	cadmium
CdCl_2_ × 2.5 H_2_O	cadmium chloride 2.5-hydrate
Cd-MT	cadmium-metallothionein complex
Cd^2+^	cadmium ions
CV	coefficient of variation
Cu	copper
ELISA	enzyme-linked immunosorbent assay
GFR	glomerular filtration rate
HNO_3_	nitric acid
HRP	horseradish peroxidase
KIM-1	kidney injury molecule-1
LOAEL	lowest observed adverse effect level
MIP1a	macrophage inflammatory protein 1 alpha
Mn	manganese
MT	metallothionein
NAC	N-acetyl-L-cysteine
NAG	N-acetyl-β-D-glucosaminidase
PCR	total protein concentration in the urine adjusted for creatinine concentration
SD	standard deviation
w.w.	wet weight
Zn	zinc
