# Supplementary material for: The Protective Potential of Aronia melanocarpa L. Berry Extract against Cadmium-Induced Kidney Damage: A Study in an Animal Model of Human Environmental Exposure to This Toxic Element
Source: Int J Mol Sci. 2023 Jul 19;24(14):11647. doi: 10.3390/ijms241411647 (PMC10381010; doi:10.3390/ijms241411647)
Supplement: Supplementary file 1 [file ijms-24-11647-s001.zip › ijms-2465278-supplementary.pdf]

Table S1. The concentration of kidney injury molecule-1 (KIM-1) and β_2-microglobulin (β2-MG) in the urine of female rats.

Group	Experiment Duration 3 Months	10 Months	17 Months	24 Months
	**KIM-1 (pg/mg Creatinine)**			
Control	96.71	90.60	179.4	185.7
	32.22 – 222.6	32.04 – 124.6	26.29 – 212.5	131.2 – 239.3
AM	171.9	97.79	254.1	260.2
	44.00 – 389.7	58.20 – 152.2	78.49 – 368.2	161.7 – 293.3
Cd_1	509.8 ^{a*}	240.8 ^{a*}	439.3 ^{a†}	360.5
	200.8 - 1754	149.0 – 419.2	273.6 – 549.9	279.0 – 752.6
Cd_1+AM	69.65 ^{c*}	84.64 ^{c*}	149.7 ^{c†}	159.7 ^{c†}
	9.144 – 327.2	54.10 – 138.3	107.6 – 222.3	101.9 – 197.2
Cd_5	544.0 $^{a*\ d*}$	876.8 $^{a\ddagger\ b\ddagger\ d\ddagger}$	463.3 $^{a\ddagger\ d\ddagger}$	418.7 $^{a*\ d\ddagger}$
	190.4 – 834.1	569.2 - 1441	330.8 – 1050.8	363.4 – 525.5
Cd_5+AM	55.39 $^{c\ddagger\ e\ddagger}$	109.9 ^{e*}	229.2 ^{e*}	141.4 $^{c\dagger\ e\ddagger}$
	25.19 – 143.2	101.1 – 157.7	106.8 – 300.5	109.4 – 176.9
	**β2-MG (ng/mg Creatinine)**			
Control	2.114	4.803	4.381	4.688
	1.353 – 5.483	0.543 – 6.743	0.740 – 5.816	2.982 – 5.715
AM	2.131	2.663	4.494	4.965
	1.590 – 3.671	1.486 – 4.713	1.958 – 6.402	3.521 – 5.943
Cd_1	2.780	4.545	7.119 $^{a*\ b*}$	10.24 $^{a\dagger\ b*}$
	1.495 – 5.356	2.487 – 7.624	5.876 – 13.95	7.430 – 20.92
Cd_1+AM	3.480	4.309	4.561 ^{c*}	6.145 ^{c*}
	1.121 – 5.045	1.038 – 6.561	2.806 – 6.002	2.164 – 8.531
Cd_5	2.661	5.557	11.23 $^{a\ddagger\ b\ddagger\ d\dagger}$	16.94 $^{a\ddagger\ b\dagger\ d\dagger}$
	2.051 – 5.192	2.012 – 12.84	10.02 – 20.14	11.40 – 23.44
Cd_5+AM	2.580	2.542	4.917 ^{e†}	5.044 $^{c*\ e*}$
	1.702 – 2.929	1.196 – 8.299	2.848 – 7.005	2.209 – 6.278

The animals were treated with cadmium (Cd) in the diet at the concentration of 0, 1, or 5 mg/kg (Control, Cd_1, and Cd_5 groups) and/or 0.1% extract from the berries of *Aronia melanocarpa* L. (AM, Cd_1+AM, and Cd_5+AM groups) for 3, 10, 17, and 24 months. Data are shown as a median and minimum and maximum values for eight animals (except for seven females in the AM, Cd_1, and Cd_5 groups after 24 months). Statistically significant differences (Kruskal–Wallis test) compared to: a—Control group; b—AM group; c—Cd_1 group; d—Cd_1+AM group, and e—Cd_5 group, where * $p < 0.05$, † $p < 0.01$, and ‡ $p < 0.001$, are marked.

Table S2. The concentration of β2-microglobulin (β2-MG) in the urine of female rats evaluated every other month during the 24-month study.
The animals were treated with cadmium (Cd) in the diet at the concentration of 0, 1, or 5 mg/kg (Control, Cd_1, and Cd_5 groups) and/or 0.1% extract from the berries of *Aronia melanocarpa* L. (AM,

**Months**	**Control**	**AM**	**Cd_1**	**Cd_1+AM**	**Cd_5**	**Cd_5+AM**
0	2.368	2.319	2.482	2.261	2.221	2.241
	1.606 – 3.064	1.227 – 3.194	1.660 – 3.419	1.736 – 2.964	1.608 – 2.921	1.637 – 2.861
2	3.557	2.862	3.148	3.567	3.886	2.995
	0.076 – 5.407	0.592 – 7.981	2.063 – 5.151	1.338 – 3.968	1.040 – 4.431	1.741 – 4.676
4	2.963	2.006	2.423	2.380	2.123	2.904
	1.180 – 4.587	1.197 – 3.330	1.088 – 5.335	1.657 – 4.680	0.484 – 6.224	0.052 – 8.989
6	3.801	4.025	5.357	2.185 $^{b\dagger\ c\ddagger}$	3.422	2.462 ^{c†}
	2.607 – 6.724	2.932 – 5.064	3.743 – 8.691	0.795 – 2.392	1.908 – 4.717	1.141 – 4.894
8	2.967	2.811	5.071	2.403 ^{c†}	5.095 $^{b*\ d\dagger}$	2.009 $^{c\ddagger\ e\ddagger}$
	2.171 – 4.023	1.106 – 4.319	3.948 – 5.886	1.322 – 3.717	4.085 – 6.042	1.433 – 2.368
10	3.889	2.930	7.402 ^{b†}	4.740	6.113	2.798 ^{c*}
	2.597 – 4.394	1.635 – 5.185	4.402 – 8.386	1.142 – 7.218	3.213 – 8.032	1.316 – 6.469
12	3.970	3.474	8.368 ^{b†}	3.242 ^{c†}	8.842 $^{a*\ b\dagger\ d\dagger}$	3.499 $^{c\dagger\ e\dagger}$
	3.288 – 4.802	1.708 – 5.445	7.059 – 11.98	2.621 – 7.370	6.893 – 12.42	1.176 – 5.669
14	4.321	5.047	8.500 ^{a*}	4.794	8.722 ^{a*}	3.508 $^{c\dagger\ e\ddagger}$
	2.106 – 6.111	2.446 – 7.757	5.665 – 13.40	3.870 – 7.917	6.301 – 10.72	1.687 – 5.491
16	3.983	4.085	7.992 $^{a\dagger\ b*}$	4.677 ^{c*}	11.07 $^{a\ddagger\ b\dagger\ d\dagger}$	4.907 ^{e*}
	1.673 – 5.211	1.780 – 5.820	6.389 – 15.41	2.551 – 6.252	9.514 – 20.22	5.879 – 2.589
18	4.802	5.142	12.39 $^{a\ddagger\ b\dagger}$	8.128 ^{c*}	11.17 $^{a\dagger\ b*}$	6.426 $^{c\dagger\ e*}$
	4.128 – 10.11	4.539 – 5.846	10.50 – 20.66	4.518 – 9.939	9.380 – 19.75	5.195 – 7.215
20	5.339	5.318	10.42 ^{a*}	4.099 ^{c†}	10.81 ^{a*}	5.029 $^{e\dagger\ c*}$
	3.308 – 7.994	3.893 – 7.017	8.405 – 17.71	2.696 – 7.795	8.956 – 15.18	3.372 – 6.585
22	5.936	5.378	17.01 ^{a†}	6.033 ^{c†}	16.43 $^{a\dagger\ d\dagger}$	7.106 ^{e*}
	4.162 – 7.605	3.808 – 6.788	10.11 – 21.99	3.961 – 8.432	11.36 – 31.46	5.040 – 9.443
24	4.688	4.965	10.24 $^{a\dagger\ b*}$	6.145 ^{c*}	16.94 $^{a\ddagger\ b\dagger\ d\dagger}$	5.044 $^{c*\ e*}$
	2.982 – 5.715	3.521 – 5.943	7.430 – 20.92	2.164 – 8.531	11.40 – 23.44	2.209 – 6.278
Friedman Test	$p < 0.001$	$p < 0.001$	$p < 0.001$	$p < 0.001$	$p < 0.001$	$p < 0.001$
Wilcoxon Test	0 – 6, 10…24 *<br>2 – 22 *<br>4 – 12, 18…24 *<br>6 – 18, 22 *<br>8 – 10, 12, 18…24 *<br>10 – 18…22 *<br>12 – 18…22 *<br>16 – 20, 22 *<br>22 – 24 *	0 – 6, 12…24 *<br>4 – 6, 10…24 *<br>6 – 10, 18…22 *<br>8 – 14, 18…24 *<br>10 – 18…24 *<br>12 – 14, 18…24 *<br>16 – 20, 22 *	0 – 6…24 *<br>2 – 6…24 *<br>4 – 6…24 *<br>6 – 12, 16…24 *<br>8 – 12…24 *<br>10 – 12 *<br>10 – 18…24 *<br>12 – 18…24 *<br>14 – 18, 22 *<br>16 – 18, 22 *<br>20 – 22<br>20 – 22 *	0 – 2, 10…24 *<br>2 – 6, 14…18, 22, 24 *<br>4 – 14… 24 *<br>6 – 10…24 †<br>8 – 10 *<br>8 – 12…24 †<br>10 – 18 *<br>12 – 18 †<br>12 – 22 *<br>14 – 18 *<br>16 – 18, 22 †<br>18 – 20 †<br>20 – 22 †	0 – 2, 6 *<br>0 – 8…24 †<br>2 – 8, 12…24 †<br>2 – 10 *<br>4 – 8, 10 *<br>4 – 12…24 †<br>6 – 10 *<br>6 – 8, 12…24 †<br>8 – 12…24 †<br>10 – 12, 16…24 †<br>10 – 14 *<br>12 – 16, 22, 24 *<br>14 – 16, 22, 24 †<br>14 – 18, 20 *<br>16 – 24 *<br>18 – 22, 24 *<br>20 – 22, 24 *	0 – 2, 12, 16 *<br>0 – 16…24 †<br>2 – 8, 20, 24 *<br>2 – 18, 22 †<br>6 – 16, 20, 24 *<br>6 – 18, 22 †<br>8 – 10, 12, 14 *<br>8 – 16…24 †<br>10 – 18 †<br>10 -22 *<br>12 – 18, 22 †<br>12 – 20, 24 *<br>14 – 16, 20 *<br>14 – 18, 22 †<br>16 – 18, 22 †<br>18 – 24 *<br>20 – 22 †<br>22 – 24*

Cd_1+AM, and Cd_5+AM groups) for 24 months. Data are shown as a median and minimum and maximum values for eight animals (except for seven females in the AE, Cd_1, and Cd_5 groups after 20, 22, and 24 months). Statistically significant differences (Kruskal–Wallis test) compared to: a—Control group; b—AM group; c—Cd_1 group; d—Cd_1+AM group; and e—Cd_5 group. Wilcoxon test was performed to compare data between particular two time points in the same animals during the 24-month study. * $p < 0.05$, † $p < 0.01$, and ‡ $p < 0.001$.

Table S3. The activities of N-acetyl-β-D-glucosaminidase (NAG) and alkaline phosphatase (ALP) in the urine of female rats.

**Group**	**Experiment Duration** **3 Months**	**10 Months**	**17 Months**	**24 Months**
		**NAG (U/L)**		
Control	85.25	126.6	113.9	137.6
	60.38 – 114.1	68.55 – 166.7	87.03 – 156.6	68.78 – 154.8
AM	95.34	111.0	123.9	116.6
	63.27 – 116.6	61.20 – 145.4	77.49 – 137.1	76.75 – 160.8
Cd$_1$	104.5	636.0 ^{b‡}	520.09 $^{a\dagger\ b*}$	3056 $^{a*\ b*}$
	53.87 – 132.5	380.7 – 801.9	387.9 – 776.9	2971 – 4112
Cd$_1$+AM	93.65	141.7	121.7 ^{c†}	117.1 ^{c†}
	67.78 – 116.0	92.99 – 230.1	90.41 – 162.7	93.61 – 141.5
Cd$_5$	93.46	800.4 $^{a\dagger\ b\ddagger\ d*}$	596.9 $^{a\dagger\ b*\ d\dagger}$	3453 $^{a*\ b\dagger\ d\dagger}$
	75.54 – 120.8	347.7 – 935.5	496.0 – 769.4	2999 – 4836
Cd$_5$+AM	92.83	108.1 $^{c\dagger\ e\ddagger}$	129.7 ^{c*}	131.9 ^{e†}
	41.47 – 116.5	46.10 – 173.3	94.98 – 152.2	96.65 – 148.9
		**ALP (U/L)**		
Control	39.22	42.45	36.82	38.08
	27.41 – 68.93	10.90 – 70.19	30.52 – 71.00	24.11 – 113.0
AM	27.16	36.70	37.81	34.00
	16.80 – 57.10	24.81 – 79.00	35.84 – 41.36	28.71 -102.0
Cd$_1$	31.99	146.9 $^{a\dagger\ b\dagger}$	96.05 $^{a\dagger\ b\dagger}$	149.9 ^{a*}
	23.80 – 69.30	99.75 – 192.0	69.84 – 139.7	105.7 – 208.8
Cd$_1$+AM	32.08	60.66	51.46	49.60
	27.57 – 35.84	31.46 - 79.95	43.75 – 59.18	26.10 – 120.0
Cd$_5$	35.84	185.1 $^{a\ddagger\ b\ddagger\ d*}$	111.7 $^{a\dagger\ b\dagger}$	278.0 $^{a\ddagger\ b\dagger\ d\dagger}$
	27.57 – 71.50	90.98 – 303.3	89.60 – 127.6	181.6 – 375.2
Cd$_5$+AM	33.36	47.60 ^{e†}	36.76 $^{c\ddagger\ e\ddagger}$	39.06 ^{e†}
	13.51 – 56.90	25.38 – 102.0	34.92 – 37.68	22.10 – 70.36

The animals were treated with cadmium (Cd) in the diet at the concentration of 0, 1, or 5 mg/kg (Control, Cd$_1$, and Cd$_5$ groups) and/or 0.1% extract from the berries of *Aronia melanocarpa* L. (AM, Cd$_1$+AM, and Cd$_5$+AM groups) for 3, 10, 17, and 24 months. Data are shown as a median and minimum and maximum values for eight animals (except for seven females in the AM, Cd$_1$, and Cd$_5$ groups after 24 months). Statistically significant differences (Kruskal–Wallis test) compared to: a—Control group; b—AM group; c—Cd$_1$ group; d—Cd$_1$+AM group; and e—Cd$_5$ group, where * $p < 0.05$, † $p < 0.01$, and ‡ $p < 0.001$, are marked.

Table S4. The activity of N-acetyl-β-D-glucosaminidase (NAG) in the urine of female rats evaluated every other month during the 24-month study.

Months	Control	AM	Cd_1	Cd_1+AM	Cd_5	Cd_5+AM
0	16.75<br>8.988 – 19.91	16.75<br>9.913 – 24.13	17.14<br>14.31 – 21.34	16.68<br>11.99 – 19.90	16.43<br>7.280 – 19.91	16.75<br>9.781 – 20.14
2	78.08<br>22.65 – 104.9	89.21<br>47.80 – 103.3	61.01<br>14.57 – 85.23	69.08<br>42.79 – 85.69	72.73<br>65.20 – 93.12	39.17<br>27.11 – 71.12
4	95.50<br>7.445 – 151,9	74.08<br>3.537 – 169.0	108.0<br>92.17 – 143.5	106.4<br>49.72 – 169.1	106.5<br>36.47 – 180.6	86.92<br>30.03 – 132.6
6	115.3<br>89.10 – 123.9	63.15<br>31.55 – 120.5	117.1<br>72.58 – 150.7	114.5<br>62.79 – 128.8	124.6<br>84.56 – 144.5	110.0<br>75.24 – 133.7
8	64.63<br>11.74 – 150.8	36.21<br>10.57 – 103.3	116.6 ^{b†}<br>89.28 – 189.4	92.63<br>22.08 – 193.4	92.13<br>73.36 – 128.4	105.4<br>29.44 – 142.6
10	106.6<br>88.43 – 198.2	69.69<br>39.73 – 121.4	617.1 ^{b‡}<br>342.5 – 890.7	123.3<br>49.66 – 287.0	710.4 $^{a* b\ddagger d*}$<br>312.8 – 841.6	81.04 $^{c\dagger e\ddagger}$<br>34.56 – 261.3
12	127.6<br>63.12 – 173.4	111.5<br>52.14 – 133.3	391.0 $^{a\dagger b\dagger}$<br>345.1 – 479.5	65.10 ^{c‡}<br>46.72 – 99.45	406.4 $^{a\dagger b\dagger d\ddagger}$<br>354.6 – 521.3	112.3 ^{e*}<br>93.00 – 143.5
14	101.8<br>66.93 – 128.4	86.71<br>53.61 – 148.7	406.7 $^{a* b*}$<br>321.1 – 487.0	72.89 ^{c‡}<br>44.41 – 85.14	434.4 $^{a* b\dagger d\ddagger}$<br>397.2 – 553.5	97.64 ^{e*}<br>74.00 – 111.5
16	104.6<br>69.24 – 127.0	78.09<br>50.17 – 138.1	490.0 $^{a* b\ddagger}$<br>371.7 – 745.5	99.79 ^{c*}<br>58.30 – 117.3	572.7 $^{a\dagger b\ddagger d\dagger}$<br>475.9 – 738.2	94.84 ^{e†}<br>90.20 – 125.4
18	107.3<br>59.32 – 141.0	134.1<br>107.6 – 195.2	507.1 ^{a‡}<br>341.7 – 579.6	126.1 ^{c*}<br>90.37 – 159.4	477.9 $^{a\ddagger d\dagger}$<br>441.9 – 578.8	126.9 $^{c* e\dagger}$<br>93.73 – 180.2
20	105.5<br>79.10 – 127.1	122.7<br>78.80 – 139.7	484.4 ^{a*}<br>403.1 – 548.8	74.04 ^{c‡}<br>61.08 – 124.8	542.2 $^{a\dagger d\ddagger}$<br>420.9 – 570.5	97.98 $^{c* e\dagger}$<br>92.13 – 133.7
22	127.5<br>118.4 – 171.9	129.9<br>107.7 – 192.3	2319 $^{a* b*}$<br>1990 – 3188	132.4 ^{c*}<br>97.44 – 150.7	3132 $^{a\dagger b\dagger d\dagger}$<br>2727 - 4396	156.4 ^{e*}<br>122.5 – 210.8
24	137.6<br>68.78 – 154.8	116.6<br>76.75 – 160.8	3056 $^{a* b*}$<br>2971 – 4112	117.1 ^{c*}<br>93.61 – 141.5	3453 $^{a* b\dagger d\dagger}$<br>2999 – 4836	131.9 ^{e†}<br>96.65 – 148.9
Friedman Test	$p < 0.001$	$p < 0.001$	$p < 0.001$	$p < 0.001$	$p < 0.001$	$p < 0.001$
Wilcoxon Test	0 – 2…24 † 2 – 6, 10, 22 † 2 – 12, 16, 20, 24 * 6 – 22 † 8 – 22 * 14 – 22 * 16 – 22 † 16 – 24 * 18 – 22 † 20 – 22 *	0 – 2, 6, 10…18 † 0 – 4, 8, 20…24 * 2 – 18 † 2 – 20…24 * 4 – 18, 22 * 6 – 18 † 6 – 20, 22 * 8 – 12, 16, 20…24 * 8 – 18 † 10 – 18 † 10 – 20…24 * 12 – 18 * 14 – 18 * 16 – 18…24 *	0 – 2, 20…24 * 0 – 4…18 † 2 – 4…18 † 2 – 20…24 * 4 – 10…18 † 4 – 20…24 * 6 – 10…18 † 6 – 20…24 * 8 – 10…18 † 8 – 20…24 * 10 – 12, 14, 18, 22, 24* 12 – 16…24 * 14 – 20…24 * 16 – 22, 24 * 18 – 22, 24 * 20 – 22, 24 * 22 – 24 *	0 – 2…24 † 2 – 4…10, 16, 20 * 2 – 18, 22, 24 † 4 – 12, 14 † 6 – 12, 14 † 6 – 20 * 10 – 12 † 10 – 14 * 12 – 16, 18, 22, 24 † 14 – 16 * 14 – 18, 22, 24 † 16 – 18, 24 * 16 – 22 † 18 – 20 * 20 – 22 † 20 – 24 *	0 – 2…18 † 0 – 20…24 * 2 – 4, 20…24 * 2 – 6…18 † 4 – 10…18 † 4 – 20…24 * 6 – 10…18 † 6 – 20…24 * 8 – 10…18 † 8 – 20…24 * 10 – 12, 14, 18…24 * 12 – 16, 22, 24 * 14 – 16, 22, 24 * 16 – 22, 24 * 18 – 22, 24 * 20 – 22, 24 * 22 – 24 *	0 – 2…24 † 2 – 4, 8, 10 * 2 – 6, 12…24 † 4 – 12, 18, 22 † 4 - 16, 20, 24 * 6 – 22 † 8 – 22 † 12 – 14, 16 * 12 – 22 † 14 – 18, 24 * 14 – 22 † 16 – 18, 24 * 16 – 22 † 18 – 22 * 20 – 22 † 20 – 24 * 22 – 24*

The animals were treated with cadmium (Cd) in the diet at the concentration of 0, 1, or 5 mg/kg (Control, Cd_1, and Cd_5 groups) and/or 0.1% extract from the berries of *Aronia melanocarpa* L. (AM, Cd_1+AM, and Cd_5+AM groups) for 24 months. Data are shown as a median and minimum and maximum values for eight animals (except for seven females in the AE, Cd_1, and Cd_5 groups after 20, 22, and 24 months). Statistically significant differences (Kruskal–Wallis test) compared to: a—Control group; b—AM group; c—Cd_1 group; d—Cd_1+AM group; and e—Cd_5 group. Wilcoxon test was performed to compare data between particular two time points in the same animals during the 24-month study. * $p < 0.05$, † $p < 0.01$, and ‡ $p < 0.001$.

Table S5. The activity of alkaline phosphatase (ALP) in the urine of female rats evaluated every other month during the 24-month study.

Months	Control	AM	Cd$_1$	Cd$_1$+AM	Cd$_5$	Cd$_5$+AM
0	24.34	22.96	23.64	22.06	24.34	24.34
	15.93 – 34.90	15.14 – 34.90	13.59 – 33.08	19.53 – 33.84	11.03 – 30.33	19.53 – 33.94
2	24.42	27.57	22.06	13.39	38.59	33.08
	22.06 – 33.08	22.06 – 55.14	11.03 – 33.08	11.03 – 22.06	21.31 – 88.22	22.48 – 55.14
4	26.77	29.97	40.00	42.36	128.2 $^{a* \, b*}$	55.14
	19.30 – 46.87	15.16 – 49.63	30.33 – 52.38	11.03 – 68.93	38.59 – 140.6	5.514 – 204
6	27.57	31.25	59.67	39.21	119.5 $^{a\ddagger \, b\ddagger}$	79.55 ^{a*b*}
	22.06 – 38.60	24.81 – 43.25	27.57 – 132.3	32.45 – 82.71	66.17 – 187.5	41.36 – 173.7
8	39.61	41.36	73.80	55.14	125.3 ^{a*}	27.57 ^{e†}
	13.79 – 81.96	27.57 – 82.71	44.11 – 121.3	13.78 – 132.4	55.14 – 198.5	16.54 – 82.71
10	40.38	37.22	114.2 $^{a* \, b\dagger}$	54.22	178.5 $^{a\ddagger \, b\ddagger \, d*}$	48.77 ^{e†}
	13.25 – 56.97	24.81 – 42.42	65.10 – 165.0	34.16 – 77.20	129.0 – 196.0	28.53 – 92.00
12	28.27	27.57	97.13 $^{a* \, b*}$	44.72	117.2 $^{a\dagger \, b\dagger}$	34.46 $^{c* \, e\dagger}$
	24.81 – 60.65	18.55 – 58.47	42.81 – 139.9	24.81 – 65.42	77.19 – 159.9	5.514 – 57.89
14	32.43	36.18	89.76 ^{a*}	42.79	104.5 $^{a\dagger \, b*}$	25.23 $^{c\dagger \, e\ddagger}$
	13.78 – 68.93	23.78 – 52.38	44.11 – 170.9	27.57 – 79.95	86.66 – 130.0	19.21 – 44.22
16	33.32	31.89	85.75 $^{a* \, b*}$	39.18	121.0 $^{a\dagger \, b\dagger \, d*}$	30.63 $^{c\ddagger \, e\ddagger}$
	29.87 – 59.17	29.87 – 36.33	58.20 – 116.5	29.10 – 60.33	101.1 – 125.6	29.10 – 31.40
18	36.53	22.52	95.12 $^{a* \, b*}$	38.60	117.2 $^{a\dagger \, b\dagger}$	17.92 $^{c\ddagger \, e\ddagger}$
	21.14 – 44.06	20.22 – 45.03	50.55 – 133.3	25.73 – 42.81	96.50 – 160.8	8.271 – 20.72
20	38.14	34.00	95.58 ^{a*}	43.19	108.2 ^{a†}	33.84 $^{c\dagger \, e\ddagger}$
	31.25 – 51.46	22.06 – 61.57	70.76 – 136.8	21.14 – 66.17	84.03 – 119.7	20.22 – 44.11
22	35.54	30.65	91.29 ^{a*}	44.11	123.0 $^{a\dagger \, b\dagger \, d\dagger}$	35.03 ^{e†}
	26.65 – 44.06	22.98 – 55.14	61.57 – 123.2	20.22 – 71.16	92.41 – 124.8	22.06 – 77.20
24	38.08	34.00	149.9 ^{a*}	49.60	278.0 $^{a\ddagger \, b\dagger \, d\dagger}$	39.06 ^{e†}
	24.11 – 113.0	28.71 – 102.0	105.7 – 208.8	26.10 – 120.0	181.6 – 375.2	22.10 – 70.36
Friedman Test	$p < 0.001$	$p = 0.20$	$p < 0.001$	$p < 0.001$	$p < 0.001$	$p < 0.001$
Wilcoxon Test	0 – 10, 12, 22, 24 *<br>0 – 16, 20 †<br>2 – 10, 16, 20 *<br>2 – 22 †<br>6 – 10, 20 *		0 – 4, 8…18 †<br>0 – 6, 20…24 *<br>2 – 4, 8…18 †<br>2 – 6, 20…24 *<br>4 – 8, 12, 20…24 *<br>4 – 10, 14…18 †<br>6 – 24 *<br>8 – 10, 24 *<br>10 – 16 *<br>12 – 24 *<br>14 – 24 *<br>16 – 24 *<br>18 – 24 *<br>22 – 24 *	0 – 2, 8, 12, 16…22 *<br>0 – 6, 10, 14, 24 †<br>2 – 4, 20 *<br>2 – 6…18, 22, 24 †<br>8 – 22 *<br>10 – 18 *	0 – 4…18 †<br>0 – 20…24 *<br>2 – 4, 20…24 *<br>2 – 6…18 †<br>4 – 10, 24 *<br>6 – 24 *<br>8 – 24 *<br>10 – 12, 18…24 *<br>10 – 14, 16 †<br>12 – 24 *<br>14 – 24 †<br>16 – 24 *<br>18 - 24 *<br>20 – 24 *<br>20 – 24 *	0 – 2, 16, 20, 24*<br>0 – 6, 10, 18 †<br>2 – 6 *<br>2 – 18 †<br>6 – 12, 20…24 *<br>6 – 14…18 †<br>8 – 18 *<br>10 – 14, 16 *<br>10 – 18 †<br>14 – 18 †<br>14 – 24 *<br>16 – 18 †<br>18 – 20…24 †

The animals were treated with cadmium (Cd) in the diet at the concentration of 0, 1, or 5 mg/kg (Control, Cd$_1$, and Cd$_5$ groups) and/or 0.1% extract from the berries of *Aronia melanocarpa* L. (AM, Cd$_1$+AM, and Cd$_5$+AM groups) for 24 months. Data are shown as a median and minimum and maximum values for eight animals (except for seven females in the AE, Cd$_1$, and Cd$_5$ groups after 20, 22, and 24 months). Statistically significant differences (Kruskal–Wallis test) compared to: a—Control group; b—AM group; c—Cd$_1$ group; d—Cd$_1$+AM group; and e—Cd$_5$ group. Wilcoxon test was performed to compare data between particular two time points in the same animals during the 24-month study. * $p < 0.05$, † $p < 0.01$, and ‡ $p < 0.001$.

Table S6. The concentrations of albumin (ACR) and total protein (PCR) adjusted for creatinine concentration in the urine of female rats.

**Group**	**Experiment Duration**			
	**3 Months**	**10 Months**	**17 Months**	**24 Months**
		**ACR (mg/mg Creatinine)**		
Control	6.369	8.282	8.837	9.537
	3.541 – 8.987	6.359 – 13.17	7.225 – 9.645	7.461 – 11.32
AM	6.658	8.888	7.668	9.006
	4.004 – 9.908	5.702 – 10.91	4.220 – 10.43	7.223 – 12.73
Cd_1	8.167	10.04	13.48 $^{a* \; b*}$	21.34 ^{a*}
	5.491 – 10.87	6.501 – 14.56	11.14 – 22.13	17.06 – 26.69
Cd_1+AM	7.732	9.038	13.94 $^{a* \; b*}$	12.18 ^{c*}
	6.244 – 8.377	2.602 – 11.75	13.24 – 20.07	5.142 – 16.59
Cd_5	9.400	9.500	22.45 $^{a‡ \; b‡}$	22.56 $^{a† \; d*}$
	6.156 – 11.13	6.810 – 17.24	20.65 23.80	20.95 – 25.47
Cd_5+AM	7.355	5.716 $^{c† \; d* \; e†}$	11.79 ^{e*}	11.45 $^{c‡ \; e†}$
	4.764 – 9.541	3.375 – 7.050	9.600 – 13.74	6.473 – 13.38
		**PCR (mg/mg Creatinine)**		
Control	6.194	11.12	12.27	11.29
	3.634 – 7.743	7.831 – 14.85	9.063 – 17.44	8.320 – 12.56
AM	7.494	9.020	9.230	11.68
	2.978 – 13.09	6.737 – 13.96	8.375 – 23.80	10.36 – 20.00
Cd_1	7.329	38.00 $^{a* \; b†}$	38.10 $^{a* \; b†}$	64.28 ^{a*}
	3.473 – 12.99	27.61 – 41.28	33.20 – 61.49	56.08 – 82.50
Cd_1+AM	8.175	9.500 ^{c*}	12.01 ^{c*}	8.986 ^{c‡}
	3.454 – 14.75	6.974 – 15.08	7.137 – 17.40	4.179 – 13.28
Cd_5	6.218	42.14 $^{a† \; b‡ \; d‡}$	42.06 $^{a* \; b† \; d†}$	63.09 $^{a* \; d‡}$
	3.893 – 8.271	29.20 – 55.61	25.51 – 79.63	55.44 – 79.04
Cd_5+AM	8.289	12.40 ^{e*}	11.19 $^{c† \; e†}$	10.71 ^{e†}
	6.380 – 12.31	11.35 – 14.24	8.120 – 13.98	8.080 – 14.72

The animals were treated with cadmium (Cd) in the diet at the concentration of 0, 1, or 5 mg/kg (Control, Cd_1, and Cd_5 groups) and/or 0.1% extract from the berries of *Aronia melanocarpa* L. (AM, Cd_1+AM, and Cd_5+AM groups) for 3, 10, 17, and 24 months. Data are shown as a median and minimum and maximum values for eight animals (except for seven females in the AM, Cd_1, and Cd_5 groups after 24 months). Statistically significant differences (Kruskal–Wallis test) compared to: a—Control group; b—AM group; c—Cd_1 group; d—Cd_1+AM group; and e—Cd_5 group, where * $p < 0.05$, † $p < 0.01$, and ‡ $p < 0.001$, are marked.

Table S7. The concentration of albumin in the urine adjusted for creatinine concentration (ACR) of female rats evaluated every other month during the 24-month study.

Months	Control	AM	Cd$_1$	Cd$_1$+AM	Cd$_5$	Cd$_5$+AM
0	1.690<br>1.152 – 2.939	1.677<br>1.025 – 2.316	1.539<br>0.986 – 2.940	1.740<br>0.982 – 2.316	1.543<br>0.931 – 2.155	1.412<br>0.952 – 2.180
2	6.186<br>3.591 – 7.423	6.156<br>4.166 – 9.156	6.475<br>3.630 – 9.276	6.623<br>3.926 – 8.001	6.818<br>5.477 – 9.266	6.578<br>3.824 – 8.632
4	6.590<br>4.300 – 8 833	7.872<br>3.551 – 8.703	6.367<br>3.834 – 9.528	3.991<br>2.715 – 11.20	11.32<br>6.790 – 15.62	6.754<br>3.761 – 9.593
6	6.745<br>5.486 – 8.882	7.616<br>5.491 – 8.236	14.06 ^{a*}<br>10.33 – 14.91	5.647 ^{c‡}<br>4.516 – 6.288	10.08 ^{d†}<br>8.112 – 11.41	5.618 $^{c\ddagger\ e\dagger}$<br>3.950 – 7.141
8	8.025<br>4.945 – 13.68	7.013<br>4.122 – 9.978	15.95 ^{b†}<br>8.400 – 24.11	9.037<br>6.491 – 15.03	13.76 ^{b†}<br>10.07 – 16.75	7.073 $^{c\dagger\ e\dagger}$<br>5.664 – 8.014
10	7.529<br>5.781 – 11.98	8.080<br>5.183 – 9.920	10.54<br>6.826 – 15.29	9.490<br>2.732 – 12.34	9.974<br>7.151 – 18.10	6.001 $^{c\dagger\ d*\ e\dagger}$<br>3.544 – 7.403
12	9.350<br>5.614 – 14.70	6.675<br>4.185 – 9.656	10.20<br>5.433 – 16.38	11.65 ^{b*}<br>9.215 – 14.58	13.38 ^{b†}<br>9.778 – 17.77	7.914 ^{a*}<br>5.138 – 11.54
14	11.81<br>8.065 – 17.17	8.319<br>6.114 – 12.50	11.75<br>6.720 – 15.86	12.80 ^{b*}<br>11.30 – 16.21	14.68 ^{b*}<br>8.864 – 25.45	9.824 ^{e*}<br>6.527 – 11.93
16	9.126<br>3.253 – 11.46	8.408<br>4.773 – 17.37	12.67<br>7.988 – 24.71	11.14<br>6.519 – 23.65	20.70 $^{a\ddagger\ b\dagger}$<br>16.73 – 23.64	9.534 ^{e†}<br>4.875 – 13.17
18	8.345<br>4.715 – 13.55	9.085<br>6.327 – 15.42	14.44 $^{a\dagger\ b*}$<br>12.31 – 21.86	9.461 ^{c*}<br>5.692 – 12.60	16.18 $^{a\dagger\ b*\ d*}$<br>13.55 – 20.21	9.313 ^{e*}<br>7.799 – 13.72
20	9.239<br>5.389 – 13.16	9.781<br>3.432 – 20.66	18.86 ^{a*}<br>14.37 – 20.77	10.10 ^{c*}<br>8.035 – 20.38	22.45 $^{a\dagger\ b*}$<br>16.84 – 25.87	11.40 ^{e*}<br>11.07 – 17.16
22	9.626<br>7.147 – 11.48	9.315<br>6.073 – 15.13	22.77 $^{a\ddagger\ b\dagger}$<br>16.10 – 25.90	11.96 ^{c*}<br>10.68 – 15.92	22.72 $^{a\ddagger\ b\dagger}$<br>17.39 – 28.61	10.62 $^{c*\ e*}$<br>9.134 – 17.61
24	9.537<br>7.461 – 11.32	9.006<br>7.223 – 12.73	21.34 ^{a*}<br>17.06 – 26.69	12.81 ^{c*}<br>5.142 – 16.59	22.56 $^{a\dagger\ d*}$<br>20.95 – 25.47	11.45 $^{a\ddagger\ e\dagger}$<br>6.473 – 13.38
Friedman Test	$p < 0.001$	$p < 0.001$	$p < 0.001$	$p < 0.001$	$p < 0.001$	$p < 0.001$
Wilcoxon Test	0 – 2…24 †<br>2 – 6…12, 16, 22 *<br>2 – 14, 18, 24 †<br>4 – 14, 22 *<br>4 – 24 †<br>6 – 14, 22, 24 *<br>10 – 14 *<br>12 – 14 *<br>14 – 16 *	0 – 2…18 †<br>0 – 20…24 *<br>2 – 16, 20, 22 *<br>2 – 18 †<br>4 – 24 *<br>6 - 22, 24 *<br>8 – 22, 24 *<br>10 – 22, 24 *<br>12 – 14, 22, 24 *	0 – 2…18 †<br>0 – 20…24 *<br>2 – 6, 8, 14…18 †<br>2 – 10, 20…24 *<br>4 – 6, 16, 18 †<br>4 – 8, 10, 14, 20…24 *<br>6 – 20…24 *<br>8 – 12 †<br>10 – 20…24 *<br>12 – 18…24 *<br>14 – 18…24 *<br>16 – 20…24 *<br>18 – 24 *<br>20 – 22, 24 *	0 – 2…24 †<br>2 – 8, 20 *<br>2 – 12…22 †<br>4 – 8, 12, 14, 22, 24 †<br>4 – 10, 16…20 *<br>6 – 8, 12…22 †<br>6 – 10, 24 *<br>8 – 14 *<br>10 – 14 †<br>10 – 22 *<br>12 – 18 *<br>14 – 18 †<br>18 – 22 *	0 – 2…18 †<br>0 – 20…24 *<br>2 – 4, 10, 20…24 *<br>2 – 6, 8, 12…18 †<br>4 – 16 †<br>4 – 18…24 *<br>6 – 8, 14, 20…24 *<br>6 – 12, 16, 18 †<br>8 – 16 †<br>8 – 18… 24 *<br>10 – 16 †<br>10 – 20…24 *<br>12 – 16 †<br>12 – 18…24 *<br>14 – 16 *<br>16 – 18 *<br>18 – 20…24 *	0 – 2…24 †<br>2 – 14, 18…22 †<br>2 – 24 *<br>4 - 14, 18, 24 *<br>4 – 20, 22 †<br>6 – 8, 12, 16 *<br>6 – 14, 18…24 †<br>8 – 10, 14, 24 *<br>8 – 18…22 †<br>10 – 14, 18…24 †<br>10 – 16 *<br>12 – 20 †<br>12 – 22 *<br>16 – 20 *

The animals were treated with cadmium (Cd) in the diet at the concentration of 0, 1, or 5 mg/kg (Control, Cd$_1$, and Cd$_5$ groups) and/or 0.1% extract from the berries of *Aronia melanocarpa* L. (AM, Cd$_1$+AM, and Cd$_5$+AM groups) for 24 months. Data are shown as a median and minimum and maximum values for eight animals (except for seven females in the AE, Cd$_1$, and Cd$_5$ groups after 20, 22, and 24 months). Statistically significant differences (Kruskal–Wallis test) compared to: a—Control group; b—AM group; c—Cd$_1$ group; d—Cd$_1$+AM group; and e—Cd$_5$ group. Wilcoxon test was performed to compare data between particular two time points in the same animals during the 24-month study. * $p < 0.05$, † $p < 0.01$, and ‡ $p < 0.001$.

Table S8. The concentration of total protein (PCR) in the urine of female rats evaluated every other month during the 24-month study.

Months	Control	AM	Cd_1	Cd_1+AM	Cd_5	Cd_5+AM
0	3.457	3.464	3.706	3.451	3.755	3.566
	2.996 – 5.897	2.669 – 4.770	2.758 – 4.726	2.990 – 4.726	2.332 – 4.628	2.700 – 4.503
2	7.575	7.323	10.26	8.623	7.220	6.941
	6.200 – 15.96	4.653 – 39.63	8.190 – 22.09	6.308 – 24.71	3.381 – 18.31	1.722 – 15.99
4	9.005	5.190	10.24	8.347	23.01 ^{b*}	10.86
	4.391 – 18.96	3.104 – 20.35	3.055 – 26.13	2.687 – 10.90	4.868 – 33.42	5.875 – 27.12
6	6.932	9.714	22.46 ^{a†}	7.557 ^{c*}	15.79 ^{a*}	8.794
	3.107 – 10.56	1.963 – 13.50	8.580 – 27.85	4.416 – 16.33	7.045 – 20.35	3.129 – 26.57
8	9.280	10.21	45.34 ^{a*}	10.22 ^{c*}	32.94 ^{a*}	3.559 $^{c\ddagger\ e\ddagger}$
	4.227 – 15.12	3.034 – 32.08	29.34 – 78.80	4.046 – 20.79	30.01 – 48.38	2.689 – 5.184
10	12.55	8.712	38.32 $^{a*\ b\ddagger}$	14.34 ^{c*}	35.72 $^{a*\ b\ddagger}$	13.15 $^{c*\ e*}$
	6.768 – 16.56	6.096 – 12.14	26.16 – 59.32	8.064 – 18.96	28.80 – 43.36	7.680 – 18.96
12	10.80	6.435	45.42 $^{a*\ b\dagger}$	11.07 ^{c*}	46.10 $^{a*\ b\dagger}$	5.385 $^{c\ddagger\ e\ddagger}$
	6.272 – 12.28	4.206 – 12.68	39.28 – 68.00	8.240 – 15.44	38.44 – 57.68	2.614 – 7.626
14	10.46	7.500	44.82 $^{a*\ b\ddagger}$	11.82 ^{c*}	49.72 $^{a*\ b\ddagger}$	6.280 $^{c\ddagger\ e\ddagger}$
	8.040 – 11.20	4.560 – 11.04	38.96 – 51.52	5.760 – 14.36	37.40 – 64.12	4.080 – 9.200
16	11.30	9.900	43.44 $^{a*\ b\ddagger}$	11.27 ^{c*}	51.08 $^{a\dagger\ b\ddagger\ d\dagger}$	10.71 $^{c*\ e\dagger}$
	6.660 – 16.16	5.720 – 12.00	37.12 – 52.60	4.730 – 14.69	36.00 – 67.57	6.943 – 14.40
18	12.32	11.68	51.60 $^{a*\ b\dagger}$	11.98 ^{c*}	55.83 $^{a*\ b\dagger\ d*}$	11.48 $^{c\ddagger\ e\dagger}$
	8.680 – 16.36	6.560 – 15.48	36.96 – 87.12	9.440 – 16.20	43.04 – 67.20	4.320 – 14.80
20	11.76	14.20	42.96 ^{a*}	11.14 ^{c*}	57.22 $^{a\dagger\ b*\ d\dagger}$	11.46 $^{c*\ e\dagger}$
	8.120 – 16.28	8.760 – 16.40	38.05 – 81.64	10.44 – 16.20	42.96 – 66.32	5.880 – 19.56
22	11.48	12.20	60.36 ^{a*}	11.22 ^{c*}	57.62 $^{a*\ d\dagger}$	10.36 $^{c\dagger\ e\ddagger}$
	9.560 – 16.56	10.92 – 16.60	36.65 – 82.76	9.160 – 13.92	47.96 – 72.76	6.920 – 14.14
24	11.29	11.68	64.28 ^{a*}	8.986 ^{c‡}	63.09 $^{a*\ d\ddagger}$	10.71 $^{c\dagger\ e\dagger}$
	8.320 – 12.56	10.36 – 20.00	56.08 – 82.50	4.179 – 13.28	55.44 – 79.04	8.080 – 14.72
Friedman Test	$p < 0.001$	$p < 0.001$	$p < 0.001$	$p < 0.001$	$p < 0.001$	$p < 0.001$
Wilcoxon Test	0 – 2, 6…24 †<br>2 – 4 *<br>2 – 10 †<br>2 – 18 *<br>6 – 10…20 *<br>6 – 22, 24 †<br>8 – 10 †<br>14 – 18, 22 *<br>16 – 18…24 *	0 – 2, 10…18 †<br>0 – 4, 6, 8, 20…24 *<br>10 – 22, 24 *<br>12 – 18, 22, 24 *<br>14 – 18…24 *<br>16 – 18 †<br>16 – 22…24 *	0 – 2, 6…18 †<br>0 – 4, 20…24 *<br>2 – 8 †<br>2 – 20…24 *<br>4 – 6, 20…24 *<br>4 – 8…18 †<br>6 – 20…24 *<br>10 – 24 *<br>12 – 24 *<br>14 – 22, 24 *<br>16 – 22, 24 *<br>22 – 24 *	0 – 2, 8…24 †<br>0 – 4, 6 *<br>4 – 10, 14 †<br>4 – 10, 16…20 *<br>4 – 12, 18…22 *<br>16 – 18 *<br>18 – 24 *<br>20 – 24 *<br>22 – 24 †	0 – 2, 20…24 *<br>0 – 4…18 †<br>2 – 4, 20, 24 *<br>2 – 8…18 †<br>4 – 8, 20…24 *<br>4 – 10…18 †<br>6 – 8…18 †<br>6 – 20…24 *<br>8 – 12…18 †<br>8 – 14, 16, 20…24 *<br>10 – 12…18 †<br>10 – 20…24 *<br>12 – 18 †<br>12 – 22, 24 *	0 – 2 *<br>0 – 4, 6, 10, 14…24 †<br>2 – 8 *<br>4 – 8 †<br>4 - 12, 14 *<br>6 – 8 *<br>8 – 10, 14…24 †<br>10 – 12 †<br>10 – 14 *<br>12 – 16…24 †<br>14 – 16, 18 *<br>14 – 20…24 †

The animals were treated with cadmium (Cd) in the diet at the concentration of 0, 1, or 5 mg/kg (Control, Cd_1, and Cd_5 groups) and/or 0.1% extract from the berries of *Aronia melanocarpa* L. (AM, Cd_1+AM, and Cd_5+AM groups) for 24 months. Data are shown as a median and minimum and maximum values for eight animals (except for seven females in the AE, Cd_1, and Cd_5 groups after 20, 22, and 24 months). Statistically significant differences (Kruskal–Wallis test) compared to: a—Control group; b—AM group; c—Cd_1 group; d—Cd_1+AM group; and e—Cd_5 group. Wilcoxon test was performed to compare data between particular two time points in the same animals during the 24-month study. * $p < 0.05$, $^{\dagger}p < 0.01$, and $^{\ddagger}p < 0.001$.

Table S9. The concentration of creatinine in the serum and urine and creatinine clearance in female rats.

Group	Experiment Duration<br>3 Months	 10 Months	 17 Months	 24 Months
		**Creatinine in the Serum (mg/100 mL)**		
Control	0.780	0.742	0.648	0.862
	0.678 – 0.847	0.645 – 0.774	0.537 – 0.806	0.646 – 0.985
AM	0.758	0.710	0.537	0.708
	0.678 – 0.885	0.613 – 0.903	0.358 – 0.866	0.554 – 0.923
Cd_1	0.692	0.726	0.761	1.015
	0.615 – 0.769	0.645 – 0.839	0.478 – 1.134	0.646 – 1.108
Cd_1+AM	0.769	0.807	0.552	0.708
	0.731 – 0.846	0.704 – 0.936	0.418 – 0.866	0.677 – 1.262
Cd_5	0.750 $^{b\dagger\ d\dagger}$	0.874	0.885	1.126 $^{b\dagger\ d*}$
	0.654 – 0.885	0.667 – 1.412	0.537 – 1.060	0.973 – 1.277
Cd_5+AM	0.556 $^{a\ddagger\ e\dagger}$	0.784	0.619 ^{e†}	0.687 $^{c*\ e\ddagger}$
	0.540 – 0.667	0.745 – 0.902	0.358 – 0.716	0.523 – 0.800
		**Creatinine in the Urine (mg/mL)**		
Control	1.491	1.046	0.941	0.703
	0.545 – 2.564	0.662 – 1.339	0.484 – 5.210	0.348 – 2.754
AM	1.391	0.709	0.637	0.596
	0.764 – 2.364	0.554 – 1.077	0.274 – 2.081	0.422 – 1.032
Cd_1	1.121	0.928	1.070	0.816
	0.420 – 1.677	0.652 – 1.464	0.404 – 4.404	0.438 – 1.219
Cd_1+AM	0.779	0.900	0.720	0.891
	0.435 – 1.516	0.371 – 3.757	0.229 – 1.421	0.469 – 2.047
Cd_5	0.976	0.923	0.617	0.737
	0.516 – 1.500	0.592 – 2.197	0.325 – 0.983	0.272 – 0.951
Cd_5+AM	1.260	0.675	0.667	0.726
	0.796 – 1.944	0.282 – 2.169	0.500 – 1.400	0.418 – 1.508
		**Creatinine Clearance (mL/min)**		
Control	1.346	1.060	0.949	0.984
	1.015 – 1.592	0.832 – 1.538	0.505 – 1.001	0.820 – 1.203
AM	1.208	0.927	0.909	0.931
	0.652 – 1.484	0.852 – 1.464	0.610 – 1.519	0.725 – 1.259
Cd_1	1.289	0.943	0.950	0.982
	0.757 – 2.987	0.799 – 1.292	0.410 – 1.476	0.847 – 1.036
Cd_1+AM	1.117	0.967	1.030	1.306
	0.769 – 1.863	0.604 – 1.616	0.736 – 1.813	0.519 – 1.693
Cd_5	1.203	0.988	0.613 $^{a\dagger\ d\ddagger}$	0.764 ^{a†}
	0.960 – 2.348	0.732 – 1.711	0.374 – 0.799	0.425 – 0.798
Cd_5+AM	1.205	1.095	0.860 ^{e*}	1.582 ^{e†}
	0.798 – 1.829	0.874 – 1.277	0.625 – 1.357	0.785 – 2.034

The animals were treated with cadmium (Cd) in the diet at the concentration of 0, 1, or 5 mg/kg (Control, Cd_1, and Cd_5 groups) and/or 0.1% extract from the berries of *Aronia melanocarpa* L. (AM, Cd_1+AM, and Cd_5+AM groups) for 3, 10, 17, and 24 months. Data are shown as a median and minimum and maximum values for eight animals (except for seven females in the AM, Cd_1, and Cd_5 groups after 24 months). Statistically significant differences (Kruskal–Wallis test) compared to: a—Control group; b—AM group; c—Cd_1 group; d—Cd_1+AM group; and e—Cd_5 group, where * $p < 0.05$, $^{\dagger} p < 0.01$, and $^{\ddagger} p < 0.001$, are marked.

Table S10. The concentration of urea and uric acid in the serum of female rats.

Group	3 Months	10 Months	17 Months	24 Months
	**Experiment Duration**			
	**Uric Acid (mg/100 mL)**			
Control	3.110	2.360	3.840	3.380
	2.680 – 3.630	2.000 – 2.680	3.440 – 4.690	3.310 – 4.900
AM	3.315	2.540	3.970	3.200
	2.950 – 3.630	2.100 – 3.050	3.590 – 4.940	2.950 – 4.500
Cd_1	4.340 $^{a‡\ b†}$	2.440	3.640	3.450
	4.020 – 4.630	2.120 – 2.520	3.000 – 4.030	3.160 – 4.430
Cd_1+AM	4.210 $^{a‡\ b†}$	2.460	4.414	3.750
	3.680 – 5.370	2.260 – 2.700	3.310 – 4.630	3.200 – 4.800
Cd_5	3.380 $^{c*\ d*}$	2.750	4.140	4.400 $^{a*\ b†}$
	3.150 – 3.590	2.350 – 3.430	3.160 – 4.750	3.500 – 7.500
Cd_5+AM	3.420	2.550	4.060	3.700
	3.200 – 4.000	2.260 – 2.770	3.530 – 4.840	3.100 – 5.500
	**Urea (mg/100 mL)**			
Control	26.30	32.54	28.26	24.57
	20.18 – 32.40	10.09 – 44.40	23.28 – 35.80	17.24 – 33.62
AM	23.47	40.52	24.56	17.24
	16.20 – 30.75	15.95 – 52.16	19.40 – 31.90	11.20 – 40.00
Cd_1	37.22	26.51	26.79	38.02
	26.29 – 47.88	20.26 – 38.79	24.60 – 33.20	34.90 – 48.90
Cd_1+AM	38.38 ^{b*}	38.80	34.50	21.88
	10.61 – 47.18	4.810 – 49.57	16.80 – 40.80	18.75 – 25.86
Cd_5	32.16	40.73	37.87 $^{a*\ b†\ c*}$	53.62 $^{a*\ b†\ d†}$
	14.32 – 42.72	29.31 – 52.58	31.40 – 46.55	41.14 – 79.16
Cd_5+AM	21.92 $^{c*\ d†}$	35.35	32.35	20.10 $^{c*\ e†}$
	13.85 – 26.06	4.810 – 42.67	21.40 – 38.90	17.71 – 34.04

The animals were treated with cadmium (Cd) in the diet at the concentration of 0, 1, or 5 mg/kg (Control, Cd_1, and Cd_5 groups) and/or 0.1% extract from the berries of *Aronia melanocarpa* L. (AM, Cd_1+AM, and Cd_5+AM groups) for 3, 10, 17, and 24 months. Data are shown as a median and minimum and maximum values for eight animals (except for seven females in the AM, Cd_1, and Cd_5 groups after 24 months). Statistically significant differences (Kruskal–Wallis test) compared to: a—Control group; b—AM group; c—Cd_1 group; d—Cd_1+AM group; and e—Cd_5 group, where * $p < 0.05$, † $p < 0.01$, and ‡ $p < 0.001$, are marked.

Table S11. The concentrations of urea and uric acid in the urine of female rats.

Group	Experiment Duration<br>3 Months	10 Months	17 Months	24 Months
		**Uric Acid (mg/24 h)**		
Control	21.43	23.85	18.01	33.99
	12.42 – 44.00	14.80 – 32.27	16.52 – 24.60	18.71 – 76.35
AM	20.22	35.77	24.20	39.24
	8.300 – 35.85	28.35 – 47.88	3.860 – 39.16	9.650 – 68.88
Cd_1	31.02	26.25	32.35	21.96
	12.73 – 71.10	17.40 – 35.91	3.200 – 41.50	13.89 – 42.39
Cd_1+AM	29.32	23.52	35.95	28.68
	26.25 – 48.43	12.28 – 41.21	11.77 – 66.41	8.253 – 51.99
Cd_5	35.21	21.88	32.68	27.37
	24.5558.56	14.61 – 48.20	14.18 – 52.44	19.86 – 31.26
Cd_5+AM	26.72	26.38	27.86	34.12
	15.63 – 39.84	10.56 – 77.53	9.510 – 47.76	22.53 – 37.97
		**Urea (mg/24 h)**		
Control	41.85	35.03	57.52	36.93
	18.60 – 72.00	22.80 – 43.40	29.52 – 68.58	21.82 – 74.82
AM	40.12	47.80	43.55	43.16
	19.25 – 50.25	26.95 – 81.90	24.00 – 69.50	13.29 – 83.44
Cd_1	51.30	31.32	57.37	39.46
	16.75 – 99.00	22.40 – 37.80	22.06 – 86.40	24.27 – 64.94
Cd_1+AM	58.10	34.69	42.17	59.65
	24.60 – 86.40	16.70 – 56.64	22.13 – 72.73	14.76 – 82.61
Cd_5	50.38	36.96	53.96	41.76
	33.50 – 81.60	17.10 – 53.80	38.97 – 92.42	33.58 – 72.85
Cd_5+AM	42.95	43.67	43.67	51.27
	20.00 – 52.20	22.89 – 56.52	23.52 – 70 04	26.52 – 100.1

The animals were treated with cadmium (Cd) in the diet at the concentration of 0, 1, or 5 mg/kg (Control, Cd_1, and Cd_5 groups) and/or 0.1% extract from the berries of *Aronia melanocarpa* L. (AM, Cd_1+AM, and Cd_5+AM group) for 3, 10, 17, and 24 months. Data are shown as a median and minimum and maximum values for eight animals (except for seven females in the AM, Cd_1, and Cd_5 groups after 24 months). There were no statistically significant differences (Kruskal–Wallis test) between the experimental groups.

Table S12. The concentration of cadmium (Cd) in the urine of female rats evaluated every other month during the 24-month study.

Months	Control	AM	Cd_1	Cd_1+AM	Cd_5	Cd_5+AM
0	0.1263	0.1157	0.1333	0.1216	0.1170	0.1323
	0.0300 - 0.1749	0.0886 – 0.1764	0.0600 – 0.1766	0.0393 – 0.1794	0.0600 – 0.1794	0.0812 – 0.1467
2	0.1376	0.1337	0.3024	0.3818 ^{a*}	0.4534 $^{a\ddagger\, b*}$	0.4706 $^{a\ddagger\, b\dagger}$
	0.0950 - 0.2752	0.1040 – 0.3917	0.1422 – 0.3462	0.2790 – 0.4942	0.1664 – 0.5727	0.2584 – 0.6948
4	0.1342	0.1259	0.2398	0.2474	0.4378 $^{a\dagger\, b\dagger}$	0.4733 $^{a\dagger\, b*}$
	0.0677 – 0.2894	0.1029 – 0.1570	0.1187 – 0.4146	0.1418 – 0.4231	0.2094 – 0.6720	0.1610 – 0.7368
6	0.1244	0.1421	0.2311 ^{a†}	0.2162	0.2599 $^{a\ddagger\, b\dagger\, d*}$	0.2392 $^{a\dagger\, b*}$
	0.0620 – 0.1876	0.1044 – 0.1908	0.1485 – 0.3397	0.1519 – 0.3184	0.2327 – 0.4169	0.1696 – 0.6054
8	0.1136	0.1161	0.2481 ^{b*}	0.2476 $^{a*\, b\dagger}$	0.3224 $^{a\dagger\, b\dagger}$	0.2876 $^{a*\, b\dagger}$
	0.0652 – 0.1738	0.0646 – 0.1690	0.1396 – 0.4467	0.2125 – 0.5917	0.1862 – 0.4342	0.2376 – 0.3512
10	0.1129	0.1439	0.2121	0.2188	0.3570 $^{a\ddagger\, b\dagger}$	0.3957 $^{a\ddagger\, b\ddagger}$
	0.1015 – 0.1601	0.1127 – 0.1605	0.1114 – 0.3586	0.1609 – 0.3720	0.2120 – 0.5084	0.2879 – 0.6738
12	0.1446	0.1564	0.2403	0.2848 ^{a†}	0.3462 $^{a\dagger\, b*}$	0.3622 $^{a\ddagger\, b*}$
	0.0938 – 0.1883	0.0833 – 0.2836	0.1912 – 0.4959	0.2256 – 0.4953	0.2024 – 0.6804	0.2053 – 0.4630
14	0.1549	0.1996	0.2700	0.2972 ^{a*}	0.4798 $^{a\dagger\, b\dagger}$	0.5580 $^{a\ddagger\, b\dagger}$
	0.0870 – 0.2560	0.0893 – 0.2553	0.1850 – 0.3110	0.2790 – 0.6836	0.2032 – 0.7207	0.2338 – 0.9776
16	0.1634	0.1697	0.2867	0.3031	0.4968 $^{a\dagger\, b*}$	0.6167 $^{a\ddagger\, b\dagger\, e*}$
	0.0683 – 0.2176	0.0883 – 0.3840	0.1255 – 0.6386	0.2053 – 0.9170	0.2867 – 0.9785	0.3414 – 0.8911
18	0.1335	0.1472	0.2180	0.2256	0.3936 $^{a\dagger\, b\dagger}$	0.4329 $^{a\ddagger\, b\ddagger}$
	0.1010 – 0.1829	0.1163 – 0.1841	0.1227 – 0.4043	0.1774 – 0.3824	0.1860 – 0.4897	0.2423 – 0.6143
20	0.1310	0.1515	0.2019	0.2107	0.3485 $^{a\ddagger\, b\dagger}$	0.4068 $^{a\ddagger\, b\ddagger\, e*}$
	0 0906 – 0.1898	0.0768 – 0.2050	0.1704 – 0.2918	0.1579 – 0.3246	0.2523 – 0.4841	0.3272 – 0.5538
22	0.1382	0.1552	0.2230	0.3061 $^{a\dagger\, b*}$	0.3345 $^{a\dagger\, b*}$	0.4859 $^{a\ddagger\, b\dagger\, e*}$
	0.1217 – 0.2770	0.1251 – 0.2168	0.1565 – 0.2864	0.2584 – 0.4623	0.2034 – 0.4645	0.3927 – 0.8177
24	0.1374	0.1304	0.1958	0.2050	0.4206 $^{a\ddagger\, b\ddagger}$	0.4927 $^{a\ddagger\, b\ddagger\, e*}$
	0.0764 – 0.1937	0.1118 – 0.1618	0.1795 – 0.2451	0.1794 – 0.2531	0.3110 – 0.4695	0.4308 – 0.6616
Friedman Test	$p = 0.790$	$p < 0.05$	$p = 0.063$	$p < 0.001$	$p < 0.001$	$p < 0.001$
Wilcoxon Test		2 – 8 *<br>4 – 22 *<br>6 – 8 *<br>8 – 10, 14…18, 22, 24 *<br>10 – 22 †<br>12 – 24 *		0 – 2, 8…18, 22, 24 †<br>0 – 4, 6, 20 *<br>2 – 4, 18, 22 *<br>2 - 6, 10, 20, 24 †<br>6 – 14 *<br>6 – 22 †<br>8 – 24 *<br>10 – 14 †<br>10 – 16, 22 *<br>12 – 20, 24 *<br>14 – 18 *<br>14 – 20, 24 †<br>16 – 20, 24 *<br>18 – 22 *<br>20 – 22 *<br>22 – 24 †	0 – 2…18 †<br>0 – 20…24 *<br>2 – 6 *<br>6 – 14, 16, 20, 24 *<br>8 – 24 *<br>20 – 24 *	0 – 2…24 †<br>2 – 6, 8 *<br>4 – 6 *<br>6 – 14, 24 *<br>6 – 16 †<br>8 – 10, 14, 18...22 *<br>8 – 16, 24 †<br>12 – 14, 16, 24 *<br>16 – 20 *

The animals were treated with cadmium (Cd) in the diet at the concentration of 0, 1, or 5 mg/kg (Control, Cd_1, and Cd_5 groups) and/or 0.1% extract from the berries of *Aronia melanocarpa* L. (AM, Cd_1+AM, and Cd_5+AM groups) for 24 months. Data are shown as a median and minimum and maximum values for eight animals (except for seven females in the AE, Cd_1, and Cd_5 groups after 20, 22, and 24 months). Statistically significant differences (Kruskal–Wallis test) compared to: a—Control group; b—AM group; c—Cd_1 group; d—Cd_1+AM group; and e—Cd_5 group. Wilcoxon test was performed to compare data between particular two time points in the same animals during the 24-month study. * $p < 0.05$, † $p < 0.01$, and ‡ $p < 0.001$.

Table S13. The absolute and the relative weight of the left kidney of rats.

Group	Experiment Duration			
	3 Months	10 Months	17 Months	24 Months
**Absolute Weight of the Kidney (g)**				
Control	0.9420<br>0.8595 – 1.0100	0.9292<br>0.8075 – 1.0274	1.1014<br>0.9994 – 1.4990	1.3065<br>1.1163 – 2.0292
AM	1.0005<br>0.8176 – 1.0691	0.9575<br>0.8781 – 1.0104	1.2544<br>0.8618 – 1.5903	1.1884<br>0.9532 – 1.9371
Cd_1	0.8962<br>0.4648 – 0.9746	0.9794<br>0.8703 – 1.0617	1.1659<br>0.9084 – 1.8407	1.3195<br>1.2592 – 1.4661
Cd_1+AM	0.9098<br>0.8226 – 1.0242	0.9848<br>0.8094 – 1.0719	1.1322<br>0.9154 – 1.2132	1.3463<br>1.1764 – 1.6897
Cd_5	0.9021<br>0.8628 – 1.0535	1.0326<br>0.5309 – 1.1813	1.0986<br>1.0553 – 1.4936	1.3646<br>1.2528 – 1.6199
Cd_5+AM	0.8806<br>0.7716 – 1.0770	0.9223<br>0.8183 – 1.0479	1.1244<br>1.0384 – 1.3111	1.1801<br>1.0834 – 1.2960
**Relative Weight of the Kidney (g/100 g b.w.)**				
Control	0.3032<br>0.2732 – 0.3222	0.2104<br>0.1963 – 0.2581	0.2209<br>0.1562 – 0.3407	0.1937<br>0.1786 – 0.3624
AM	0.3128<br>0.2937 – 0.3300	0.2170<br>0.1792 – 0.2536	0.2460<br>0.1413 – 0.3181	0.2246<br>0.1467 – 0.3522
Cd_1	0.3054<br>0.1592 – 0.3387	0.2200<br>0.2868 – 0.2485	0.2451<br>0.1748 – 0.4184	0.1884<br>0.1672 – 0.2865
Cd_1+AM	0.3117<br>0.2856 – 0.3556	0.2153<br>0.1655 – 0.2666	0.2246<br>0.1695 – 0.2639	0.2666<br>0.2262 – 0.3096
Cd_5	0.3005<br>0,2818 – 0.4130	0.2501<br>0.1144 – 0.2774	0.2386<br>0.1505 – 0.4268	0.2481<br>0.2085 – 0.,2793
Cd_5+AM	0.2825<br>0.2660 – 0.3121	0.2272<br>0.1819 – 0.2501	0.2323<br>0.1783 – 0.2914	0.2123<br>0.1708 – 0.2875

The animals were treated with cadmium (Cd) in the diet at the concentration of 0, 1, or 5 mg/kg (Control, Cd_1, and Cd_5 groups) and/or 0.1% extract from the berries of *Aronia melanocarpa* L. (AM, Cd_1+AM, and Cd_5+AM groups) for 3, 10, 17, and 24 months. Data are shown as a median and minimum and maximum values for eight animals (except for seven females in the AM, Cd_1, and Cd_5 groups after 24 months). There were no statistically significant differences (Kruskal-Wallis test) between the experimental groups.

Table S14. The concentrations of chemerin, macrophage inflammatory protein 1a (MIP1a), and Bcl2-associated X protein (Bax) in the kidney of female rats.

Group	Experiment Duration			
	**3 Months**	**10 Months**	**17 Months**	**24 Months**
	**Chemerin (pg/mg Protein)**			
Control	29.82	14.68	16.09	16.41
	26.91 – 39.83	11.78 – 19.82	14.90 – 17.66	14.25 – 17.59
AM	22.84	14.01	14.90	15.62
	10.91 – 33.76	7.282 – 26.61	10.93 – 19.28	12.98 – 19.15
Cd$_1$	28.12	16.73	20.54	38.85 ^{a*}
	21.67 – 86.31	10.81 – 56.95	18.10 – 22.05	28.27 – 40.15
Cd$_1$+AM	22.02	12.35	16.81	11.75 ^{c‡}
	7.063 – 32.88	7.414 – 25.75	15.88 – 17.86	9.142 – 13.92
Cd$_5$	32.35	18.78	22.22 $^{a\dagger\ b\dagger}$	37.69 $^{a*\ d\ddagger}$
	23.04 – 47.10	11.32 – 38.81	19.32 – 23.71	35.44 – 41.36
Cd$_5$+AM	11.56 $^{a\dagger\ c\dagger\ e\dagger}$	2.887 $^{a*\ b*\ c\ddagger\ d*\ e\ddagger}$	10.43 $^{c\ddagger\ e\ddagger}$	10.86 $^{c\ddagger\ e\ddagger}$
	6.634 – 19.81	1.174 – 5.572	9.596 – 12.57	9.288 – 11.47
	**MIP1a (ng/mg Protein)**			
Control	2.168	1.138	1.223	0.837
	1.162 – 3.504	0.840 – 2.996	0.874 – 1.826	0.683 – 1.196
AM	1.652	1.065	0.587	0.266
	0.829 – 2.250	0.912 – 2.478	0.269 – 0.977	0.128 – 0.425
Cd$_1$	1.984	1.390	0.941	1.000
	0.919 – 2.536	0.920 – 3.180	0.585 – 1.604	0.792 – 2.020
Cd$_1$+AM	1.441	0.754	0.260 $^{a\ddagger\ c*}$	0.190 $^{a*\ c\ddagger}$
	0.761 – 2.281	0.647 – 1.150	0.036 – 0.331	0.144 – 0.213
Cd$_5$	0.930 $^{a\ddagger\ c*}$	2.172 ^{d‡}	1.618 $^{b*\ d\ddagger}$	1.266
	0.721 – 1.125	1.776 – 2.462	1.218 – 2.604	0.942 – 2.076
Cd$_5$+AM	0.890 $^{a\ddagger\ c\dagger}$	0.717 $^{a*\ c*\ e\ddagger}$	0.316 $^{a\dagger\ e\dagger}$	0.169 $^{a*\ e\ddagger}$
	0.751 – 0.968	0.567 – 0.859	0.094 – 0.520	0.102 – 0.262
	**Bax (ng/mg Protein)**			
Control	5.412	2.651	2.661	1.938
	3.381 – 6.340	0.465 – 4.571	1.728 – 3.572	1.307 – 2.420
AM	3.830	2.974	1.853	1.387
	3.023 – 6.722	1.785 – 3.605	0.914 – 2.550	1.181 – 2.502
Cd$_1$	5.607	3.075	5.792	2.231
	3.293 – 7.317	2.393 – 6.568	3.637 – 12.47	1.617 – 3.876
Cd$_1$+AM	3.120	1.924 ^{c*}	1.052 ^{c‡}	1.096 ^{c*}
	1.638 – 5.257	1.046 – 2.597	0.532 – 1.607	0.922 – 1.877
Cd$_5$	2.780	19.990	3.839 ^{d†}	2.732 $^{a*\ b*\ d\ddagger}$
	2.456 – 3.594	1.718 – 2.215	2.552 – 4.915	2.442 – 5.216
Cd$_5$+AM	1.469 $^{a\ddagger\ b\dagger\ c\ddagger}$	1.195 $^{b\dagger\ c\ddagger}$	1.186 $^{c\ddagger\ e\dagger}$	1.452 ^{e†}
	0.736 – 1.614	1.031 – 2.163	0.911 – 1.668	1.004 – 2.002

The animals were treated with cadmium (Cd) in the diet at the concentration of 0, 1, or 5 mg/kg (Control, Cd$_1$, and Cd$_5$ groups) and/or 0.1% extract from the berries of *Aronia melanocarpa* L. (AM, Cd$_1$+AM, and Cd$_5$+AM groups) for 3, 10, 17, and 24 months. Data are shown as a median and minimum and maximum values for eight animals (except for seven females in the AM, Cd$_1$, and Cd$_5$ groups after 24 months). Statistically significant differences (Kruskal–Wallis test) compared to: a—Control group; b—AM group; c—Cd$_1$ group; d—Cd$_1$+AM group; and e—Cd$_5$ group, where * $p < 0.05$, $^{\dagger}p < 0.01$, and $^{\ddagger}p < 0.001$, are marked.

Table S15. The concentration of cadmium (Cd) in the blood, urine, and kidney of female rats [1,2].

Group	Experiment Duration: 3 Months	10 Months	17 Months	24 Months
**Blood (μg Cd/L)**				
Control	0.0691	0.0860	0.0744	0.0834
	0.0330 – 0.1110	0.0360 – 0.1290	0.0480 – 0.0960	0.0690 – 0.0990
AM	0.0752	0.0802	0.0718	0.0861
	0.0500 – 0.0960	0.0330 – 0.1360	0.0103 – 0.0950	0.0700 – 0.0970
Cd_1	0.1884	0.1792	0.2425	0.2330
	0.1320 – 0.2460	0.1030 – 0.3060	0.1980 – 0.3240	0.1850 – 0.2840
	↑ 2.7x	↑ 2.1x	↑ 3.3x	↑ 2.8x
Cd_1+AM	0.1877	0.1844	0.2375	0.2189
	0.1290 – 0.2480	0.1170 – 0.2760	0.1610 – 0.3050	0.1900 – 0.3000
	↑ 2.7x	↑ 2.1x	↑ 3.2x	↑ 2.6x
Cd_5	1.0236	0.9394	1.0339	1.0467
	0.7390 – 1.3320	0.7350 – 1.1220	0.9400 – 1.1310	0.8250 – 1.2010
	↑ 14.8x	↑ 10.9x	↑ 13.9x	↑ 12.6x
Cd_5+AM	0.8298	0.7948	0.9319	0.8201
	0.6200 – 1.0640	0.5840 – 0.9960	0.7920 – 1.1250	0.6090 – 1.1490
	↑ 12x ↘ 19%	↑ 9.2x ↘ 15%	↑ 12.5x ↘ 10%	↑ 9.8x ↘ 22%
**Urine (μg Cd/g Creatinine)**				
Control	0.1387	0.1304	0.1491	0.1337
	0.1032 – 0.2013	0.0944 – 0.1722	0.1172 – 0.1889	0.0764 – 0.1937
AM	0.1321	0.1364	0.1445	0.1357
	0.1129 – 0.1507	0.1119 – 0.1558	0.0730 – 0.1712	0.1118 – 0.1618
Cd_1	0.2184	0.1809	0.2096	0.2053
	0.1810 – 0.2509	0.0852 – 0.2558	0.1069 – 0.2820	0.1795 – 0.2451
	↑ 57%	↑ 39%	↑ 41%	↑ 54%
Cd_1+AM	0.2193	0.1913	0.2143	0.2084
	0.1349 – 0.2762	0.1392 – 0.2483	0.1471 – 0.3538	0.1794 – 0.2531
	↑ 58%	↑ 47%	↑ 44%	↑ 56%
Cd_5	0.5008	0.4002	0.4737	0.4104
	0.4183 – 0.6016	0.2839 – 0.6949	0.3369 – 0.6467	0.3110 – 0.4695
	↑ 3.6x	↑ 3.1x	↑ 3.2x	↑ 3.1x
Cd_5+AM	0.6064	0.4997	0.5773	0.4994
	0.4833 – 0.8197	0.3640 – 0.6812	0.4206 – 0.7481	0.4308 – 0.6616
	↑ 4.4x ↗ 21%	↑ 3.8x ↗ 25%	↑ 3.9x ↗ 22%	↑ 3.7x ↗ 22%
**Kidney (μg Cd/g)**				
Control	0.0385	0.0505	0.0467	0.0844
	0.0283 – 0.0618	0.0385 – 0.0619	0.0364 – 0.0644	0.0452 – 0.1382
AM	0.0355	0.0491	0.0555	0.0930
	0.0282 – 0.0425	0.0308 – 0.0614	0.0454 – 0.0677	0.0599 – 0.1396
Cd_1	0.3482	1.1342	1.2126	1.9807
	0.2626 – 0.4472	0.8769 – 1.4298	0.7714 – 1.8262	1.0909 – 2.8322
	↑ 9x	↑ 22x	↑ 26x	↑ 23x
Cd_1+AM	0.2475	1.0277	1.3048	2.0533
	0.1666 – 0.3018	0.7600 – 1.1772	1.1193 – 1.5348	1.2735 – 2.9219
	↑ 6.4x ↘ 29%	↑ 20x ↘ 9.5%	↑ 28x	↑ 24x
Cd_5	1.3714	4.8425	10.7680	8.0093
	0.9739 – 1.6725	4.2892 – 6.1790	8.4189 – 14.8705	6.5652 – 8.8627
	↑ 36x	↑ 96x	↑ 231x	↑ 95x
Cd_5+AM	1.1805	4.5712	9.6644	7.3042
	0.9976 – 1.4006	3.5350 – 5.1680	8.2900 – 11.7654	5.7523 – 8.6859
	↑ 31x ↘ 14%	↑ 91x ↘ 5.6%	↑ 207x ↘ 10%	↑ 87x ↘ 8.8%

The animals were treated with cadmium (Cd) in the diet at the concentration of 0, 1, or 5 mg/kg (Control, Cd_1, and Cd_5 groups) and/or 0.1% extract from the berries of *Aronia melanocarpa* L. (AM, Cd_1+AM, and Cd_5+AM groups) for 3, 10, 17, and 24 months. Data are shown as a mean and minimum and maximum values for eight animals (with the exception of seven females in the AM, Cd_1,

and Cd_5 groups after 24 months). The factors or percentages of changes compared to the control group (↑, increase) or the adequate group treated with Cd alone (↘, decrease; ↗, increase) are indicated if $p < 0.05$. [1] prepared based on Brzóska, M.M.; Gałażyn-Sidorczuk, M.; Jurczuk, M.; Tomczyk, M. Protective effect of *Aronia melanocarpa* polyphenols on cadmium accumulation in the body: A study in a rat model of human exposure to this metal. *Curr. Drug Targets* **2015**, *16*, 1470–1487. https://doi.org/10.2174/1389450116666150102121708. [2] Cd concentration in the blood and urine was also presented in the Supplementary Material for the article Mężyńska, M.; Brzóska, M.M.; Rogalska, J.; Piłat-Marcinkiewicz, B. Extract from *Aronia melanocarpa* L. berries prevents cadmium-induced oxidative stress in the liver: A study in a rat model of low-level and moderate lifetime human exposure to this toxic metal. *Nutrients* **2019**, *11*, 21. https://doi.org/10.3390/nu11010021.

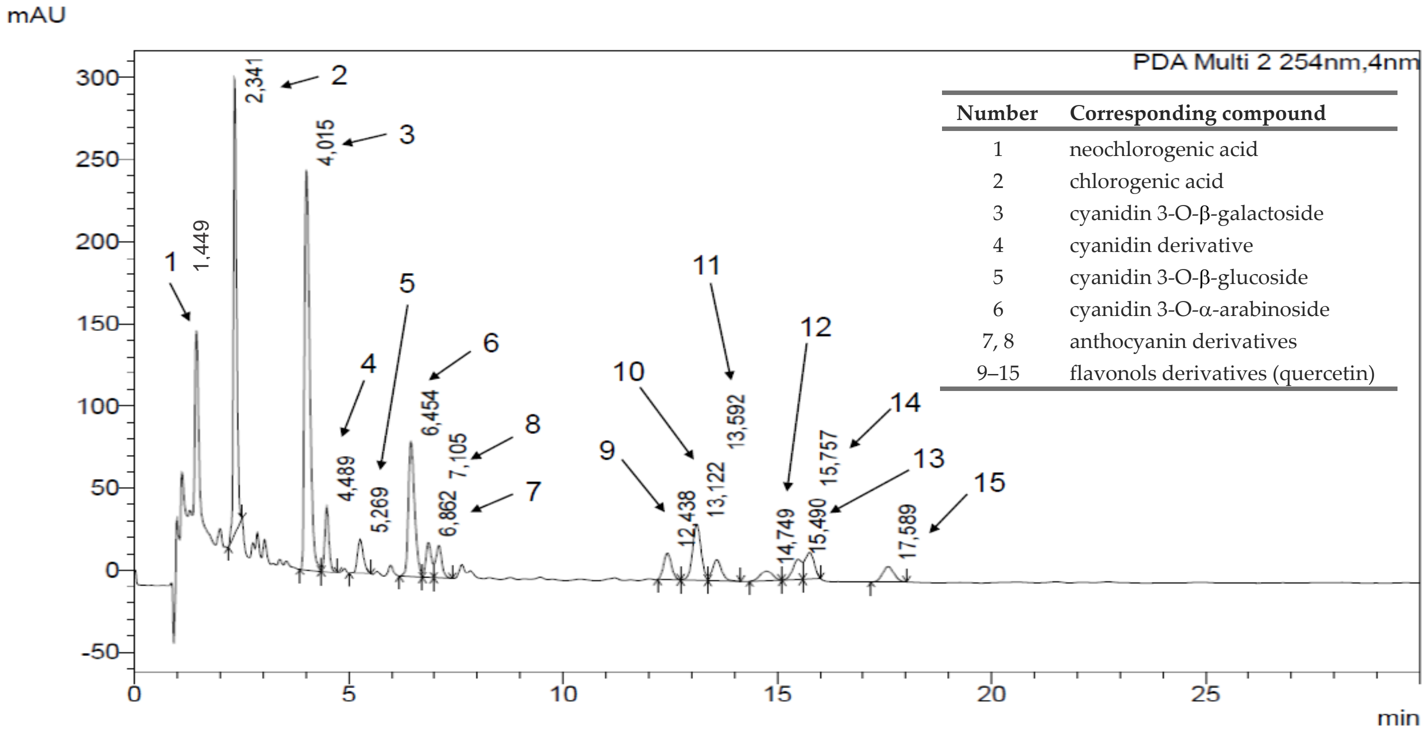

Number	Corresponding compound
1	neochlorogenic acid
2	chlorogenic acid
3	cyanidin 3-O-β-galactoside
4	cyanidin derivative
5	cyanidin 3-O-β-glucoside
6	cyanidin 3-O-α-arabinoside
7, 8	anthocyanin derivatives
9–15	flavonols derivatives (quercetin)

Figure S1. Ultra Performance Liquid Chromatography (UPLC) polyphenolic profile of the *Aronia melanocarpa* L. berry extract. The profile of the extract was investigated and presented in Brzóska, M.M.; Rogalska, J.; Gałażyn-Sidorczuk, M.; Jurczuk, M.; Roszczenko, A.; Tomczyk, M. Protective effect of *Aronia melanocarpa* polyphenols against cadmium-induced disorders in bone metabolism: A study in a rat model of lifetime human exposure to this heavy metal. *Chem. Biol. Interact*. **2015**, *229*, 132–146. https://doi.org/10.1016/j.cbi.2015.01.031.
